# Tidal tomography reveals a thermal anomaly beneath Mars’s crustal dichotomy

**DOI:** 10.1038/s41586-026-10893-x

**Published:** 2026-08-26

**Authors:** Alexander Berne, Nicholas Wagner, Isamu Matsuyama, Sander Goossens, Antonio Genova, Marc Rovira-Navarro, Amirhossein Bagheri, Chuan Qin, Angela Marusiak, Douglas Hemingway, Harriet C. P. Lau, Kar Wai Cheng, Shijie Zhong, Francis Nimmo

**Affiliations:** 1 Lunar and Planetary Laboratory, University of Arizona, Tucson, AZ USA; 2 Department of Earth, Environmental and Planetary Sciences, Brown University, Providence, RI USA; 3 Planetary Geology, Geophysics and Geochemistry Laboratory, NASA Goddard Space Flight Center, Greenbelt, MD USA; 4 Department of Mechanical and Aerospace Engineering, Sapienza University of Rome, Rome, Italy; 5 Department of Geoscience and Engineering, Delft University of Technology, Delft, The Netherlands; 6 Division of Geological and Planetary Sciences, California Institute of Technology, Pasadena, CA USA; 7 Department of Earth, Planetary, and Space Sciences, University of California, Los Angeles (UCLA), Los Angeles, CA USA; 8 Institute for Geophysics, Jackson School of Geosciences, University of Texas at Austin, Austin, TX USA; 9 Institute of Earth Sciences, Academia Sinica, Taipei, Taiwan; 10 Department of Physics, University of Colorado Boulder, Boulder, CO USA; 11 Department of Earth and Planetary Sciences, University of California, Santa Cruz, Santa Cruz, CA USA

**Keywords:** Geodynamics, Tectonics, Geophysics, Inner planets

## Abstract

Mars undergoes seasonal tidal forcing as a result of its eccentric orbit and the tilt of its rotation axis relative to the Sun^1^. Response to this forcing produces temporal variations in the Martian gravity field and is sensitive to the internal structure of the planet^2–4^. Using tracking data from the Mars Global Surveyor (MGS), Mars Odyssey (ODY) and Mars Reconnaissance Orbiter (MRO) spacecraft^5–7^, we demonstrate that degree-3 components of the time-variable gravity field of Mars differ by up to 300% from predictions for a spherically symmetric planet^8–10^. These deviations can be explained if the effective shear modulus of the mantle varies by >20% over a roughly north–south pattern that closely aligns with the surface expression of the Martian crustal dichotomy^11^. On the basis of this correlation, we infer preservation of a 200–400 °C thermal anomaly in the present-day mantle below the southern highlands of Mars. This temperature variation could reflect regional mantle convection^12,13^ or insulation by the thick southern highlands crust of Mars that has persisted over several billion years^14,15^.

## Abstract (web summary)

Analysis of tracking data from Mars orbiters reveals time-variable gravity field behaviour reflecting the structure underlying the Martian crustal dichotomy and indicates the preservation of a 200–400 °C thermal anomaly in the present-day mantle below the southern highlands.

## Main

Mars exhibits a prominent dichotomy in crustal structure. Whereas low-lying plains cover the northern hemisphere of Mars, the southern hemisphere is more topographically elevated, rugged and densely cratered^16^. These asymmetries potentially accompany an approximately 25 km mean variation in crustal thickness^11^ (or a roughly 200 kg m^−3^ variation in crustal density^17,18^), as well as spatial differences in crustal magnetization^19^ and seismic wave attenuation^20^. The northern lowlands may also have held surface water before the loss of Mars’s putative oceans to space^21^. Understanding the origin of the crustal dichotomy of Mars therefore constrains the planet’s ancient hydrology and the timing of surface conditions capable of supporting life^22^.

The origin of the hemispheric crustal dichotomy of Mars is widely debated. Some studies suggest that a giant impact disrupted the primordial mantle of the planet and either thickened the crust across the southern hemisphere^23,24^ or excavated the northern hemisphere^25^. Other studies suggest that mantle convection over one hemisphere drives subsidence and resurfacing of the northern crust or thickening and uplift of the southern crust^12,13^. These formation hypotheses for the crustal dichotomy predict long-lived structures within the Martian deep interior that may persist into the present day^12,13,23,24^. For example, crustal thickening over the southern hemisphere could enhance thermal insulation and radiogenic heating of the deeper interior, resulting in a structurally weak underlying mantle^14,15^. Here we investigate the character of these potential heterogeneities at depth by analysing the gravitational response of Mars to seasonal (that is, 687 Earth days) tidal interactions with the Sun.

The Martian gravity field can be expressed in terms of spherical harmonic coefficients of degree *ℓ* and order *m* (*C*_*ℓm*_ and *S*_*ℓm*_), with the full wavelength at a given *ℓ* usually defined as roughly 2π*R*/*ℓ* (*R* = 3,396 km)^26^. Temporal variations can be modelled as cyclic perturbations to these coefficients and separated into in-phase (or ‘cosine’, *A*) and (a quarter cycle) out-of-phase (or ‘sine’, *B*) components over a given period (that is, $\Delta {C}_{{\ell }m}^{A,B}$ and $\Delta {S}_{{\ell }m}^{A,B}$) (Methods). For a spherically symmetric planet, forcing at a given degree and order excites deformation only at the same degree and order. In this case, tidal forcing acts almost entirely at degree-2 and will contribute negligibly to the gravity field of Mars at higher degrees (that is, $\Delta {C}_{3m}^{A,B},\Delta {S}_{3m}^{A,B}\approx 0$). However, lateral heterogeneity in the internal structure of Mars (for example, north–south variations in shear modulus) can couple with degree-2 forcing to produce substantial degree-3 signals such that $\Delta {C}_{3m}^{A,B},\Delta {S}_{3m}^{A,B}\ne 0$ (ref. ^8^). Moreover, although static gravity fields carry some sensitivity to deep-seated density structure (especially at long wavelengths), their sensitivity is weighted towards structure in the shallower interior at all degrees (equation (4) of ref. ^27^). By contrast, the low-order non-zonal (that is, *m* ≠ 0) time-variable gravity field of Mars is most sensitive to lateral variations in the shear modulus of the mantle, as these heterogeneities generate larger mass redistributions than comparable perturbations within shallower layers^4,9^. Measuring time-variable gravity signals (for example, by analysing the trajectory of orbiting spacecraft) correspondingly provides a direct means to constrain the extent of Mars’s deep-seated heterogeneities^4^.

## Measuring the seasonal tidal response of Mars

To recover the seasonal degree-3 tidal signals of Mars, we analyse Earth-based X-band Doppler tracking data acquired by the NASA Deep Space Network (DSN) from several Martian orbiters. We use radio science data from MGS, ODY and MRO spanning a total of 16 years. Our data processing procedure follows the approach used to determine the time-varying zonal (*m* = 0) gravity field of Mars in ref. ^28^ but is extended in this study to include non-zonal *ℓ* = 2 and *ℓ* = 3 time-varying gravity field coefficients over the seasonal (or annual) 687-day period.

**Extended Data Fig. 1:**
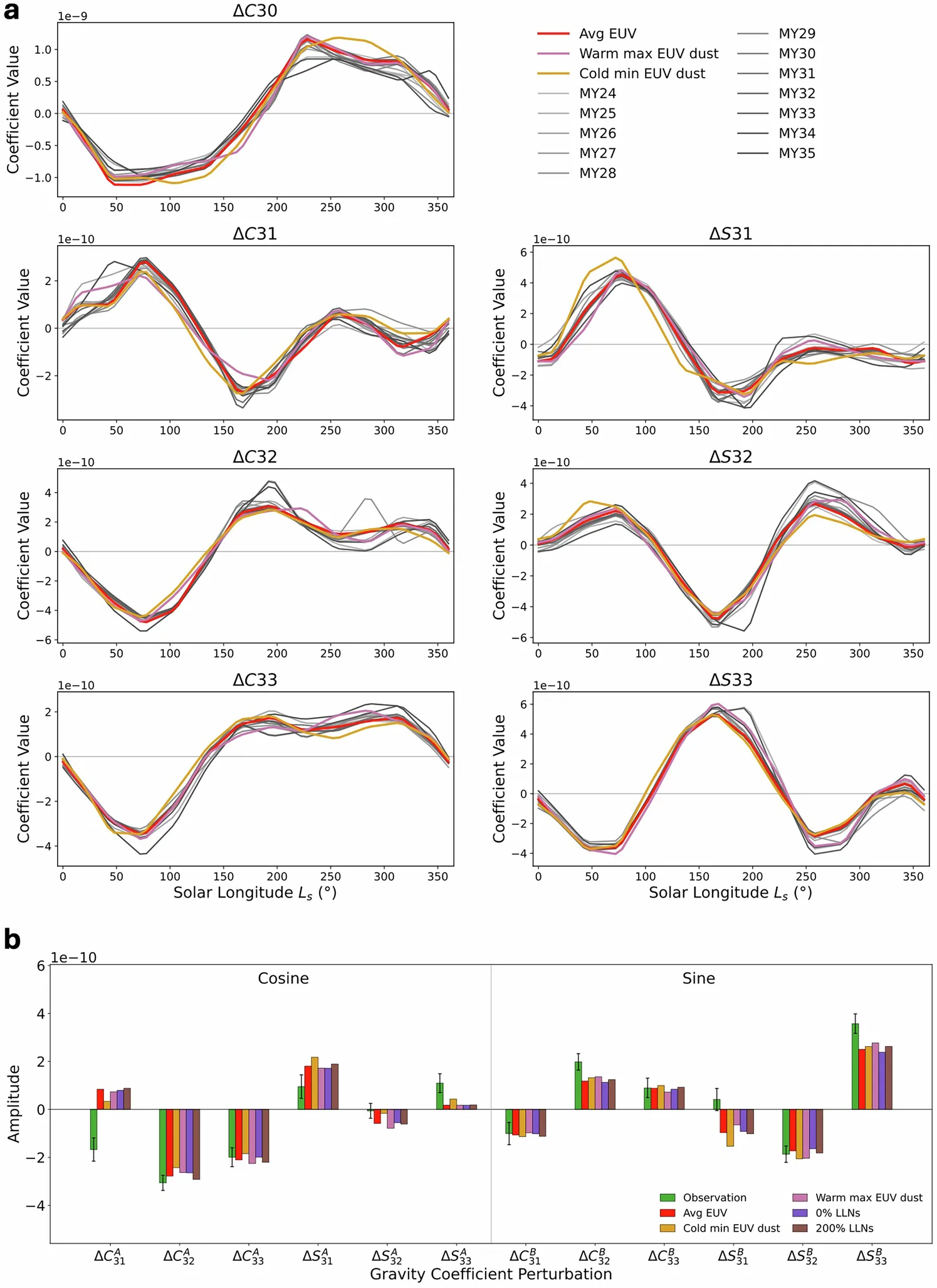
Seasonal evolution and atmospheric-model sensitivity of the degree-3 gravity signals. **a**, Seasonal evolution of degree-3 gravity coefficients across models for the atmosphere of Mars. Curves show the 4π-normalized, demeaned seasonal time series of Δ*C*_30_, Δ*C*_31_, Δ*S*_31_, Δ*C*_32_, Δ*S*_32_, Δ*C*_33_ and Δ*S*_33_ as a function of solar longitude *L*_s_. Coloured curves denote the average-EUV (red), warm/max-EUV with dust (pink) and cold/min-EUV with dust (orange) atmospheric scenarios, whereas grey curves show the individual realizations of the Avg EUV model from MY24 to MY35. Error bars in Fig. 1a are derived from inter-annual variations for the Avg EUV model. Horizontal grey lines mark zero amplitude in each panel. **b**, Sensitivity of the degree-3 gravity signals to atmospheric models and assumed loading Love numbers. Bars show the total observed seasonal degree-3 perturbations (green), the nominal atmospheric correction using the average-EUV scenario (red), atmospheric corrections for the cold/minimum-EUV/dust and warm/maximum-EUV/dust endmember scenarios (orange and pink) and cases with 0% and 200% the loading Love numbers ${k}_{{\ell }}^{{\prime} }$ assigned to the nominal average-EUV atmosphere (purple and brown; see equation (3)). The left and right groups show the cosine and sine terms, respectively. Error bars on the observations denote 15× formal uncertainties.

**Extended Data Fig. 2:**
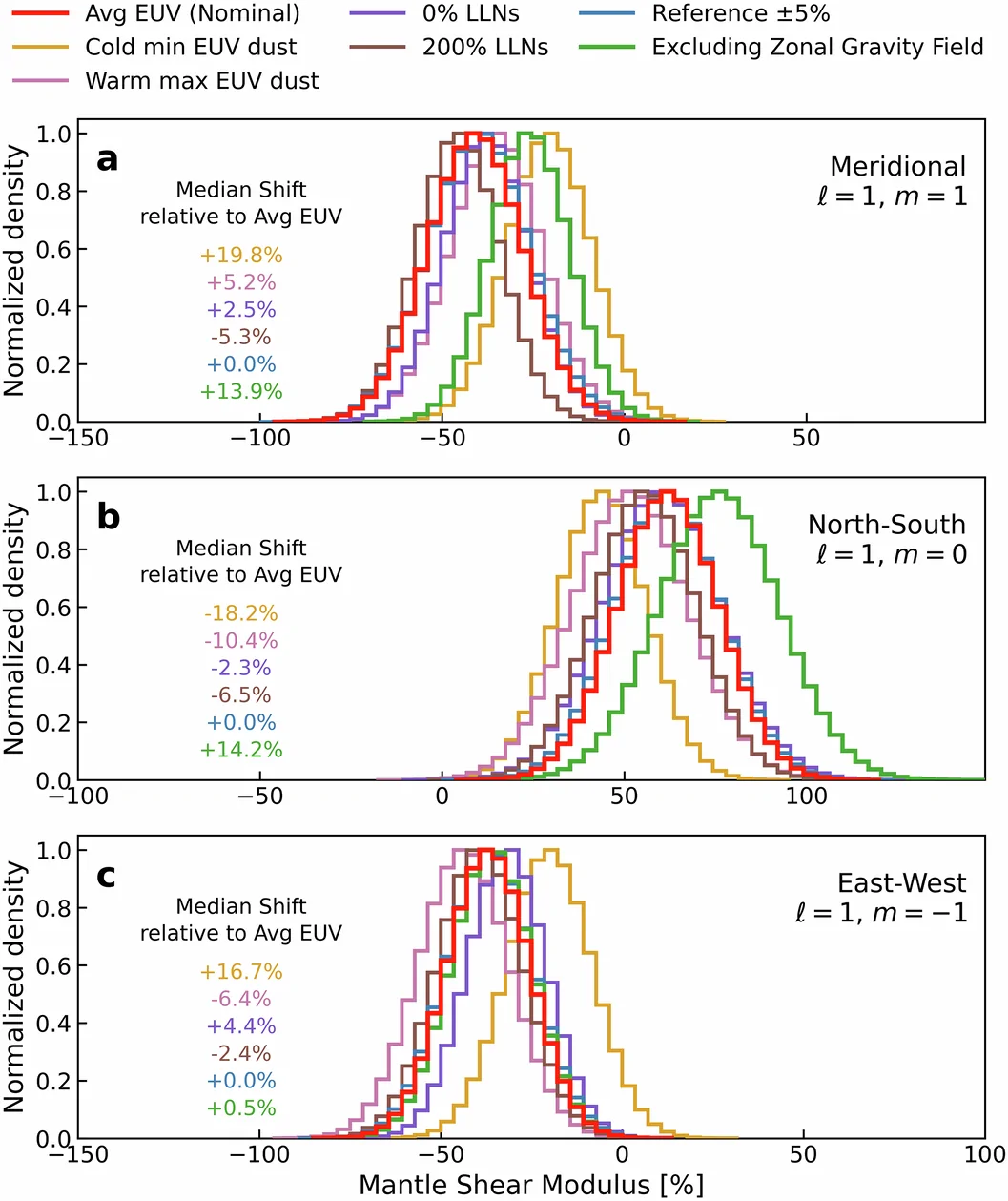
Sensitivity of tidal tomography results to atmospheric models, assumed loading Love numbers, the choice of reference 1D interior model and the inclusion of zonal gravity field terms. **a**–**c**, Posterior distributions of the degree-1 mantle shear modulus coefficients for the meridional *ℓ* = 1, *m* = 1 term (**a**), the north–south *ℓ* = 1, *m*= 0 term (**b**) and the east–west *ℓ* = 1, *m* = −1 term (**c**) for scenarios of the nominal atmospheric correction using the average-EUV scenario (red), atmospheric corrections for the cold/minimum-EUV/dust and warm/maximum-EUV/dust endmember scenarios (orange and pink), cases with 0% and 200% the loading Love numbers ${k}_{{\ell }}^{{\prime} }$ assigned to the nominal average-EUV atmosphere (purple and brown; see equation (3)), a case in which the reference shear and bulk moduli of the crust and mantle are sampled from a Gaussian distribution centred about nominal values (Extended Data Table 2) and a standard deviation equal to 5% of these values (light blue), and an inversion that excludes the zonal gravity harmonics in Extended Data Table 1 as constraints (green). Median shifts for each scenario relative to the average-EUV case is shown to the left in each plot.

Recovered non-zonal degree-3 gravity field coefficient perturbations are presented in Table 1 (for zonal and degree-2 coefficients, see Extended Data Table 1). To account for modelling uncertainties, particularly those arising from unmodelled non-gravitational accelerations, we inflate the formal uncertainties of these coefficients by a factor of 15 (see, for example, ref. ^4^) in Table 1 and in all subsequent analysis. As in ref. ^28^, we directly model the effects of the atmosphere by integrating the impact of the Mars general circulation model on gravity coefficients (Methods and Extended Data Figs. 1 and 2).

**Table 1:** Recovered degree-3 non-zonal time-varying gravity field for Mars with 15× formal uncertainties

Parameter	Amplitude	15× formal 1*σ* uncertainty
$\Delta {C}_{31}^{A}$	−2.5084 × 10^−10^	4.86 × 10^−11^
$\Delta {C}_{31}^{B}$	6.2830 × 10^−^^12^	4.65 × 10^−11^
$\Delta {C}_{32}^{A}$	−2.7967 × 10^−^^11^	3.17 × 10^−^^11^
$\Delta {C}_{32}^{B}$	7.9724 × 10^−^^11^	3.44 × 10^−^^11^
$\Delta {C}_{33}^{A}$	1.0453 × 10^−^^11^	3.95 × 10^−^^11^
$\Delta {C}_{33}^{B}$	1.6420 × 10^−^^12^	4.07 × 10^−^^11^
$\Delta {S}_{31}^{A}$	−8.5201 × 10^−^^11^	4.91 × 10^−^^11^
$\Delta {S}_{31}^{B}$	1.3759 × 10^−^^10^	4.62 × 10^−^^11^
$\Delta {S}_{32}^{A}$	5.2115 × 10^−^^11^	3.12 × 10^−^^11^
$\Delta {S}_{32}^{B}$	−1.3930 × 10^−^^11^	3.41 × 10^−^^11^
$\Delta {S}_{33}^{A}$	9.1466 × 10^−^^11^	3.95 × 10^−^^11^
$\Delta {S}_{33}^{B}$	1.0699 × 10^−^^10^	4.07 × 10^−^^11^

**Fig. 1:**
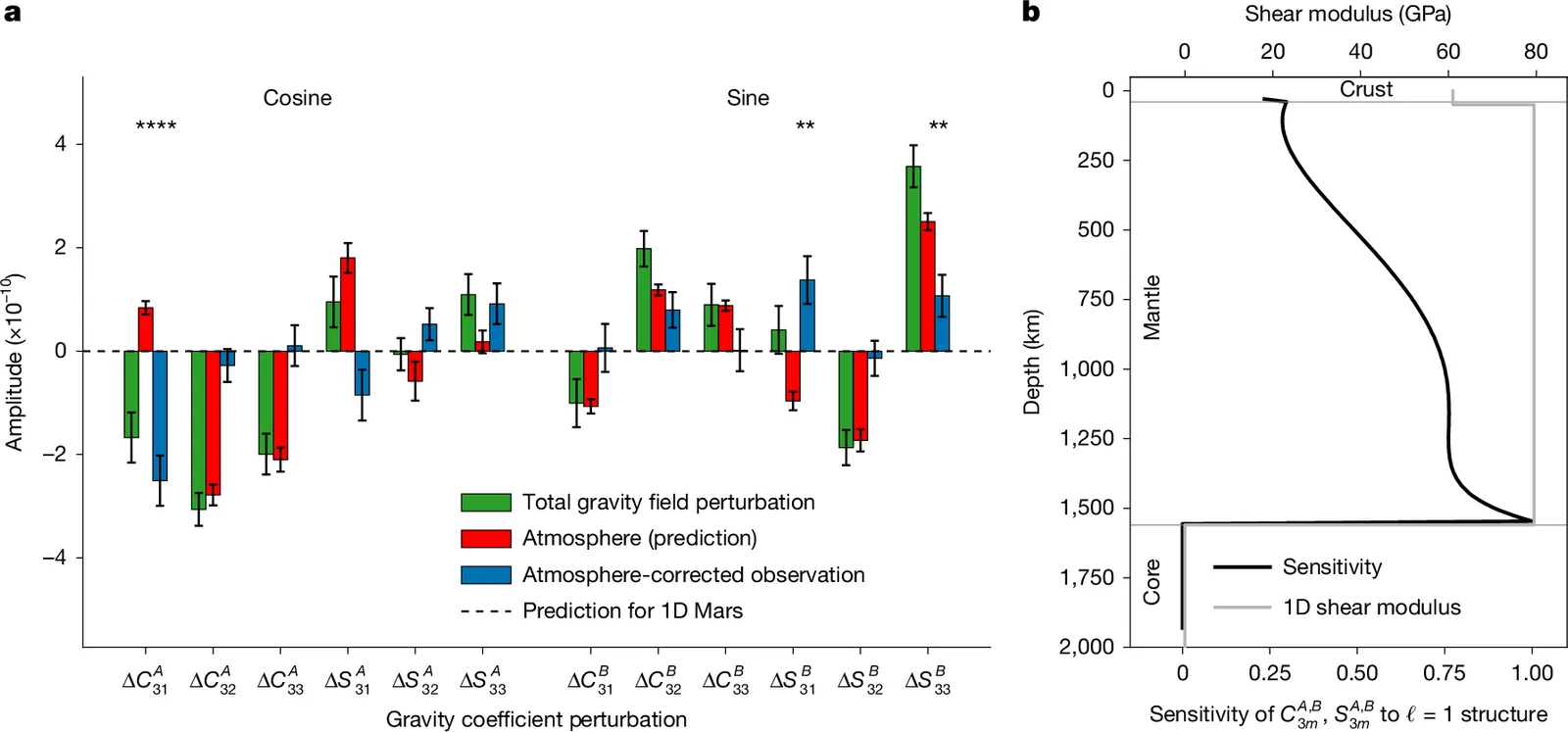
Sensitivity of the Martian seasonal gravity field to laterally heterogeneous structure. **a**, Bar chart showing the amplitude of seasonal temporal variations in selected Martian *ℓ* = 3 gravity field coefficients (that is, $\Delta {C}_{3m}^{A,B}$ and $\Delta {S}_{3m}^{A,B}$, in which *A* and *B* denote cosine and sine terms, respectively). Green, red and blue bars, respectively, denote the median total gravity field perturbations, the predicted median impact of the atmosphere on perturbations and the median observed gravity field perturbations corrected for the atmosphere. Error bounds on the green and blue bars represent 15× formal 1*σ* uncertainties and asterisks denote statistical significance of the atmosphere-corrected observation relative to the null hypothesis (that is, expectations for a spherically symmetric Mars with no atmosphere; amplitude = 0, the horizontal dashed line). **P < 0.01 and ****P < 0.0001 for two-tailed *t*-tests. Error bars on predictions for red bars are 1*σ* uncertainties computed by comparing inter-annual variations in the assumed atmospheric model (Extended Data Fig. 1). Error bars on atmosphere-corrected coefficients (blue bars) are generally much larger than (and therefore implicitly account for) uncertainties associated with inter-year variations in the Martian gravity field from our nominal atmospheric model (red bars) but do not directly consider the impact of extreme atmospheric models on coefficients (Methods and Extended Data Fig. 1). **b**, Normalized sensitivity of gravity coefficients to *ℓ* = 1 perturbations in shear modulus versus depth for Martian interiors subject to *ℓ *= 2 tidal forcing. The average shear modulus with depth assumed for Mars is plotted as a grey line for reference (Extended Data Table 2). Labels refer to vertical regions spanning the crust (0–50 km depth), the mantle (50–1,560 km) and the core (1,560–3,390 km).

Coefficient amplitudes are 4π-normalized and evaluated over the seasonal, 687-day period referenced to the J2000 epoch. Superscripts *A* and *B* denote in-phase (cosine) and out-of-phase (sine) signal components, respectively. Presented values correspond with the solid-body response (that is, blue bars) shown in Fig. 1a. Reported 15× formal 1*σ* bounds are larger than (and therefore implicitly account for) uncertainties associated with inter-year variations in the Martian gravity field from our nominal atmospheric model (Fig. 1a) but do not directly consider the impact of extreme atmospheric models on coefficients (for example, very warm, cold or high-dust conditions; Methods and Extended Data Fig. 1).

## Modelling the mantle structure of Mars

We recover non-zonal *ℓ* = 3 perturbations to the Martian gravity field that deviate substantially from values predicted for a spherically symmetric Mars with an atmosphere (Fig. 1a). For example, the cosine coefficient of the *ℓ* = 3, *m* = 1 gravity field perturbation (that is, $\Delta {C}_{31}^{A}$) deviates approximately 300% from expectations for a spherically symmetric Mars with atmospheric loading (with >99.99% confidence, the difference between the green and red bars in Fig. 1a), suggesting coupling between tidal deformation and laterally heterogeneous internal structure in the mantle of Mars (Fig. 1b). To constrain the nature of these asymmetries, we perform a Bayesian, Markov chain Monte Carlo inversion (Methods). Inversions incorporate both degree-2 and degree-3 (zonal and non-zonal) time-variable gravity fields of Mars as constraints (Table 1, Extended Data Table 1 and Methods).

Our model space consists of lateral shear modulus variations imposed onto a 1D reference interior constrained by Mars’s degree-2 tidal Love number, moment of inertia and seismic travel-time data^29^ (Extended Data Table 2). We expand these variations in a spherical harmonic basis truncated at degree *ℓ* = 3 across two internal layers: the crust (0–50 km depth) and the mantle (50–1,560 km depth). Lateral crustal thickness and density variations on Mars negligibly (<0.3%) affect the time-variable gravity field and are therefore ignored for inversions. Heterogeneities in the core (including a potential solid inner layer^30^) also minimally affect time-variable gravity fields (Fig. 1b). We consequently prescribe a uniform elastic structure below 1,560 km depth. We use the semi-analytical spectral code LOV3D (ref. ^10^) to forward compute coupling between degree-2 forcing and lateral heterogeneity for candidate interior structures (Methods). Note that the zonal gravity field variations of Mars are also sensitive to seasonal CO_2_ condensation over the polar caps^31^. We accordingly model *m* = 0 terms as the sum of the solid-body tide and a seasonal mass exchange of 6.2 × 10^15^ kg and 8.4 × 10^15^ kg over the northern and southern polar caps, respectively^28^ (Methods).

**Extended Data Fig. 3:**
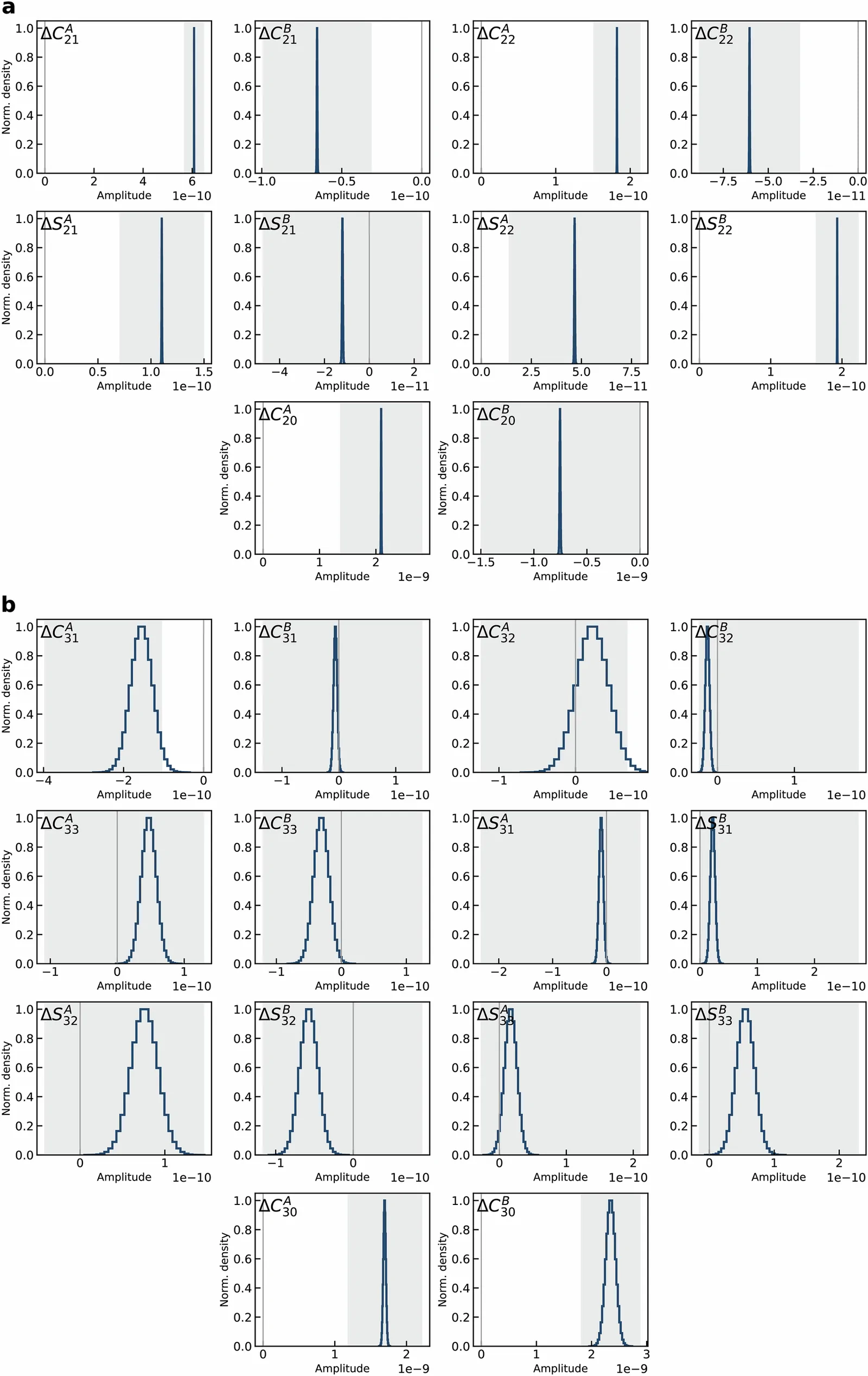
Posterior distributions of annual gravity coefficient variations. **a**,**b**, Panels show normalized histograms for the posteriors of degree-2 (**a**) and degree-3 (**b**) annual variations in gravity coefficients ($\Delta {C}_{{\ell }m}^{A,B}$ and $\Delta {S}_{{\ell }m}^{A,B}$) obtained from tidal tomography inversions. Grey-shaded regions denote 45× the formal uncertainty derived from gravity field inversions (that is, 3× the 15× 1*σ* uncertainties in Table 1 and Extended Data Table 1).

**Extended Data Fig. 4:**
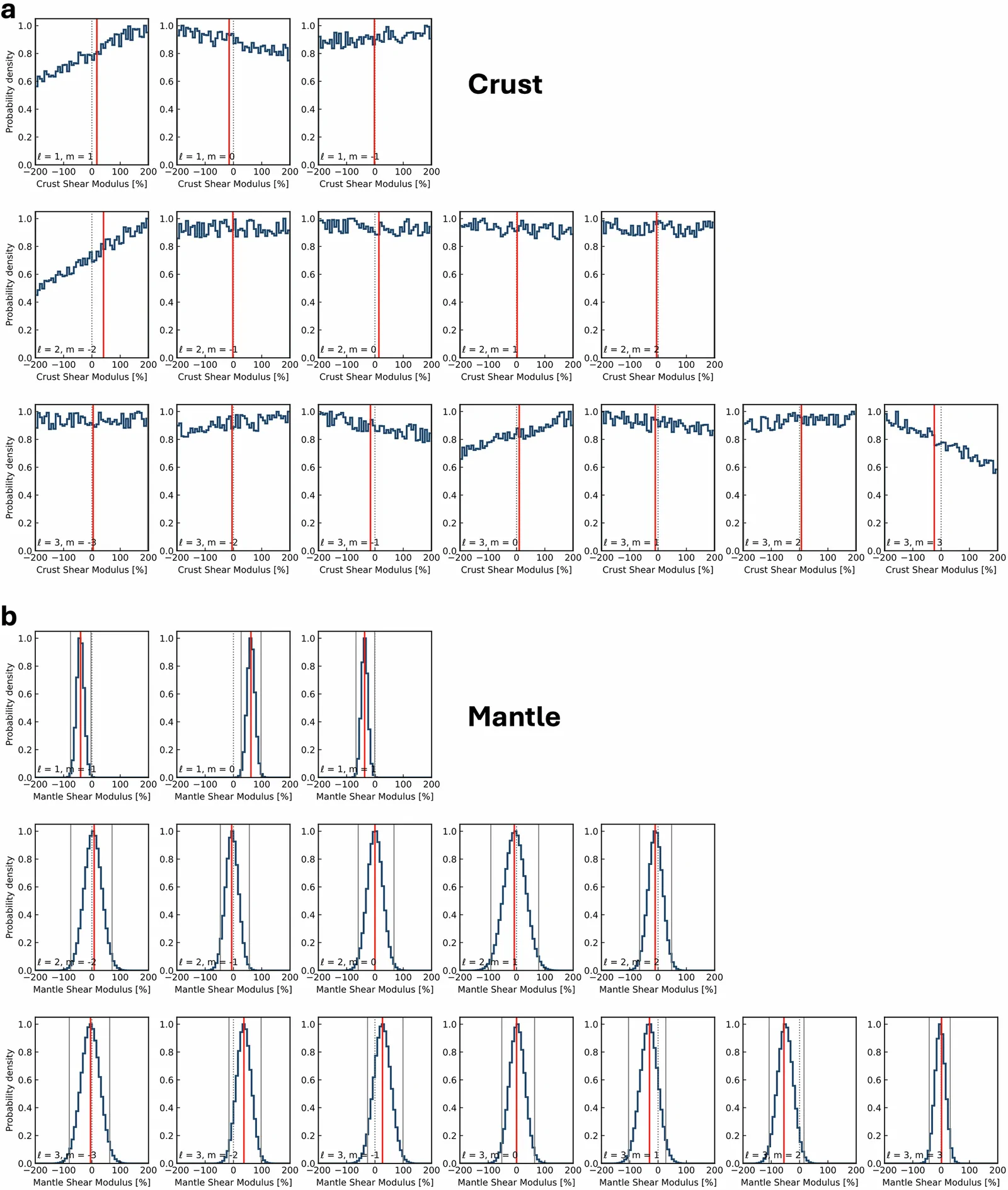
Posterior distributions of perturbations in the shear modulus of the crust and mantle of Mars. **a**, Crust. **b**, Mantle. In each panel, top row: degree-1 (*ℓ* = 1, *m* = 1, 0, −1); middle row: degree-2 (*ℓ* = 2, *m* = −2, −1, 0, 1,2); bottom row: degree-3 (*ℓ* = 3, *m* = −3, −2,− 1, 0, 1, 2, 3). Vertical lines mark *x* = 0 (dotted), the 0.3rd and 99.7th percentiles (solid grey) and the 50th percentile (solid red).

Our results indicate that degree-1 (*ℓ* = 1) shear modulus structure, which reflects a hemispheric pattern, enhances the amplitude of *ℓ* = 3 coefficients for Martian interiors subject to seasonal tidal forcing at *ℓ* = 2 (Fig. 1b). For example, the *ℓ* = 2, *m* = 1 harmonic associated with the obliquity tide of Mars interacts with north–south (that is, *ℓ* = 1, *m* = 0) shear modulus structure (lower/higher shear modulus values in the mid-latitudes of the southern/northern portions of the meridional hemisphere that spans −90° E to 90° E longitude) to regionally increase/decrease outward radial deformation. The resulting mass displacement yields an *ℓ* = 3, *m* = 1 gravity signature that enhances the amplitude of $\Delta {C}_{31}^{A}$. Interaction between other hemispherical variations such as east–west (*ℓ* = 1, *m* = −1) and meridional (*ℓ* = 1, *m* = 1) asymmetries and the full degree-2 Martian tide (that is, components arising from both obliquity and eccentricity of Mars) is more complex^10^ but produces deviation of several degree-3 gravity coefficients from zero (Extended Data Fig. 3). By contrast, the degree-2 time-variable gravity field does not measurably deviate from predictions for a spherically symmetric interior (Extended Data Fig. 3), suggesting minimal internal heterogeneity that strongly couples with *ℓ* = 2 forcing^10^ (for example, *ℓ* = 2 patterns; Extended Data Fig. 4).

**Fig. 2:**
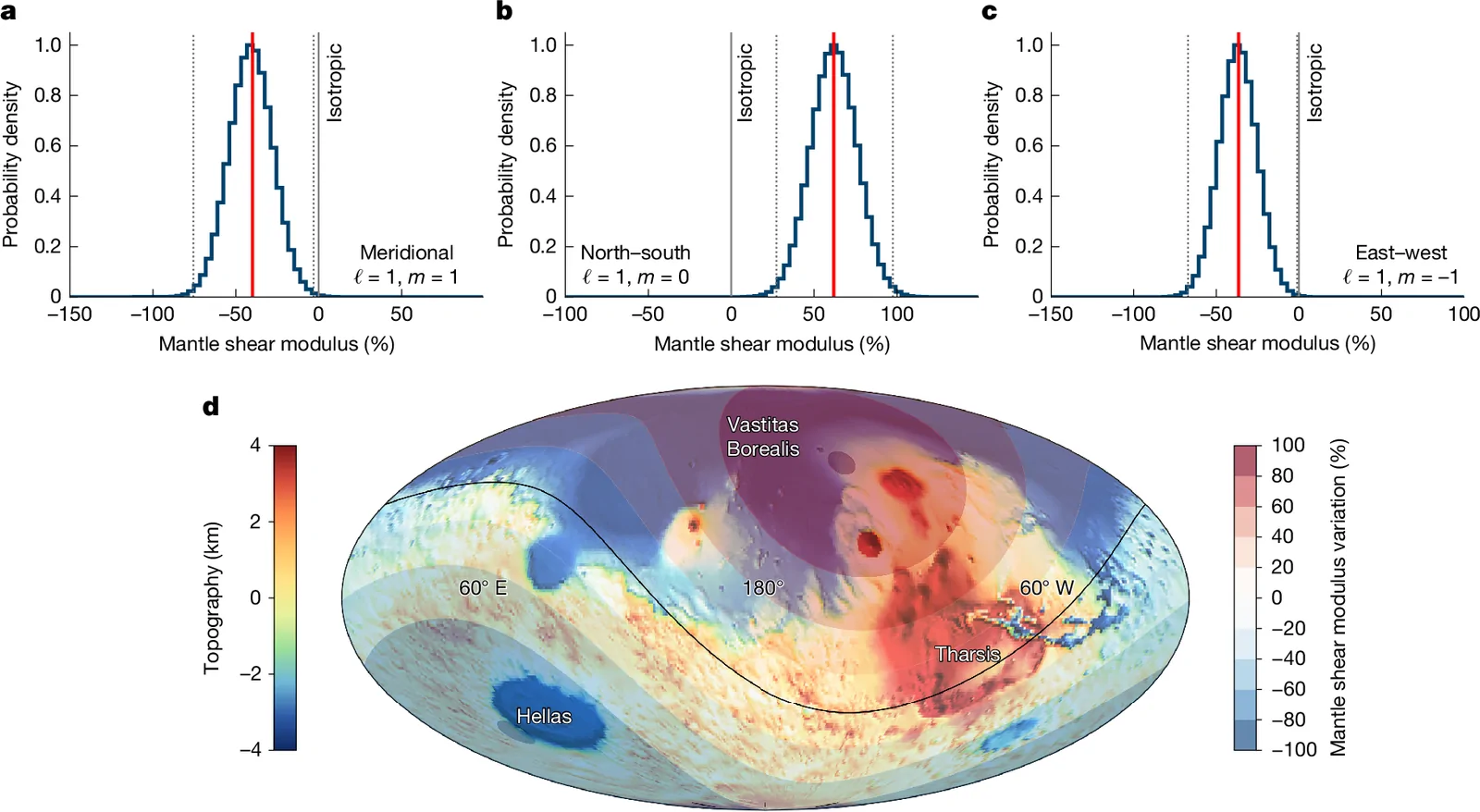
Tidal tomography of the Martian mantle. **a**–**c**, Histograms of inverted coefficient values that describe internal hemispheric (degree-1) variations in shear modulus (in per cent relative to the bulk value) for the Martian mantle (50–1,560 km depth). Meridional (**a**), north–south (**b**) and east–west (**c**) labels denote order-1, order-0 and order-1 variations, respectively. Dotted lines indicate 0.3rd and 99.7th percentiles (that is, 3*σ* confidence bounds) and the solid red lines indicate maximum a posteriori solutions (that is, the location of the absolute maximum of posterior distributions). A full list of derived harmonic coefficients describing 3D structure is shown in Extended Data Table 3. **d**, Plot of Martian topography (MGS-M-MOLA-5-IEGDR (ref. ^54^), red–orange–yellow–green–blue colour map) overlain by semi-transparent map of the combination of maximum a posteriori solutions for degree-1 variations in mantle shear modulus shown in **a**–**c** (red–blue colour map). Black contour indicates the trace of the zero value of shear modulus variations. Approximate locations of Tharsis, Vastitas Borealis and Hellas basin are indicated. Map is shown in Mollweide projection.

**Extended Data Fig. 5:**
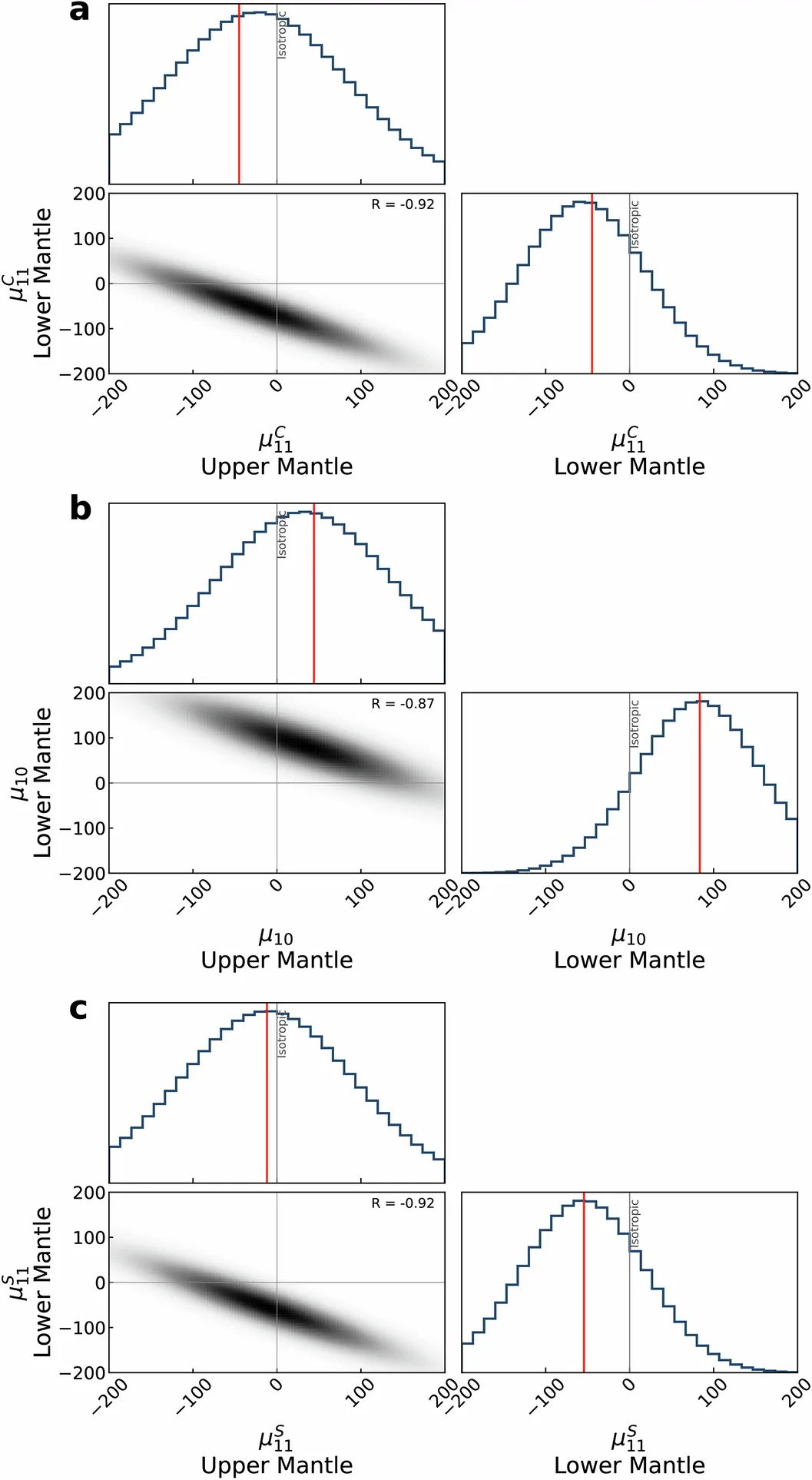
Corner plots for degree-1 shear modulus perturbations in an inversion with the mantle subdivided into several layers. **a**–**c**, Degree-1 coefficients ${\mu }_{11}^{C}$ (**a**), *μ*_10_ (**b**) and ${\mu }_{11}^{S}$ (**c**) are derived for an inversion that subdivides the Martian mantle into a lower portion (radius 1,830–2,520 km) and an upper portion (radius 2,520–3,340 km). Pearson correlation coefficients *R* are shown alongside 2D posterior distributions (values close to −1 indicate a substantially non-unique trade-off between recovered heterogeneity amplitudes). Red lines on 1D histograms indicate the median (50th percentile) of distributions. Vertical grey lines in 1D histograms indicate values expected for a laterally isotropic reference interior.

Our inversions predict statistically significant degree-1 variations in the shear modulus of the Martian mantle. Specifically, we resolve north–south, meridional and east–west patterns with peak-to-peak amplitudes (and 3*σ* uncertainties) of 60 ± 40%, 42 ± 38% and 36 ± 34%, respectively (Fig. 2a–c), as well as an overall variation of 81 ± 60% (or >20%). Inversions fit all time-variable gravity field constraints to within 3*σ* uncertainty (Extended Data Fig. 3) and do not recover any statistically significant lateral variations in shear modulus for the crust of Mars (Extended Data Table 3 and Extended Data Fig. 4a) or for degrees higher than *ℓ* = 1 in the mantle (Extended Data Table 3 and Extended Data Fig. 4b). Note that heterogeneity amplitudes represent averages over the entire mantle depth range. This averaging is necessary to produce statistically significant results, because the sensitivity of degree-3 time-variable gravity to 3D structure varies gradually with depth (Fig. 1b), resulting in substantial non-uniqueness between recovered heterogeneity amplitudes when the mantle is subdivided into several layers (Extended Data Fig. 5). Even so, our inversions do not necessarily preclude more vertically localized structure within the mantle of Mars (for example, at the base of the lithosphere^14^ or near the core–mantle boundary^32^).

The hemispheric mantle shear modulus variation of Mars broadly matches the spatial pattern of the crustal dichotomy (Fig. 2d). For example, combining maximum likelihood solutions for recovered degree-1 order-1, order-0 and order-−1 variations (Fig. 2a–c) results in a region of increased shear modulus beneath the northern lowlands (centred at 45° N, 138° W over Vastitas Borealis; Fig. 2d) and a corresponding reduction in shear modulus beneath the southern highlands (centred at 45° S, 42° E near Hellas basin; Fig. 2d). The zero-value contour of the mantle shear modulus variation of Mars also tracks the boundary of the crustal dichotomy, including its southward (or northward) deflection over the anti-meridional (or meridional) hemisphere. The correspondence between surface geology and mantle stiffness variations weakens between 40° W and 139° W near the volcanic Tharsis rise region, potentially because of overprinting of the dichotomy boundary by this structure^16^. The lack of anomalies observed directly beneath Tharsis (Fig. 2d) also suggests that present-day internal structures associated with this feature are small in amplitude (that is, compared with asymmetries in Fig. 2), shallow or restricted to a relatively small (*ℓ* > 3) lateral region within the interior^33^.

## Thermal anomaly beneath the highlands of Mars

**Fig. 3:**
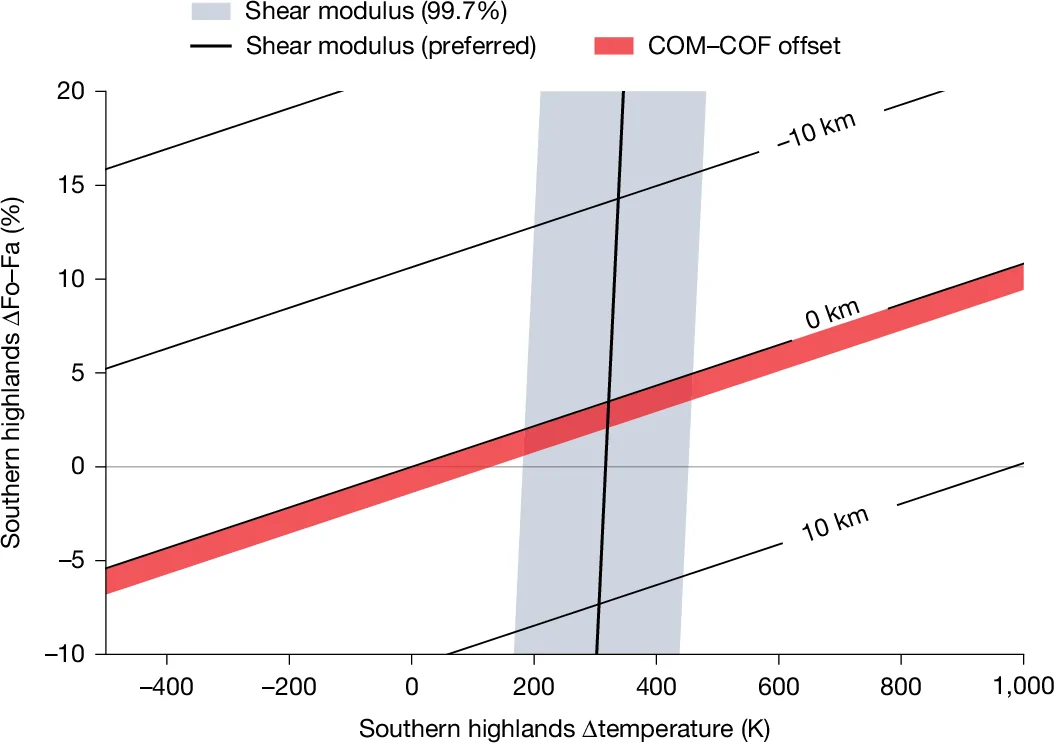
Relationship between temperature, composition and inferred shear modulus variations in the Martian mantle. Shaded regions indicate the constrained ranges of the inferred Martian effective hemispheric shear modulus variation and the impact of mantle structure on the COM–COF offset as a function of southern highlands mantle temperature anomaly and fayalite–forsterite mole fraction contrast (Fa–Fo) (for details on calculations, see Methods). Values are projected onto a degree-1 pattern with poles centred at 45° N, 138° W (northern lowlands) and 45° S, 42° E (southern highlands). Black contours indicate COM–COF offsets at 10-km intervals. Because the COM–COF offset is sensitive to the structure of both the crust and the mantle, our modelled 0–1.31-km range of values accounts for the possibility of: (1) an anomalous COF that is fully attributable to elevated topography over the southern highlands (paired with Airy or Pratt compensation of this structure at depth) or an anomalous COM arising from a relatively dense crust over the northern lowlands (that is, the 0 km bound)^11,18,35^; (2) an anomalous COM that is only attributable to mantle density variations (that is, the 1.31 km bound); and (3) intermediate cases. The grey-shaded area and thick solid black line, respectively, denote the 99.7% confidence bounds and preferred value for the overall shear modulus difference of 81 ± 60% inferred from gravity data in this work (Fig. 2). By identifying overlapping portions of parameter space that satisfy both the 99.7% range of inferred shear modulus variation and COM–COF offset, we infer that hemispheric asymmetries can be explained by a temperature anomaly of 200–400 ºC and an iron enrichment of up to 5% in the southern highlands mantle.

**Extended Data Fig. 6:**
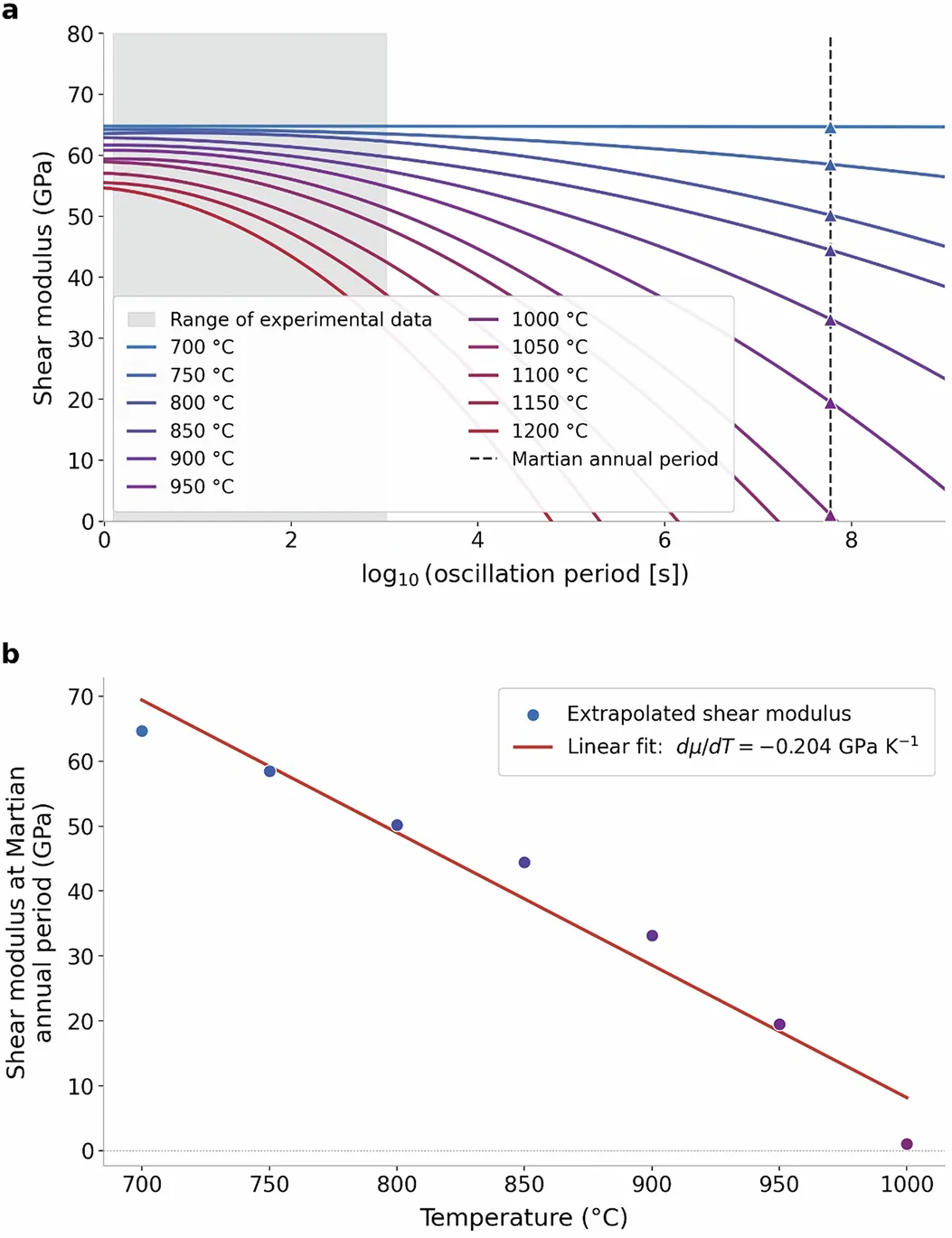
Sensitivity of olivine shear modulus to temperature at the Martian annual period. **a**, Shear modulus versus oscillation period for the background-only Burgers model of olivine rheology evaluated from experiments on crystals at temperatures between 700 and 1,200 °C (see Fig. 1a of ref. ^34^). Coloured curves are power-law fits extended beyond the range of experimental data (grey shading) to the Martian annual period (5.94 × 10^7^ s; dashed vertical line). **b**, Shear moduli evaluated at the Martian annual period as a function of temperature. A linear trendline (red) yields d*μ*/d*T* ≈ −0.204 GPa K^−1^, which is used to recover hemispheric variations in temperature from shear modulus asymmetries (Fig. 3).

Our modelling suggests that the anomalous degree-3 gravity field of Mars arises owing to a substantial, >20% lateral variation in the mantle’s effective shear modulus at the seasonal timescale. What could generate such a large contrast? At a given pressure, the rigidity of mantle rock depends on both temperature and composition. At seismic timescales, crystalline temperature differences would need to exceed an unrealistically large >1,000 K to produce observed shear modulus variations^4^. However, when extended to the Martian annual period (Methods and Extended Data Fig. 6), the effective shear modulus of olivine becomes approximately 15–20 times more sensitive to temperature^34^. As such, recovered shear modulus variations can mostly be explained by a hemispheric temperature anomaly of approximately 200–400 ºC in the present-day Martian mantle (Fig. 3 and Methods). Thermal modelling also suggests that the insulating effect of the thicker crust in the southern highlands can result in a hemispheric temperature contrast >200 ºC (Fig. 3f of ref. ^15^), consistent with our results. The centre-of-mass–centre-of-figure (COM–COF) offset of Mars also limits the potential compositional component (for example, iron, water content) of asymmetries because, in isolation, such differences (and their associated density structure) would need to produce an offset that is approximately 50 times larger than observations^35^ to account for the inferred shear modulus variations. Even so, our models allow for up to 5% iron enrichment (that is, variation in forsterite–fayalite mole fraction or ΔFo–Fa) within the mantle of the southern highlands (Fig. 3).

**Fig. 4:**
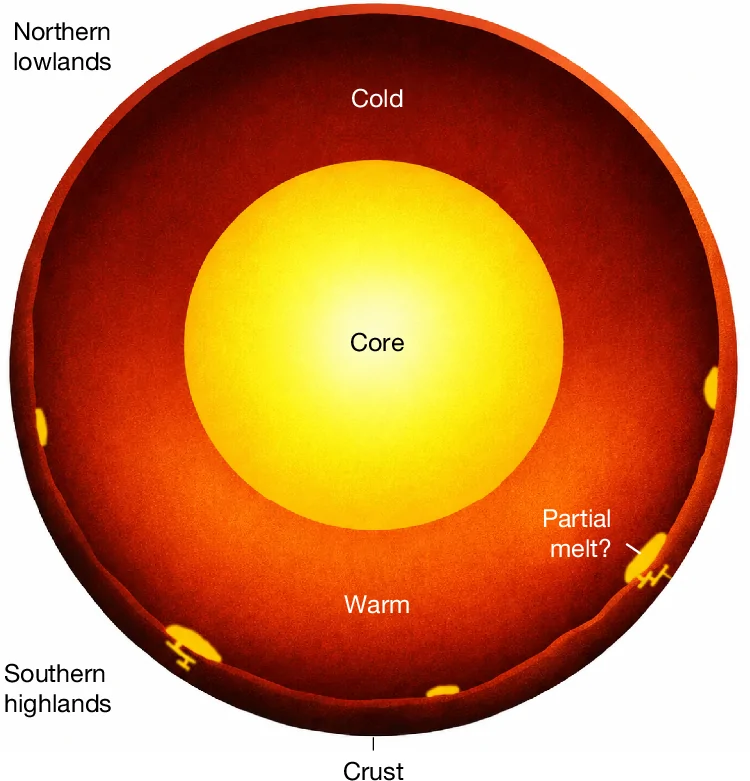
Conceptual model of the interior structure of Mars as inferred in the present study. Measured temporal variations in degree-3 gravity indicate a thermal anomaly within the mantle beneath the southern highlands (light yellow to orange regions outside the core). This anomaly may promote partial melting below the lithosphere of Mars, which ascends and stalls in the crust before reaching the surface^14^. This upwelling could result in thickening and enhanced magnetization across the southern highlands crust of Mars. By contrast, the mantle beneath the northern lowlands is comparatively cool (dark red to brown regions). Illustration is not to scale.

Mantle heterogeneities may preserve signatures of, and thus provide independent constraints on, geodynamic processes that influence the evolution of the crustal dichotomy. We consider three such candidate processes: a giant impact, spontaneous degree-1 upwelling (that persists into the present day) and enhanced thermal insulation or radiogenic heating of the southern highlands mantle by a thick overlying crust. Spontaneous degree-1 upwelling or thermal insulation would produce a mainly thermal mantle anomaly^12–15^ as well as potential secondary compositional signatures through decompression melting below the lithosphere^12–15^. However, enhanced melt extraction in these scenarios would deplete the southern highlands mantle in iron^12,36^, opposite to the observations (Fig. 3). By contrast, a giant impact is (in isolation) not expected to result in a long-term secular variation in temperature of several hundred kelvin over a given hemisphere^37^. A hybrid scenario could resolve this mismatch. For example, following the formation of the northern lowland crust from the Borealis impact, the northern hemisphere mantle is first depleted in iron^36^, leaving the southern mantle relatively enriched. The resultant thicker southern crust could also trigger preferential thermal insulation^15^ or degree-1 upwelling^38^ below the highlands of Mars (the latter would be favoured absent variations in crustal thickness or radiogenic material abundance^18^), increasing the present-day temperature of this region (Figs. 3 and 4).

Temperature variations may also influence the dissipative properties of the mantle of Mars. For example, comparisons of low-frequency marsquakes detected by the InSight (Interior Exploration using Seismic Investigations, Geodesy and Heat Transport) lander indicate substantially lower quality factors (*Q* ≈ 500) for events originating in the nearby Terra Cimmeria region of the southern highlands than for events from Cerberus Fossae in the northern lowlands (*Q* ≈ 800–2,000)^20^. These differences in attenuation can be attributed to lateral variations in the temperature of the mantle of a few hundred kelvin (Fig. 4 of ref. ^20^). A warm southern highlands mantle might also result in localized melting^14^, which could reduce the 200–400 ºC temperature anomaly required to explain gravity field observations (Fig. 3). However, the *Q* values inferred for the southern highlands remain well above those expected for partially molten olivine (*Q* ≈ 100 or lower)^20^. Even so, future electromagnetic sounding measurements (for example, ref. ^39^) could resolve whether the inferred thermal anomaly in the southern highlands mantle is associated with isolated magma pockets or with a more continuous molten layer at depth (for example, refs. ^14,32^).

The preferential magnetization of the southern highlands crust^19^ may also reflect the effects of a laterally heterogeneous interior. For example, upwelling beneath the southern highlands may have heated the overlying crust above Curie temperatures while Mars’s ancient dynamo was active (4.5–4.1 billion years ago)^40^. These temperatures may have subsequently fallen below Curie temperatures before the cessation of the dynamo, allowing affected portions of the southern highlands to acquire their present-day remanent magnetization^13,41^. Alternatively, increased heat flow at the core–mantle boundary (and in the overlying mantle) may have regionally enhanced convection in the outer core of Mars, resulting in a stronger dynamo below the southern highlands^42^. Inferred asymmetries in mantle structure may therefore constitute a present-day remnant of geodynamic processes associated with the generation of Mars’s early dynamo (Fig. 4).

## Volcanism and future measurements

Although melt is not required by our results (Fig. 3), some thermal models predict that higher temperatures in the southern highlands mantle would increase magma production in this region^14^. Such pockets of deep-seated melt are expected to ascend and erupt onto the surface over timescales of several Myr (ref. ^43^) (Methods). Yet recent (tens of Myr) volcanic activity is confined to Cerberus Fossae in the north^44^. We propose two potential solutions to this apparent paradox. The first is that the thicker (and potentially lower-density^45^) crust in the southern highlands may present a barrier to melt ascent. Under this scenario, melt would stall in the mid-crust and generate local intrusive features (Fig. 4). These intrusions would be difficult to detect in surface images but may be resolved from very-high-resolution (*ℓ* > 300) measurements of the static gravity field of Mars^46^. The second solution is that melt ascent and eruption requires a background extensional stress field^47^. Tectonic features in the southern highlands are dominantly compressional (for example, ref. ^48^), as expected from the slow cooling of Mars. By contrast, Cerberus Fossae is a region undergoing localized extension^49^. In any case, the petrology of volcanic rocks sampled from different locations over the crust of Mars could constrain the spatial differences in mantle potential temperature implied by our results^50^.

Tidal tomography—or the inference of lateral variations in interior structure from a body’s time-variable response to tidal forcing—has been used to investigate the mantle of Earth^2^ and, more recently, the Moon^4^. Here we use nearly two decades of precise spacecraft tracking data to extend this approach to Mars. Future missions with dedicated gravity experiments (similar to GRACE for Earth^51^ or GRAIL for the Moon^52^) could resolve similar (or finer) variations in Martian mantle structure over much shorter mission durations^53^. In the future, these techniques may also be applied to other planetary bodies that exhibit pronounced low-order asymmetries in structure, including Ganymede, Mercury, Io and Enceladus. Because tidal tomography largely relies on remote measurements, it represents a powerful tool for future missions seeking to characterize the interiors of planetary bodies without landed spacecraft.

## Methods

### Modelling the time-varying gravity field

The gravitational potential for Mars, *U*(*r*, *λ*, *ϕ*), can be expressed as a sum over 4π-normalized spherical harmonic coefficients *C*_*ℓm*_ and *S*_*ℓm*_:

1 $$U(r,\lambda ,\phi )=\frac{{GM}}{r}\left[1+\mathop{\sum }\limits_{{\ell }=2}^{{\rm{\infty }}}{\left(\frac{{R}_{m}}{r}\right)}^{{\ell }}\mathop{\sum }\limits_{m=0}^{{\ell }}({C}_{{\ell }m}\cos m\lambda +{S}_{{\ell }m}\sin m\lambda ){P}_{{\ell }m}(\sin \phi )\right],$$

in which *G* is the gravitational constant, *M* is the mass of Mars, *R*_*m*_ is a reference radius, *P*_*ℓm*_ are fully normalized associated Legendre functions, *r* is the radial distance from the centre of the planet, *λ* is the planetocentric longitude and *ϕ* is the planetocentric latitude. The gravity field is expressed in the body-fixed frame of Mars (as defined in ref. ^26^), centred at the planet’s COM, so that the degree-1 coefficients vanish by definition. To describe the temporal variability of the Martian gravity field, periodic terms are introduced into the normalized coefficients. This formulation, implemented within the GEODYN II orbit determination framework^55^, allows the harmonic coefficients *C*_*ℓm*_ and *S*_*ℓm*_ to vary in time to account for periodic mass redistribution. This time-dependent expansion is written as:

2 $$\begin{array}{c}{C}_{{\ell }m}(t)={\bar{C}}_{{\ell }m}+\mathop{\sum }\limits_{k=1}^{3}\left[\Delta {C}_{{\ell }m}^{A,(k)}\cos ({\omega }^{(k)}t)+\Delta {C}_{{\ell }m}^{B,(k)}\sin ({\omega }^{(k)}t)\right],\\ {S}_{{\ell }m}(t)={\bar{S}}_{{\ell }m}+\mathop{\sum }\limits_{k=1}^{3}\left[\Delta {S}_{{\ell }m}^{A,(k)}\cos ({\omega }^{(k)}t)+\Delta {S}_{{\ell }m}^{B,(k)}\sin ({\omega }^{(k)}t)\right],\end{array}$$

in which ${\bar{C}}_{{\ell }m}\,{\rm{and}}\,{\bar{S}}_{{\ell }m}$ are static (that is, not changing with time) components of the Martian gravity field, *ω*^(*k*)^ represents the angular frequency of the *k*th periodic component in which *k* = 1, 2, 3 respectively correspond to the Martian annual (or seasonal), semiannual and triannual periods, and *t* is time following the reference epoch (J2000). The amplitudes $\Delta {C}_{{\ell }m}^{A,(k)}$, $\Delta {S}_{{\ell }m}^{A,(k)}$, $\Delta {C}_{{\ell }m}^{B,(k)}$ and $\Delta {S}_{{\ell }m}^{B,(k)}$ represent the cosine and sine coefficients of the *k*th harmonic component, which together describe the temporal modulation of each spherical harmonic term at the modelled frequencies. For this work, we specifically extract coefficients at the Martian annual period for use in tidal tomography inversions (that is, $\Delta {C}_{{\ell }m}^{A,B}\equiv \Delta {C}_{{\ell }m}^{A,B,(k=1)}$ and $\Delta {S}_{{\ell }m}^{A,B}\equiv \Delta {S}_{{\ell }m}^{A,B,(k=1)}$, *ω* ≡ *ω*_1_ = 1.058 × 10^−7^ rad s^−1^ and $t=\frac{\omega }{2{\rm{\pi }}}n+{t}_{0}$ (*n* is an integer) are yearly intervals after the J2000 epoch *t*_0_).

### Gravity field inversion

We co-estimate parameters $\Delta {C}_{{\ell }m}^{A,B}$ and $\Delta {S}_{{\ell }m}^{A,B}$ up to *ℓ* = 3 along with static coefficients ${\bar{C}}_{{\ell }m}$ and ${\bar{S}}_{{\ell }m}$ up to *ℓ* = 120 in equation (2) through our gravity inversion procedure. To do so, we reprocess Earth-based radiometric (X-band) Doppler tracking data acquired by the DSN from the MGS, ODY and MRO missions. The dataset spans approximately 16 years, corresponding to about one and a half solar cycles^28^. Following an initial step of correcting for non-gravitational effects on spacecraft acceleration, we minimize residuals between predicted spacecraft range rate values (that is, for an iteratively updated gravity field) and range rate observations. These steps are described in detail below.

**Extended Data Fig. 7:**
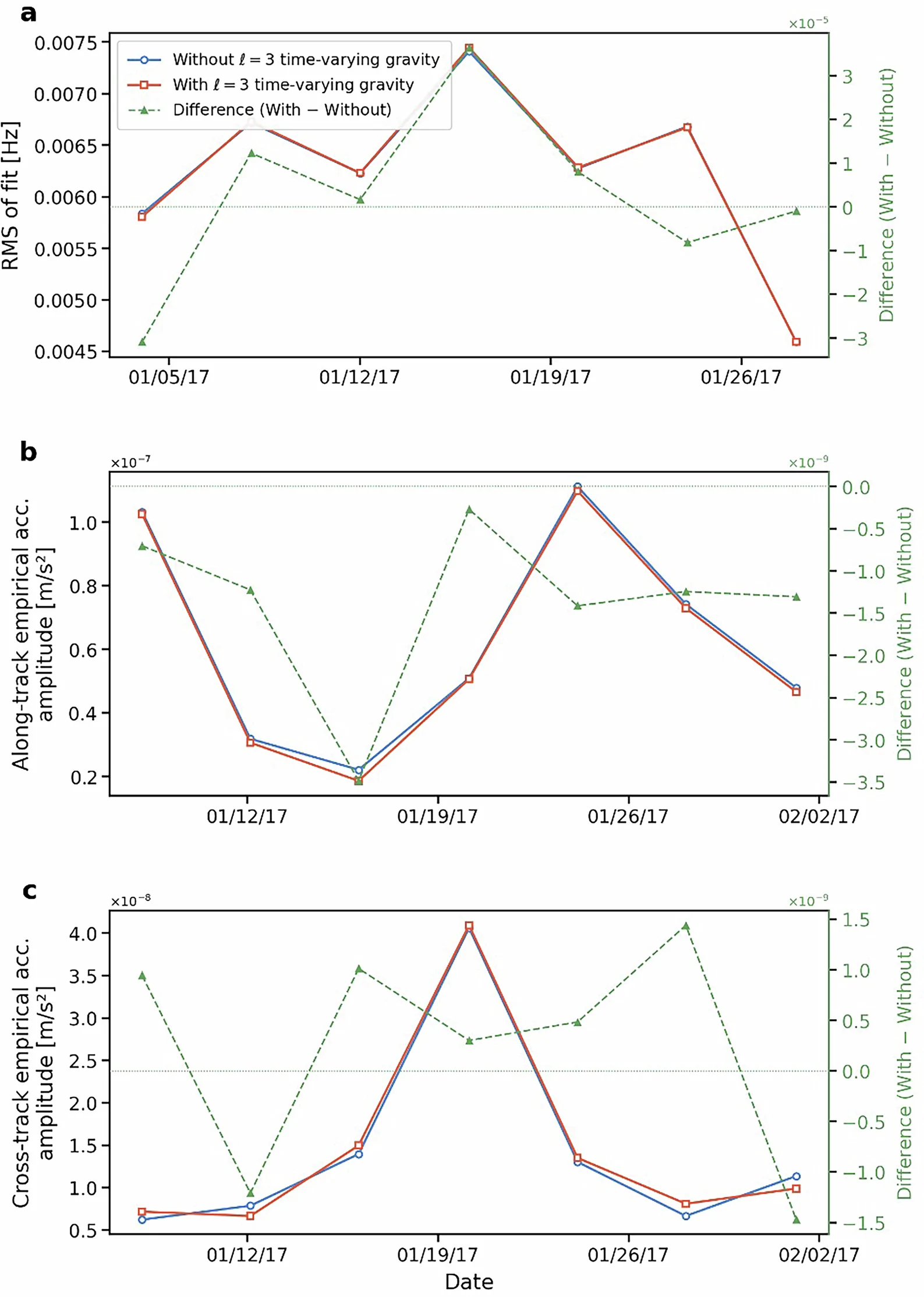
Post-fit residuals and empirical acceleration amplitude change with inclusion of *ℓ* = 3 time-variable gravity. Comparison of gravity field inversion solutions without (blue) and with (red) inclusion of degree-3 time-varying gravity terms; the green dashed line (right axis) shows the difference between solutions. Arcs drawn from 2017 data are not used in the gravity field determination. **a**, r.m.s. of fit (Hz). **b**, Along-track empirical acceleration amplitude. **c**, Cross-track empirical acceleration amplitude.

Estimation of the gravitational coefficients was achieved through a batch least-squares analysis using orbital arcs of 2.5–8.0 days, with the arc length varying according to the mission phase. For each arc, partial derivatives of the Doppler observables were computed with respect to the estimated parameters and a global simultaneous inversion of all arcs and all parameters at once was performed to minimize the residuals between the observed and modelled tracking data. Our analysis represents an extension of previous analyses focused mainly on evaluating the temporal evolution of the zonal terms^28,56^. Results for the degree-3 and degree-2 zonal terms do not change beyond 2*σ* uncertainty when adding the non-zonal terms. Moreover, estimating only degree-3 time-varying terms or explicitly including non-zonal degree-2 terms does not substantially change degree-3 terms inferred from our analysis. As a further check, we performed a sensitivity analysis of post-fit residuals and empirical accelerations to the inclusion of the degree-3 time-variable gravity field using 2017 tracking data not used in the gravity field determination. The r.m.s. of fit for these independent test arcs improves only marginally (<1%) on inclusion of the degree-3 time-varying terms (Extended Data Fig. 7a). This is expected: long-period tidal parameters are constrained by stacking many years of data, so no individual arc is expected to show a marked improvement in fit. Similarly, the a posteriori amplitudes of the empirical accelerations change only marginally and without a systematic trend when the degree-3 time-varying terms are included (Extended Data Fig. 7b,c).

We correct for the contribution of the atmosphere on the Martian gravity field. To do this, we incorporate a time series of spherical harmonic coefficients representing the atmospheric load within the orbit determination procedure (see equation (4) in ref. ^28^). At each epoch, we expand the surface pressure *P*_s_(*λ*, *ϕ*, *t*) provided by the Mars Climate Database (MCD^57^) and include the solid-body response to this surface load through degree-dependent loading Love numbers ${k}_{{\ell }}^{{\prime} }$:

3 $$\begin{array}{c}\Delta {C}_{{\ell }m}^{{\rm{atm}}}(t)=\frac{3(1+{k}_{{\ell }}^{{\prime} })}{4{\rm{\pi }}\bar{\rho }g{R}_{m}(2{\ell }+1)}\oint {P}_{{\rm{s}}}(\lambda ,\phi ,t){P}_{{\ell }m}(\sin \phi )\cos (m\lambda ){\rm{d}}\Omega ,\\ \Delta {S}_{{\ell }m}^{{\rm{atm}}}(t)=\frac{3(1+{k}_{{\ell }}^{{\prime} })}{4{\rm{\pi }}\bar{\rho }g{R}_{m}(2{\ell }+1)}\oint {P}_{{\rm{s}}}(\lambda ,\phi ,t){P}_{{\ell }m}(\sin \phi )\sin (m\lambda ){\rm{d}}\Omega ,\end{array}$$

in which $\bar{\rho }$ is Mars’s mean density, *g* is Mars’s mean surface gravity, *P*_*ℓm*_(sin*ϕ*) are the same fully normalized associated Legendre functions used in equation (1), *P*_s_(*λ*, *ϕ*, *t*) is the instantaneous surface pressure field and d*Ω* = cos*ϕ*d*λ*d*ϕ* is the element of solid angle on the unit sphere. The integrals project *P*_s_(*λ*, *ϕ*, *t*) onto the cosine and sine basis of equation (2) and the factor $(1+{k}_{{\ell }}^{{\prime} })$ accounts for the deformation of the solid planet under this surface load. The loading Love numbers ${k}_{{\ell }}^{{\prime} }$ are computed for a spherically symmetric, self-gravitating 1D elastic Mars using the semi-analytic method described in ref. ^58^. The radially stratified reference interior used for this calculation is described in Extended Data Table 2 and yields ${k}_{2}^{{\prime} }=-0.14126$ and ${k}_{3}^{{\prime} }=-0.09109$. We note that atmospheric surface loads at *ℓ* = 2 and *ℓ* = 3 are comparable in magnitude, so mode coupling between harmonics (that is, *ℓ* = 2 to *ℓ* = 3) driven by lateral heterogeneity negligibly affect ${k}_{2}^{{\prime} }$ and ${k}_{3}^{{\prime} }$. For consistency, we also tested changes to the within-harmonic (diagonal) response of a laterally heterogeneous Martian interior with degree-1, order-(−1, 0, 1) shear modulus variations of 36%, 60%, 42% in the mantle (Fig. 2) using a finite-element code^3,59–65^. This model returns ${k}_{2}^{{\prime} }$ and ${k}_{3}^{{\prime} }$ values that agree with parameters from the 1D reference model to within <1%. We also test the impact of varying ${k}_{{\ell }}^{{\prime} }$ by 200% and 0% (relative to baseline values; ‘0%’ indicates a case with no deformational response to surface loads) on inversion results (Fig. 2) and find that these changes to assumed ${k}_{{\ell }}^{{\prime} }$ influence the median of recovered degree-1 shear modulus coefficient values by <10% (Extended Data Fig. 2).

The spherical harmonic expansions for the atmosphere of Mars in equation (3) are evaluated at 2-h intervals to capture diurnal and semidiurnal components and are included in the dynamical modelling of each spacecraft. The MCD and the Drag Temperature Model-Mars (DTM-Mars^66^) used for modelling non-gravitational forces (see below) apply to distinct altitude regimes (the lower atmosphere and the thermosphere, respectively) but share a common calibration against Mars atmospheric observations (and physical assumptions) and are therefore internally consistent for this analysis. The final retrieved static and time-varying gravity fields corrected for the atmosphere, given in equations (1) and (2) and Table 1 and shown as blue bars in Fig. 1, are therefore representative of the solid-body response only. Variations in the retrieved $\Delta {C}_{{\ell }m}^{A,B}$ and $\Delta {S}_{{\ell }m}^{A,B}$ across these permutations remain stable on multi-year timescales (Extended Data Fig. 1a) and are generally smaller than the observational uncertainties reported in Fig. 1a, Table 1 and Extended Data Table 1 (Extended Data Fig. 1b); we correspondingly do not propagate these variations into the uncertainties for the atmosphere-corrected observations used as constraints on tidal tomography inversions. We do not correct for the seasonal condensation of atmospheric CO_2_ onto the surface (or associated deformational response) for non-zonal harmonics because related gravity perturbations, from both mass redistribution and surface deflection, are expected to be several orders of magnitude smaller than the observations in Table 1 or Extended Data Table 1 (refs. ^58,67^).

Past studies have tested the impact of several more modelling choices for the Martian atmosphere, including the incident solar flux (parameterized by means of the proxy quantity ‘extreme ultraviolet radiation’ or EUV) and the level of atmospheric dust opacity^28,56^. For example, Genova et al.^28^ find that, whereas the incident solar flux has a monotonic impact on gravity coefficients, dust opacity tends to amplify the impact of changes in incident solar flux on the time-variable gravity coefficients of Mars. Therefore, as an extra check, we reprocessed the atmospheric contributions from equation (3) to compare our baseline model (an average solar flux scenario, ‘Avg EUV’, *F*_10.7_ ≈ 130 sfu (ref. ^57^)) against endmember scenarios drawn from the MCD: (1) a cold-atmosphere case with minimum solar flux (*F*_10.7_ ≈ 70 sfu or solar flux units, for which 1 sfu = 10^−22^ W m^−2^ Hz^−1^ (ref. ^57^)) combined with dust-storm conditions (column-integrated visible optical depth *τ* ≳ 5 (ref. ^68^)) or ‘Cold min EUV dust’ and (2) a warm-atmosphere case with maximum solar flux (*F*_10.7_ ≈ 200 sfu (ref. ^57^)) combined with the same dust opacity or ‘Warm max EUV dust’. These differences between atmospheric models, when applied as corrections to the atmosphere-corrected observations shown in Fig. 1, propagate into ≤20% shifts in the inferred posterior distributions of degree-1 mantle shear modulus relative to our nominal inversion (Extended Data Fig. 2). The most substantial shifts (relative to the nominal case) occur for the ‘Cold min EUV dust’ scenario, although recovered posteriors for this case indicate a north–south pattern that still broadly aligns with the structure of the crustal dichotomy (degree-1 order-0 and degree-1 order-1 variations remain significant at 3*σ* and 2*σ* levels, respectively).

We correct for non-gravitational forces on each spacecraft with a detailed forward model of the non-conservative perturbations. To this end, we represent each spacecraft as a multi-panel body and assign thermo-optical properties to movable surfaces such as the high-gain antenna and solar arrays. We predict the thermospheric density along each orbit with DTM-Mars^66^ updated for the revised evolution of the main atmospheric constituents^69^. To absorb any residual mismodelling, we also co-estimate small periodic accelerations in the along-track and cross-track directions at frequencies tied to the orbital period, following ref. ^28^. We note that, although tidal forcing is theoretically stronger at diurnal frequencies than at the seasonal period^1^, the higher-frequency components of the time-variable gravity of Mars are also the most susceptible to residual non-gravitational errors. Estimated empirical accelerations therefore largely absorb diurnal tidal signals together with the perturbations they target^28,56^. For this reason, we do not attempt to recover diurnal tidal signals and restrict our analysis to the annual gravity field variations.

### Modelling the tidal deformation of Mars

We directly model the impact of tidal deformation associated with Sun–Mars tides on coefficients Δ*C*_*ℓm*_ and Δ*S*_*ℓm*_ according to (superscript ‘p’ denotes ‘predicted’):

4 $$\Delta {C}_{{\ell }m}^{{\rm{p}}}-{\rm{i}}\Delta {S}_{{\ell }m}^{{\rm{p}}}={f}^{{\rm{p}}}(t)$$

in which:

5 $$\begin{array}{c}{f}^{{\rm{p}}}(t)=\frac{1}{{N}_{{\ell }m}}\mathop{\sum }\limits_{{{\ell }}^{{\prime} }=2}^{3}\mathop{\sum }\limits_{{m}^{{\prime} }=0}^{{{\ell }}^{{\prime} }}\frac{1}{2{{\ell }}^{{\prime} }+1}\frac{G{M}_{{\rm{S}}}}{{GM}}\frac{{R}^{{{\ell }}^{{\prime} }+1}}{{r}_{{\rm{S}}}^{{{\ell }}^{{\prime} }+1}}{P}_{{{\ell }}^{{\prime} }{m}^{{\prime} }}(\sin {\phi }_{S})\\ \,\times \left[({K}_{{{\ell }}^{{\prime} },{m}^{{\prime} }}^{{\ell },m}\cos ({m}^{{\prime} }{\lambda }_{{\rm{S}}})+{K}_{{{\ell }}^{{\prime} },-{m}^{{\prime} }}^{{\ell },m}\sin ({m}^{{\prime} }{\lambda }_{{\rm{S}}}))\right.\\ \,\left.-{\rm{i}}({K}_{{{\ell }}^{{\prime} },{m}^{{\prime} }}^{{\ell },-m}\cos ({m}^{{\prime} }{\lambda }_{{\rm{S}}})+{K}_{{{\ell }}^{{\prime} },-{m}^{{\prime} }}^{{\ell },-m}\sin ({m}^{{\prime} }{\lambda }_{{\rm{S}}}))\right].\end{array}$$

In equation (5), *M*_S_ is the mass of the Sun and (*λ*_S_, *ϕ*_S_, *r*_S_) represent solar longitude, latitude and distance, respectively, in the body-fixed frame of Mars. We evaluate (*λ*_S_, *ϕ*_S_, *r*_S_) based on solar and Martian ephemerides extracted from NASA’s Planetary Data System evaluated over the mission durations of the MGS, ODY and MRO spacecraft. Note that equation(5 ) accounts for tides that arise from both Mars’s 0.0934 eccentricity and 25.2° spin-axis obliquity relative to the Sun through the impact of these orbital parameters on variations in *λ*_S_, *ϕ*_S_, *r*_S_. *N*_*ℓm*_ is the normalization factor:

6 $${N}_{{\ell }m}=\sqrt{\frac{({\ell }-m)!\,(2-{\delta }_{0m})(2{\ell }+1)}{({\ell }+m)!}}$$

${K}_{{{\ell }}^{{\prime} },{m}^{{\prime} }}^{{\ell },m}$ in equation (5) denotes ‘extended Love numbers’ (distinct from traditional Love numbers *k*_*ℓm*_) that represents coupling between forcing at one harmonic (*ℓ*′, *m*′) and the gravitational response to this forcing at another harmonic (*ℓ*, *m*) for a given interior structure. We compute ${K}_{{{\ell }}^{{\prime} },{m}^{{\prime} }}^{{\ell },m}$ using the semi-analytic spectral method LOV3D (ref. ^10^) (see also refs. ^70,71^), which solves mass conservation, momentum and Poisson’s equations in the Fourier domain for a laterally heterogeneous body subject to tidal loading^3,59–62^. Given ${K}_{{{\ell }}^{{\prime} },{m}^{{\prime} }}^{{\ell },m}$, we compute $\Delta {C}_{{\ell }m}^{A,B,p}$ and $\Delta {S}_{{\ell }m}^{A,B,p}$ by evaluating *f*^p^(*t*) in equation (5) and then projecting this quantity into cosine and sine functions evaluated over the Martian annual period:

7 $$\begin{array}{c}\Delta {C}_{{\ell }m}^{A,{\rm{p}}}={\rm{\Re }}\,\left(\frac{\omega }{2{\rm{\pi }}}{\int }_{{t}_{{\rm{start}}}}^{{t}_{{\rm{end}}}}{f}^{{\rm{p}}}(t)\cos (\omega t){\rm{d}}t\right)\\ \Delta {C}_{{\ell }m}^{B,{\rm{p}}}={\rm{\Re }}\,\left(\frac{\omega }{2{\rm{\pi }}}{\int }_{{t}_{{\rm{start}}}}^{{t}_{{\rm{end}}}}{f}^{{\rm{p}}}(t)\sin (\omega t){\rm{d}}t\right)\\ \Delta {S}_{{\ell }m}^{A,{\rm{p}}}={\rm{\Im }}\,\left(-\frac{\omega }{2{\rm{\pi }}}{\int }_{{t}_{{\rm{start}}}}^{{t}_{{\rm{end}}}}{f}^{{\rm{p}}}(t)\cos (\omega t){\rm{d}}t\right)\\ \Delta {S}_{{\ell }m}^{B,{\rm{p}}}={\rm{\Im }}\,\left(-\frac{\omega }{2{\rm{\pi }}}{\int }_{{t}_{{\rm{start}}}}^{{t}_{{\rm{end}}}}{f}^{{\rm{p}}}(t)\sin (\omega t){\rm{d}}t\right).\end{array}$$

Note that equation (7) captures only the solid-body tidal response. However, zonal coefficients are also sensitive to seasonal CO_2_ exchange between the polar caps, which we model separately:

8 $$\Delta {C}_{{\ell }0}^{A,B,{\rm{p}}}=\Delta {C}_{{\ell }0}^{A,B,{\rm{p}},{\rm{solid}}}+\Delta {C}_{{\ell }0}^{A,B,{\rm{p}},{\rm{poles}}},$$

in which $\Delta {C}_{{\ell }0}^{A,B,{\rm{p}},{\rm{solid}}}$ is computed from equation (7) and the polar-cap contribution $\Delta {C}_{{\ell }0}^{A,B,{\rm{p}},{\rm{poles}}}$ is computed by representing the seasonal mass exchange as time-varying point masses Δ*m*_N_(*t*) and Δ*m*_S_(*t*) located at the north and south rotation poles, respectively. For point masses on the rotation axis, the corresponding (4π-normalized) coefficient is:

9 $$\Delta {C}_{{\ell }0}^{{\rm{poles}}}(t)=\frac{1}{M\sqrt{2{\ell }+1}}[\Delta {m}_{{\rm{N}}}(t)+{(-1)}^{{\ell }}\Delta {m}_{{\rm{S}}}(t)],$$

which, for the degree-2 and degree-3 zonal terms, reduces to

10 $$\Delta {C}_{20}^{{\rm{poles}}}(t)=\frac{\Delta {m}_{{\rm{N}}}(t)+\Delta {m}_{{\rm{S}}}(t)}{M\sqrt{5}},\,\Delta {C}_{30}^{{\rm{poles}}}(t)=\frac{\Delta {m}_{{\rm{N}}}(t)-\Delta {m}_{{\rm{S}}}(t)}{M\sqrt{7}}.$$

We use seasonal mass amplitudes of |Δ*m*_N_| = 6.2 × 10^15^ kg and |Δ*m*_S_| = 8.4 × 10^15^ kg (ref. ^28^), with Δ*m*_N_(*t*) and Δ*m*_S_(*t*) varying anti-phase over the Martian year (mass accumulates at one pole as it sublimates from the other). The harmonic components $\Delta {C}_{{\ell }0}^{A,B,{\rm{p}},{\rm{poles}}}$ are then obtained by projecting $\Delta {C}_{{\ell }0}^{{\rm{poles}}}(t)$ onto cos(*ωt*) and sin(*ωt*) following equation (7). The values of |Δ*m*_N_| and |Δ*m*_S_| from ref. ^28^ are empirically calibrated and implicitly absorb the solid-body loading response of the interior to seasonal polar-cap mass exchange. As an extra check of the sensitivity of the inclusion of zonal terms, we perform a tidal tomography inversion that entirely excludes zonal harmonics as constraints. The resulting posterior distributions for this scenario deviate by less than 15% from those of our nominal inversion (Extended Data Fig. 2).

### Tidal tomography procedure

To constrain the structure of the Martian interior, we carry out a Bayesian inversion with Markov Chain Monte Carlo and a Metropolis sampling algorithm using PyMC (ref. ^72^). For our inversion, we vary elastic parameters relative to a reference Martian interior to fit observed $\Delta {C}_{{\ell }m}^{A,B}$ and $\Delta {S}_{{\ell }m}^{A,B}$ (Table 1). Our detailed procedure is described below.

We consider the impact of 3D perturbations to the shear modulus of a 1D reference model on $\Delta {C}_{{\ell }m}^{A,B}$ and $\Delta {S}_{{\ell }m}^{A,B}$. Our reference model is fixed (that is, not sampled) and is based on the analysis of ref. ^29^, which constrains Martian interior structure using the planet’s mean density, moment of inertia, seismic wave arrival times and the predicted composition of the Martian mantle (see Extended Data Table 2 for assumed mean shear modulus, bulk modulus and density values for each internal layer). We do not consider lateral changes in density or bulk modulus (the latter of which has a negligible effect on $\Delta {C}_{{\ell }m}^{A,B}$ and $\Delta {S}_{{\ell }m}^{A,B}$; see ref. ^9^) for tidal tomography inversions (COM–COF offset is used only for a posteriori calculations described in Fig. 3). Because shear modulus (*μ*) is related to shear wave speed (*V*_s_) and density (*ρ*) through $\mu =\rho {V}_{{\rm{s}}}^{2}$, our approach effectively perturbs *V*_s_ while maintaining fixed *ρ* within each model layer. Note that our choice of reference model could substantially affect the non-coupled response of the interior of Mars (that is, ${K}_{{{\ell }}^{{\prime} },{m}^{{\prime} }}^{{\ell },m}$, in which *ℓ*, *m* = *ℓ*′, *m*′). However, from equation (5), forcing at *ℓ* = 3 harmonics is expected to be negligible (approximately three orders of magnitude smaller) compared with mode-coupled responses for these harmonics reported in Table 1 and so are ignored for inversions.

We consider a total of 30 parameters that describe 3D variations in shear modulus (that is, *ℓ* = 1–3 for the crust and mantle). These parameters are sampled as coefficients for spherical harmonic basis functions that comprise the ratio of the spatially variable shear modulus *μ* to that of the fixed reference model *μ*_ref_ for each internal layer:

11 $$\begin{array}{l}\frac{\mu }{{\mu }_{{\rm{ref}}}}=\mathop{\sum }\limits_{{\ell }=1}^{3}\left[{\psi }_{{\ell }0}\,{\mu }_{{\ell }0}{Y}_{{\ell }0}(\theta ,\lambda )\right.\\ \,+\,\mathop{\sum }\limits_{m=1}^{{\ell }}\left.({\psi }_{{\ell }m}^{{\rm{c}}}\,{\mu }_{{\ell }m}^{{\rm{c}}}{Y}_{{\ell }m}^{{\rm{c}}}(\theta ,\lambda )+{\psi }_{{\ell }m}^{{\rm{s}}}\,{\mu }_{{\ell }m}^{{\rm{s}}}{Y}_{{\ell }m}^{{\rm{s}}}(\theta ,\lambda ))\right],\end{array}$$

in which the sampled coefficients are *μ*_*ℓ*0_ for *m* = 0 and $({\mu }_{{\ell }m}^{{\rm{c}}},{\mu }_{{\ell }m}^{{\rm{s}}})$ for *m* ≥ 1. By normalizing 3D shear modulus variations by *μ*_ref_ in equation (11), we implicitly account for the impact of variations in 1D shear modulus on mode-coupled responses such that our choice of reference model does not substantively affect inversion results. As an extra check, we have carried out tidal tomography inversions for which we sample the 1D shear and bulk moduli of the crust and mantle from a Gaussian distribution centred about our nominal reference model values (Extended Data Table 3) and with a standard deviation equal to 5% of these values (Extended Data Fig. 2). The resulting posterior distributions for recovered degree-1 variations in mantle shear modulus deviate minimally from those of our nominal inversion.

The real-form spherical harmonic basis functions (with *θ* the planetocentric co-latitude in the COM frame) are:

12 $$\begin{array}{c}{Y}_{{\ell }0}(\theta ,\lambda )=\sqrt{\frac{2{\ell }+1}{4{\rm{\pi }}}}{P}_{{\ell }0}(\cos \theta ),\\ {Y}_{{\ell }m}^{{\rm{c}}}(\theta ,\lambda )=\left\{\begin{array}{cc}\sqrt{\frac{(2{\ell }+1)}{2{\rm{\pi }}}\frac{({\ell }-m)!}{({\ell }+m)!}}{P}_{{\ell }m}(\cos \theta )\cos (m\lambda ),\, & m > 0,\\ 0,\, & m=0,\,\\ \,\end{array}\right.\\ {Y}_{{\ell }m}^{{\rm{s}}}(\theta ,\lambda )=\left\{\begin{array}{cc}\sqrt{\frac{(2{\ell }+1)}{2{\rm{\pi }}}\frac{({\ell }-m)!}{({\ell }+m)!}}{P}_{{\ell }m}(\cos \theta )\sin (m\lambda ),\, & m > 0,\\ 0,\, & m=0.\end{array}\right.\end{array}$$

The quantities *ψ*_*ℓ*0_, ${\psi }_{{\ell }m}^{{\rm{c}}}$ and ${\psi }_{{\ell }m}^{{\rm{s}}}$ indicate coefficients that normalize the right side of equation (11) so that an input ${\mu }_{{\ell }m},{\mu }_{{\ell }m}^{{\rm{c}}},$ or ${\mu }_{{\ell }m}^{{\rm{s}}}$ of 1 (assuming that all other sampled coefficients are zero) corresponds to a peak-to-peak variation in *μ*/*μ*_ref_ of −1 to 1 (see, for example, equation (68) in ref. ^9^). Note that values presented in Fig. 2a–c indicate ${\mu }_{{\ell }m},{\mu }_{{\ell }m}^{{\rm{c}}},{\mu }_{{\ell }m}^{{\rm{s}}}\times 100{\rm{ \% }}$. To generate prior distributions, we parameterize ${\mu }_{{\ell }m},{\mu }_{{\ell }m}^{{\rm{c}}},{\mu }_{{\ell }m}^{{\rm{s}}}$ in equation (11) in terms of their base-10 logarithm (that is, ${\mu }_{{\ell }m}^{{\prime} },{\mu }_{{\ell }m}^{{{\rm{c}}}^{{\prime} }},{\mu }_{{\ell }m}^{{{\rm{s}}}^{{\prime} }}$):

13 $$\begin{array}{l}{\mu }_{{\ell }m}^{{\prime} }\,=\,{\log }_{10}(1+{\mu }_{{\ell }m}),\\ \,{\mu }_{{\ell }m}^{{{\rm{c}}}^{{\prime} }}\,=\,{\log }_{10}(1+{\mu }_{{\ell }m}^{{\rm{c}}}),\\ \,{\mu }_{{\ell }m}^{{{\rm{s}}}^{{\prime} }}\,=\,{\log }_{10}(1+{\mu }_{{\ell }m}^{{\rm{s}}}).\end{array}$$

To generate ensembles of internal structure models for Markov chains, we sample uniform (that is, flat) prior probability distributions for ${\mu }_{{\ell }m}^{{\prime} },{\mu }_{{\ell }m}^{{{\rm{c}}}^{{\prime} }},{\mu }_{{\ell }m}^{{{\rm{s}}}^{{\prime} }}$ (that correspond to a range of ${\mu }_{{\ell }m},{\mu }_{{\ell }m}^{{\rm{c}}},{\mu }_{{\ell }m}^{{\rm{s}}}\times 100{\rm{ \% }}$ from −200% to 200%) and forward compute $\Delta {C}_{{\ell }m}^{A,B,{\rm{p}}}$ and $\Delta {S}_{{\ell }m}^{A,B,{\rm{p}}}$ using these values. Each ensemble consists of about 10,000 individual accepted model realizations (that is, approximately 500,000 samples total from 50 walkers). To speed up convergence, we use an adaptive sampling approach (the ‘tune’ functionality in PyMC) that dynamically adjusts step sizes based on the sensitivity of model outputs to input parameters^72^. We visually inspect Markov chains to discard initial burn-in steps (that is, typically the first roughly 10–20% of samples) and terminate inversions when parameter autocorrelation values are >0.99. Walker positions are updated on the basis of the likelihood function *L*:

14 $$\log L\propto -\frac{1}{2}{({\bf{X}}-{\bf{Y}})}^{{\rm{T}}}{{\boldsymbol{\Sigma }}}^{-1}({\bf{X}}-{\bf{Y}}),$$

in which **X** is the vector of observed $\Delta {C}_{{\ell }m}^{A,B}$ and $\Delta {S}_{{\ell }m}^{A,B}$ in equation (2) (that is, parameters in Table 1 and Extended Data Table 1) and **Y** is the vector of model-predicted $\Delta {C}_{{\ell }m}^{A,B,{\rm{p}}}$ and $\Delta {S}_{{\ell }m}^{A,B,{\rm{p}}}$ in equation (7). For a comparison of model-predicted posteriors of $\Delta {C}_{{\ell }m}^{A,B,{\rm{p}}}$ and $\Delta {S}_{{\ell }m}^{A,B,{\rm{p}}}$ to observations, see Extended Data Fig. 3. **Σ** is a diagonal matrix (that is, each entry is independent) that incorporates observational covariances (that is, 15× formal uncertainties presented in Table 1). Other potential system constraints (for example, mean density, moment of inertia or quality factors) are not incorporated into vectors **X** or **Y** in equation (14).

### Modelling temperature and iron content

In this section, we outline our methodology for the calculations shown in Fig. 3. We parameterize hemispheric variations in effective shear modulus in terms of the combined effect of temperature and composition on rheology:

15 $$\Delta \mu =\Delta T\frac{{\rm{d}}\mu }{{\rm{d}}T}+\Delta {X}_{{\rm{Fe}}}\frac{{\rm{d}}\mu }{{\rm{d}}{X}_{{\rm{Fe}}}},$$

in which Δ*T* is the hemispheric temperature contrast and Δ*X*_Fe_ is the hemispheric contrast in fayalite–forsterite mole fraction (Fa–Fo). To compute $\frac{{\rm{d}}\mu }{{\rm{d}}T}$, we fit shear modulus versus forcing timescale data from Fig. 1a of ref. ^34^ (the ‘background-only’ Burgers model; note that all other calculations for tidal tomography inversions assume a purely elastic rheology) with power-law functions and extend these curves to the Martian annual forcing period (5.94 × 10^7^ s; Extended Data Fig. 6) yielding:

16 $$\frac{{\rm{d}}\mu }{{\rm{d}}T}\approx -0.204\,{\rm{GPa}}\,{{\rm{K}}}^{-1}.$$

We note that several anelastic laws have been published (for example, refs. ^73,74^) but we have chosen that of ref. ^34^, as it has been used in several analogous applications for the Moon^75^, Mars^76^ and the Earth^77,78^. Although each anelastic law differs in details, the overall trend of shear modulus softening with lower frequency across all experimental laws is consistent. For compositional variations, we use $\frac{{\rm{d}}\mu }{{\rm{d}}{X}_{{\rm{Fe}}}}=-0.3\,{\rm{GPa}}\,{\rm{per}}\,{\rm{ \% }}$ Fa–Fo based on experimental data presented in ref. ^79^.

We evaluate the impact of degree-1 variations in density on the COM–COF offset of Mars (that is, *δz*_COM-COF_) by first assuming an offset vector of the form:

17 $$\delta {z}_{\mathrm{COM-COF}}=\frac{1}{M}{\int }_{V}z\rho ({\bf{r}}){\rm{d}}V,$$

in which **r** is the position vector within Mars, *ρ*(**r**) is the local density, *z* is the component of **r** along the asymmetry axis, *V* is the volume of the planet and *M* is its mass. Converting equation (17) into spherical harmonics and integrating over the radial extent of the mantle, we obtain:

18 $$\delta {z}_{\mathrm{COM-COF}}=\frac{1}{M}\sqrt{\frac{{\rm{\pi }}}{12}}\Delta \rho ({r}_{{\rm{Moho}}}^{4}-{r}_{{\rm{CMB}}}^{4}),$$

in which *M* = 6.4171 × 10^23^ kg is the mass of Mars, *r*_Moho_ and *r*_CMB_ are the radii of the Martian Moho and core–mantle boundary, respectively, and Δ*ρ* is the peak-to-peak amplitude of degree-1 variations in mantle density. We compute Δ*ρ* by assuming linear contributions from thermal expansion and iron enrichment:

19 $$\Delta \rho =-{\rho }_{{\rm{ref}}}\beta \Delta T+\Delta {X}_{{\rm{Fe}}}\frac{{\rm{d}}\rho }{{\rm{d}}{X}_{{\rm{Fe}}}},$$

in which *ρ*_ref_ = 3,500 kg m^−3^ is a representative mantle density, *β* = 3 × 10^−5^ K^−1^ is the volume thermal expansion coefficient and $\frac{{\rm{d}}\rho }{{\rm{d}}{X}_{{\rm{Fe}}}}=9.7\,{\rm{kg}}\,{{\rm{m}}}^{-3}\,{\rm{per}}\,{\rm{ \% }}$ Fa–Fo is the assumed density sensitivity to iron enrichment, following ref. ^4^. Equation (19) is formulated in terms of lateral density perturbations relative to a reference mantle, so the absolute mantle density and baseline iron content do not directly affect the calculation except insofar as they modify the adopted transfer function $\frac{{\rm{d}}\rho }{{\rm{d}}{X}_{{\rm{Fe}}}}$.

For comparison with these calculations, we use COM–COF offsets from Table 4 of ref. ^35^, which gives Δ*x* = 233 m, Δ*y* = 1,428 m and Δ*z* = 2,986 m, corresponding to a total offset magnitude of 3.3 km. Although Smith et al.^35^ do not report a formal uncertainty for this quantity, uncertainties are expected to be small (<0.02 km) relative to the 0–1.31-km range used here. We project the vector of the COM–COF offset onto the axis defined by the maximum shear modulus asymmetry in Fig. 2d (45° N, 138° W), which yields an upper-bound contribution of density variations in the mantle to COM–COF offset of 1.31 km. The lower bound of 0 km used in Fig. 3 corresponds to the opposite endmember case in which the observed COM–COF offset is explained entirely by crustal structure (for example, a case in which surface topography produces a COF shift of 1.31 km).

We note that the 0–1.31-km range is an approximate (that is, order-of-magnitude) estimate of the impact of mantle density on COM–COF offset and therefore does not encompass the full range of possible degree-1 density variations in the interior. For example, Moho relief can change the COM–COF offset by >50% if this effect is not negated by the impact of surface topography on the COM (for example, by means of Airy isostasy). Similarly, the depth dependence of $\frac{{\rm{d}}\rho }{{\rm{d}}{X}_{{\rm{Fe}}}}$, including the impact of a possible density crossover near 600–700 km depth, could change the COM–COF offset by up to several hundred metres. Even so, these effects (and the 0–1.31-km range) are much smaller than the several tens of kilometres in COM–COF offset that would be required to explain the asymmetries shown in Fig. 3 purely in terms of compositional variations (for example, iron enrichment) in the mantle.

### Martian melt ascent

Here we estimate the approximate timescale of ascent for melt for the mantle of Mars. McKenzie^43^ gives the characteristic timescale *Λ* for a small melt fraction to separate from a partially molten layer of thickness *h*:

20 $$\varLambda =\frac{h\eta \psi }{\kappa \Delta \rho g}$$

in which *η* is the melt viscosity, *ψ* is the melt fraction, Δ*ρ* is the density contrast between melt and matrix, *g* is the acceleration owing to gravity and *κ* is the permeability. For small melt fractions, the permeability is taken to be:

21 $$\kappa =\frac{{d}^{2}\,{\psi }^{n}}{C}$$

Here *d* is the grain size, *n* = 2 and *C* = 3,000. For the Martian mantle, we will take *h* = 300 km, Δ*ρ* = 100 kg m^−3^, *g* = 3.7 m s^−2^ and a grain size of 1 mm. If the areotherm crosses the solidus at 300 km depth, the temperature of the melts will be in the range 1,700–1,800 K. The resulting viscosity of Martian basaltic melts at these temperatures is roughly 1 Pa s (ref. ^80^). For a melt fraction of 0.02, the permeability is then about 10^−13^ m^2^ and the compaction timescale is approximately 3 Myr.

### Ethics

This study did not involve human participants, human data or tissue, or animals, and therefore no ethical approval or informed consent was required. The work is based entirely on the analysis of publicly available spacecraft tracking and gravity data, and complies with all relevant institutional, national, and international guidelines and regulations.

## Online content

Any methods, additional references, Nature Portfolio reporting summaries, source data, extended data, supplementary information, acknowledgements, peer review information; details of author contributions and competing interests; and statements of data and code availability are available at 10.1038/s41586-026-10893-x.

## Supplementary information

Peer Review File

## Figures and Tables

**Extended Data Table 1:**
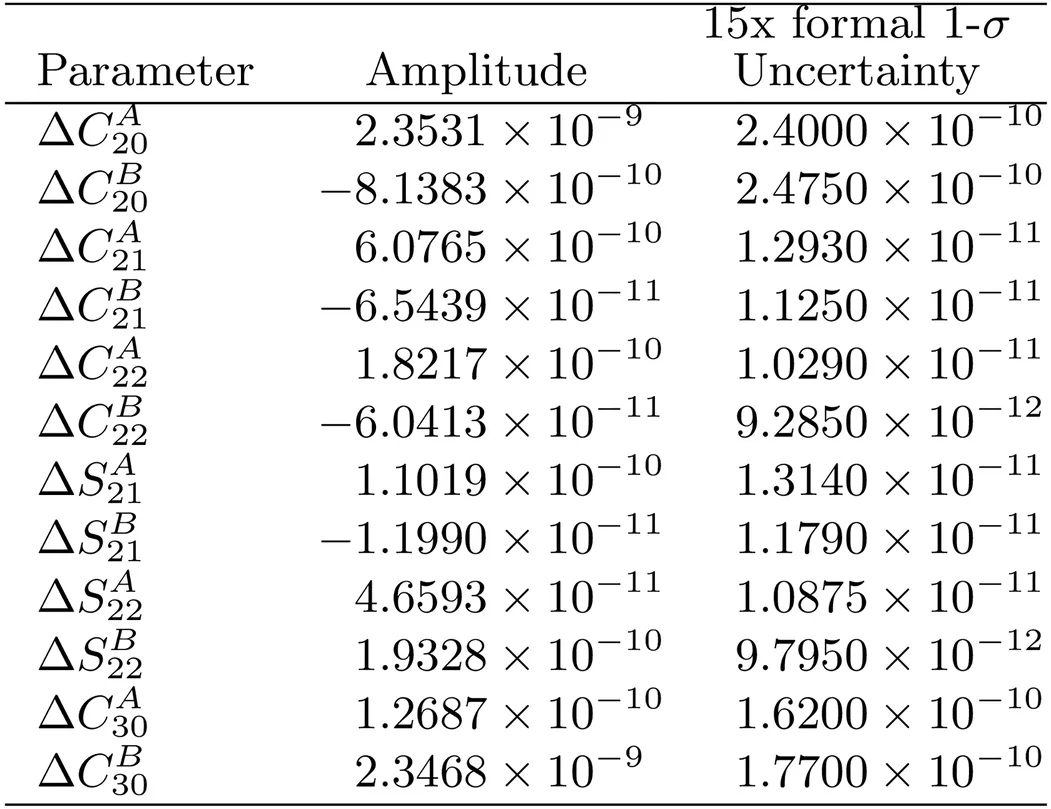
Recovered degree-2 and zonal degree-3 time-varying gravity field for Mars with 15× formal uncertainties

Coefficient amplitudes are 4π-normalized and evaluated over the seasonal, 687-day period referenced to the J2000 epoch. Superscripts *A* and *B* denote in-phase (cosine) and out-of-phase (sine) signal components.

**Extended Data Table 2:**
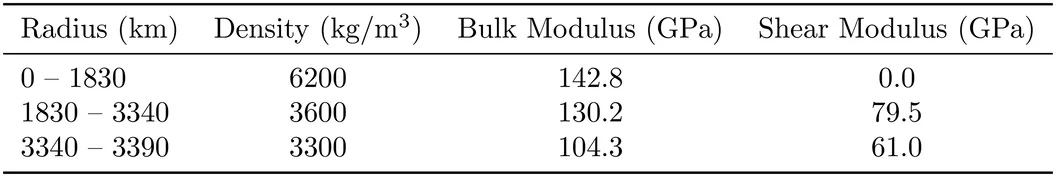
Assumed 1D reference interior model for Mars

Columns indicate the radial range, density, bulk modulus and shear modulus of each layer. The model is constrained by Mars’s degree-2 tidal deformation, moment of inertia, mean density and seismic wave arrival times^29^.

**Extended Data Table 3:**
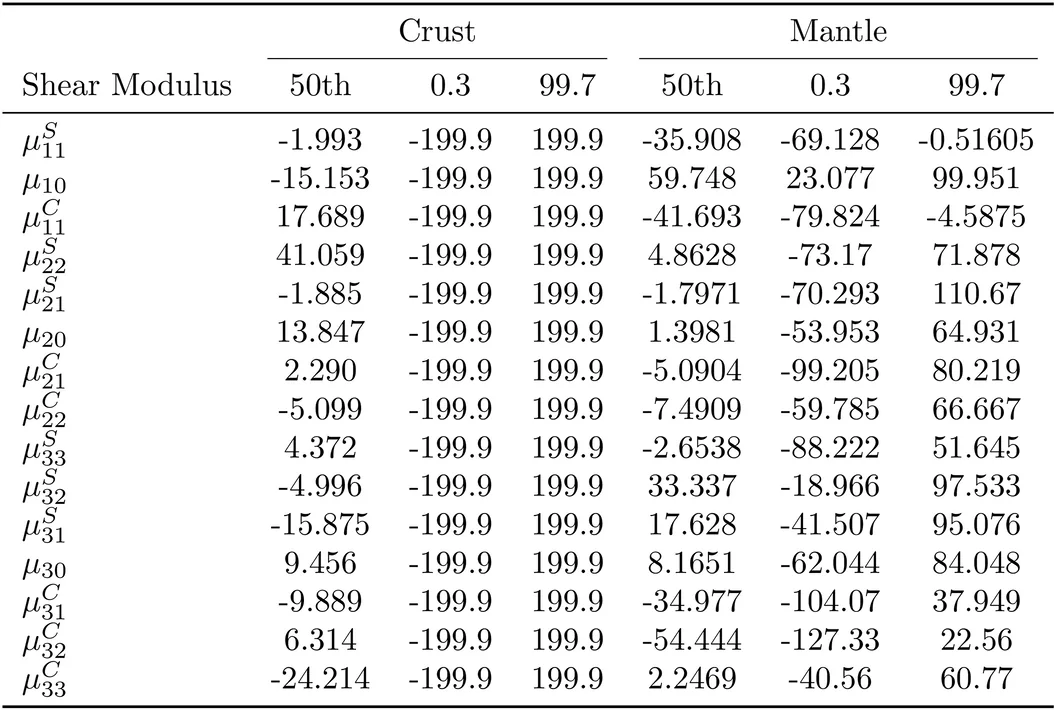
Inverted shear modulus coefficients for the interior of Mars

Columns indicate the 50th, 0.3rd and 99.7th percentile ranges of posterior distributions for coefficients in the crust (left column set) and mantle (right column set).

## Data Availability

The MGS, ODY and MRO data used to generate the results of this paper are available at the NASA Planetary Data System Geosciences Node (http://pds-geosciences.wustl.edu/missions/mgs/rsraw.html, http://pds-geosciences.wustl.edu/missions/odyssey/radioscience.html, http://pds-geosciences.wustl.edu/mro/mro-m-rss-1-magr-v1/mrors_0xxx/).

## Code availability

The gravity recovery results presented in this study were generated using the GEODYN II orbit determination and geodetic parameter estimation software^55^. The publicly distributed NASA build of GEODYN can be obtained from https://space-geodesy.nasa.gov/techniques/tools/GEODYN/GEODYN.html. The software LOV3D is also available at GitHub (https://github.com/mroviranavarro/LOV3D_multi), and an example script to evaluate gravity coefficients for a trial Martian interior model corresponding to the maximum a posteriori solution from our inversion (and estimates of forcing potential amplitudes based on equation (5) and planetary ephemerides over the MGS, ODY and MRO mission durations) is available from Zenodo (https://zenodo.org/records/20823473 (ref. ^81^)).
