# Supplementary material for: Tidal tomography reveals a thermal anomaly beneath Mars’s crustal dichotomy
Source: Nature. 2026 Aug 26;656(8129):848–53. doi: 10.1038/s41586-026-10893-x (PMC13518231; doi:10.1038/s41586-026-10893-x)
Supplement: Supplementary file 1 [file 41586_2026_10893_MOESM1_ESM.pdf]

nature portfolio

Peer Review File

Tidal Tomography Reveals a Thermal Anomaly Beneath Mars's Crustal Dichotomy

Corresponding Author: Dr Alexander Berne

Any redactions in this file are there to maintain patient confidentiality, the confidentiality of unpublished data, or to remove third-party material.

This file contains all reviewer reports in order by version, followed by all author rebuttals in order by version.

Version 0:

Reviewer comments:

Referee #1

(Remarks to the Author)
This paper analyzes non-zonal 3-degree components of Mars' time-dependent gravity field from Mars Global Surveyor, Mars Odyssey, and Mars Reconnaissance Orbiter. The authors show that, compared to spherically symmetric models, these components deviate by 200%, suggesting an asymmetric distribution of the shear modulus. This variation seems to be consistent with the north-south pattern of Mars' crustal dichotomy. Based on these values, the authors show that the mantle beneath the southern hemisphere has a melt fraction of more than 2%.

The finding of an asymmetrical distribution of the shear modulus, which aligns with crustal dichotomy, is important because it confirms that the crustal dichotomy, which likely formed very early in evolution, continues to influence the thermochemical evolution of Mars to this day. This has already been postulated in several studies, but has not yet been confirmed from data: The thicker crust of the southern hemisphere with a higher concentration of heat sources and lower thermal conductivity than the mantle, results in a thinner lithosphere, higher temperatures, and more prolonged volcanic activity, in contrast to the thinner crust of the northern hemisphere (e.g., Schumacher and Breuer 2006; Plesa et al. 2018; Thiriet et al. 2018, Knapmeyer-Endrun et al. 2021; Plesa et al. 2022; Bonnet-Gibet et al. 2022).

However, the interpretation of the results in the present study differs in that the authors preferred model assumes that the mantle below the southern hemisphere has a melt fraction of more than 2% uniformly over the mantle depth range. In dynamic 3D models (Plesa et al., 2018; Plesa et al. 2022), temperatures below the southern hemisphere are indeed higher than underneath the northern hemisphere but present melting is found to be more localized, in regions of particularly thickened crust, such as below the Tharsis and Elysium region. The models with parameterized convection by Thiriet et al. and Bonnet-Gibet et al. indeed show melting or higher temperature beneath the entire southern hemisphere—however, it should be noted that these models only calculate mean profiles and cannot resolve the mantle convection pattern and the crustal thickness variations. What all these models—3D and parameterized—show, however, is partial melting below the lithosphere and not throughout the entire mantle down to the CMB, contrary to the authors' preferred model or at the CMB if a basal magma ocean does exist. Due to the pressure dependence of the melting temperature, this is not possible, or only under very specific conditions. To my knowledge, there is no model or scenario showing this. Also not the model by Bonnet-Gibet et al., which the authors cite as a possible scenario for the formation of the crustal thickness dichotomy. In addition, it should be noted that the model by Bonnet Gibet et al. is from its concept similar to the other models mentioned above, i.e. crust formation is preferential underneath thickened crust due to thermal insulation of the crust and its enrichment in radiogenic heat sources.

In my opinion, the present study does not achieve what is suggested in some places in the manuscript, namely that it can also explain the origin of the dichotomy (different crust thicknesses in the southern and northern hemispheres). If the authors want to explain the origin of the dichotomy (see lines 60-65) with today's observations, they must explain what differences in internal structure can be expected for these different scenarios. The Bonnet-Gibet et al. model is preferred (but see the point above regarding the degree of uniform melting across the entire mantle depth range). Later, however, it is also stated that impact models cannot be ruled out as the cause of the dichotomy. I suspect that the manuscript's statement that non-zonal 3-degree components indicate a lower shear modulus underneath the southern hemisphere is most likely independent of the

cause of the origin.

At this point, it should also be emphasized that the observation that the southern hemisphere has a higher topography is usually explained by the fact that the crust beneath the southern hemisphere is about 25 km thicker than beneath the northern hemisphere (crustal thickness dichotomy). However, there are alternative models that do not assume a variation in crustal thickness but postulate a lateral variation in crustal density (e.g., Ojha et al. 2020; Belleguic et al. 2005; Goosens et al. 2017; Plesa et al 2022)). This may also explain the observed offset between COM and COF. I think that the results of the present work tend to rule out those models that assume that there is no dichotomy in crustal thickness. In these scenarios, the difference between COF and COM is attributed to differences in crustal density, and in models with almost constant crustal thickness, a temperature difference (or a difference in the degree of melting) between the hemispheres is more difficult to explain, as the insulation of the crusts is similar.

In addition to the general comments, I have some specific questions/comments about the method and the discussion of the results

1. Line 90: Some metrics should be provided to prove that the additional estimated parameters are not just absorbing unmodeled errors or residual noise. Does the addition of these time-variable gravity coefficients ameliorate the fit quality or the quality of orbit reconstruction?

2. Line 101: Since the atmospheric calibration is a key step in the methodology proposed in this work, it would be important to quantify the effect and relevance of atmospheric variability and model variability. Especially when computing the direct effect of the gravity field due to the atmosphere: what is the effect of model variability? Do different (but reasonable) inputs to the model produce appreciably different gravity contributions? Do different models produce compatible results?

3. Additionally, in the manuscript it is mentioned that MCD is used for computing surface pressure fields (thus the indirect effect of atmospheric loading), while DTM-Mars is used to compute the direct effects and drag (not written explicitly, but it's mentioned that the same method of Genova et al., 2015 was used). Are these two models consistent? Can they be safely used independently to compute different contributions of the atmosphere to the gravity field?

4. Inversion procedure: Do you solve for degree 2 time-variable gravity terms? From the text it seems you do, but the results are never discussed, which is quite puzzling to me. The inversion solves for shear modulus variations at degree 1,2,3, so I don't understand why observations of degree-2 time variable gravity are not used. I understand the argument that degree-3 is enhanced through mode coupling; however, the model should be able to fit degree-2 terms simultaneously. There is a mention that the degree-2 terms should not be much influenced by lateral variations, however it would like to see that. Additionally, the supplementary information shows the posterior distribution of the observations. I understand the argument for not using C30 as an observation (i.e., it's not mainly related to mantle elastic properties), however the best-fit interior model should satisfy these constraints. Especially because the measured C30 values have been already calibrated for atmospheric contributions, so they should naturally be consistent with the other observations that have been used for inversion.
How do the authors explain this apparent inconsistency? This, from my understanding, cannot be justified by saying that these encompass unknown atmospheric effects, because the very same unknown atmospheric effects would change the other C_lm.

5. Additionally: the methodology relies on the use of externally-computed loading Love numbers:
a) how were these computed, based on which interior structure?
b) does the best-fit model reproduce the input loading Love numbers? These depend quite heavily on the viscosity structure of the mantle. The viscosity on the other hand depends strongly on the partial melt fraction. Thus, it would be an important self-consistency check.

6. line 144-148: "These amplitudes represent averages over the entire mantle depth range. When the mantle is subdivided into multiple layers, we find that the trade-off between the amplitude of heterogeneities in separate layers precludes statistically significant constraints on shear modulus structure in any layer."
Does that mean that a vertical variation in shear modulus is not required but possible? Are the data 'good' enough to conclude this (see points above)? Furthermore, see also my comment above that a homogenous distribution of melt down to the CMB is unlikely.
A question in the same direction: Fig 2 does not show a variation in shear modulus e.g. underneath Tharsis as suggested by dynamic models. Would you be able to see that in the data if existent?

7. The whole discussion about the offset in COM-COF is not clear:
Fig.3: The effect of topography has been accounted for resulting in a remaining offset of 0 – 1.82 km. Does this topography correction only account for topography or also crust thickness variation in case of Airy compensation (which crustal density is assumed)? Where is the uncertainty in the COM-COF offset (0-1.82km) coming from? Please provide the values of the offset from literature and the value for the correction.
In Eq. 13 rMoho is constant. However, with the assumed crust thickness variation rMoho varies between southern and northern hemisphere. Which value has been chosen and how large is the introduced error?
If only the topography is considered for the COM-COF offset without crust thickness variation, what are the results when crust thickness variations are considered? The contribution from the mantle is likely be smaller – possibly even not needed. The red shaded area will be narrower and/or shifted?

Equation 14 to calculate the density variation: Mars mantle composition has a higher iron content; this might change the assumed parameters. Furthermore, how realistic is it to apply this equation for the entire mantle, in particular considering that there is likely a density crossover between mantle material and melt at a depth of about 600-700 km.

8. Fig. 3 caption: "we infer a partial melt anomaly of >2% by volume as well as up to 60% pyroxene depletion over the Southern highlands mantle." Why 60% pyroxene depletion? On the figure it looks like up to 75% pyroxene.

9. Line 561: The authors simply assume a reference value for the shear moduls of the mantle, crust and core. Can a variation of these reference values influence the results?

10. Line 569-570 "We do not consider lateral changes in density (i.e., which may violate constraints from static gravity measurements.."
The authors predict a variation in depletion which is necessarily attributed to density variation in the mantle. It looks like that they violate their own assumption.

11. Line 217-222 "For example, if upwelling below the Southern highlands occurred after the initial crystallization of Mars's primordial crust, the resulting thermal anomaly could have raised parts of the solid lower crust above the Curie temperature [14]. This heating, in turn, would have allowed affected crust to acquire magnetization from Mars's ancient dynamo between 4.5 and 4.1 Gya [13, 46]."
Not sure that I understand this argumentation. The higher heat flow in the southern hemisphere reduces the thickness of the remanent magnetized layer here, but we see a stronger field in the southern hemisphere. What do the authors want to explain here?

Minor comments:
12. Line 68: The definition of resolution of the gravity field. I believe that here you are assuming that you need to resolve an entire wavelength, rather than half, as used by other authors. Worth mentioning.
13. Line 77: This depends on the wavelength. Long-wavelength static gravity, exactly like tides, is more sensitive to the deep interior. Rephrase this sentence. As it is written it's incorrect.
14. Table 1: Although mentioned in the methods, it is absolutely unclear from the main text what is the frequency of the periodic gravity signals that are estimated.
15. Line 603: The supplementary information discusses how the likelihood function is designed. In the discussion a covariance of the model is introduced but not defined. How is this computed?

(Remarks on code availability)

Referee #2

(Remarks to the Author)
In this study, A. Berne and collaborators investigated potential lateral heterogeneity in Mars's internal structure by analyzing its gravitational response to seasonal tidal interactions with the Sun. For that purpose, they analyzed radio tracking data from multiple Martian orbiters (Mars Global Surveyor, Mars Odyssey, Mars Reconnaissance Orbiter), over a total of 16 years. The present study extends previous analyses evaluating the temporal evolution of the zonal terms at degrees 2 and 3 (Genova et al. 2016, Konopliv et al. 2016) by assessing the non-zonal terms.
However, without sufficient justification, they did not assess the contribution of the seasonal tidal response to the zonal term and excluded non-zonal degree 2 terms from their analysis. Furthermore, they focused solely on annual (seasonal) temporal variations, while acknowledging that the degree 3 signal is stronger at diurnal frequencies. Finally, it is not entirely clear how the gravity signals of atmospheric forcing and the CO2 cycle load are modeled and taken into account in the analysis. These various elements cast doubt on their analysis and conclusion regarding the signatures of internal heterogeneity in the seasonal tidal response. Further justification and testing are needed to support their analysis and conclusion.

As shown by previous studies as well as on Fig. 1 in the Supplementary Information, the zonal term 30 in the time-varying gravity field is much larger than the non-zonal term, by at least one order of magnitude. The time variations of the zonal term 30 over the 16 years covered by MGS, ODY, MRO can be explained at first order by the polar ice cap mass exchanges. The observed 30 gravity signal include also the signatures due to the mantle response to the surface load as well as the 30 component of the seasonal tidal response. It seems to me that their proposed solution would be more robust if they could also reproduce the 30 zonal term and the different contributions (atmospheric/polar ice cap load, mantle response to the surface load, and tidal response). Based on Figure 1, the predicted impact of the atmosphere appears substantially larger than the effect of the seasonal tidal response. Any error in atmospheric correction can lead to a significant change in the relative contribution of the seasonal tidal response. I also find the data presentation problematic, as it compares observed data after atmospheric correction to the seasonal tidal response. It would be more relevant to graphically represent the total amplitude inferred from the observation and the contribution of each component, in order to explain the overall signal, including both observation and modeling uncertainties. I did not find any information regarding the assumed value for the load Love number, k2' and k3'. The authors only mention "Mars's loading love numbers k'l consistent with theoritical predictions (Wagner et al 2024), without specifying the exact values used. A range of possible values should be considered. The atmospheric component is estimated by the Mars Climate Database, based on the LMD GCM simulations. A discussion regarding the accuracy of the GCM prediction (and possibly comparison with other Mars GCMs) should be provided, as it is a critical aspect of the study. Any error in the atmospheric component can significantly alter the estimated amplitude of the tidal seasonal response.

The assumption of a constant load Love number, not taking into account the effect of lateral heterogeneity (l. 506-509), should also be further justified, and some tests using the LOV3D code should be carried out to support this assumption. Overall, a more careful quantification and discussion of the different components contributing to the observed time-varying signal should be done, in order to make the interpretation in terms of seasonal tidal response more robust.

The lateral variations in the mantle's effective shear modulus (> 40%) required to explain the seasonal tidal response at degree 3 appears very large. While heterogeneities of melt distribution between the two hemispheres appear a reasonable explanation, the minimum inferred melt fraction of 2%, considered as their favored explanation (l. 176-179), seems rather low. According to Schmeling (1985), for example, except in very specific melt configurations, it is difficult to reduce the shear modulus to this extent with only 2%. The inferred shear modulus variations instead indicate larger variations in melt fraction between the two hemispheres, which is more difficult to explain. The authors compared their inferred values to typical values of the melt content expected in the asthenosphere beneath Earth's mid-oceanic ridges to justify the typical range inferred in their study. But it must be kept in mind that, in the terrestrial context, this melt fraction is achieved very locally in specific geodynamic contexts, whereas here, they need such high values on a global scale in the mantle, and not only in the asthenosphere, to explain the complex 3D tidal response. A more careful discussion of the link between the inferred shear modulus variations, the melt distribution and the geodynamic context is needed.

Overall, I find this study very interesting and promising; however, the study lacks modeling details and sensitivity tests to concretely demonstrate the contribution of the seasonal tidal response to the observed time-varying, degree-three gravity field. The interpretation in term of melt variations between the two hemispheres also required further testing and discussions.

(Remarks on code availability)

Version 1:

Reviewer comments:

Referee #1

(Remarks to the Author)
The authors have provided a thorough and satisfactory revision, particularly regarding their interpretation of the shear module’s asymmetry, the change to the title, and the more detailed description of the methodology. Before publishing, however, I would suggest taking the following point into consideration:

The authors made a thorough investigation of how atmospheric model uncertainties affect the results. However, I find it limiting that these uncertainties are not propagated onto the calibrated ΔC/S_lm coefficients. While I appreciate the addition of the posterior distribution plots of shear modulus variation as a function of the atmospheric model adopted, the uncertainties reported in the manuscript (e.g., Figure 2 and Table 1) may be misleading to non-specialist readers. I suggest that the author explicitly state in the captions of Figure 2 and Table 1 that the uncertainties on the solid-body time-varying gravity coefficients do not account for uncertainty in the atmospheric model used to derive them.

(Remarks on code availability)
The provided code (Love3D_multi) does not allow readers to readily reproduce the results presented in this manuscript. The repository should include a worked example enabling reproduction of the key results. Since providing a full inverse Bayesian pipeline would be impractical, I recommend the authors include a script/example that takes the inferred interior structure (base structure + shear modulus variations) as input and demonstrates that it correctly reproduces the observed ΔC/S_lm values, within uncertainty.

Referee #2

(Remarks to the Author)
The authors provided a detailed response to the various points raised by reviewers regarding the first submitted version. The various additional tests carried out and included as Supplementary Information make the results more reliable and the interpretation of the shear modulus asymmetries deduced in terms of temperature variations and iron content is now more relevant. The implications for the thermal evolution of the Martian mantle, past volcanism and future measurements are also better argued. I have no further comments and I thank the authors for their detailed and thoughtful response and the careful revision of their manuscript, which is now suitable for publication in Nature.

(Remarks on code availability)

Open Access This Peer Review File is licensed under a Creative Commons Attribution 4.0 International License, which permits use, sharing, adaptation, distribution and reproduction in any medium or format, as long as you give appropriate credit to the original author(s) and the source, provide a link to the Creative Commons license, and indicate if changes were made.
In cases where reviewers are anonymous, credit should be given to 'Anonymous Referee' and the source.
The images or other third party material in this Peer Review File are included in the article's Creative Commons license, unless indicated otherwise in a credit line to the material. If material is not included in the article's Creative Commons license and your intended use is not permitted by statutory regulation or exceeds the permitted use, you will need to obtain permission directly from the copyright holder.
To view a copy of this license, visit https://creativecommons.org/licenses/by/4.0/

Referee Comments and Responses for:

Tidal Tomography Reveals a Thermal Anomaly Beneath Mars's Crustal Dichotomy

May 17, 2026

Referee #1

> *This paper analyzes non-zonal 3-degree components of Mars' time-dependent gravity field from Mars Global Surveyor, Mars Odyssey, and Mars Reconnaissance Orbiter. The authors show that, compared to spherically symmetric models, these components deviate by 200%, suggesting an asymmetric distribution of the shear modulus. This variation seems to be consistent with the north-south pattern of Mars' crustal dichotomy. Based on these values, the authors show that the mantle beneath the southern hemisphere has a melt fraction of more than 2%.*
>
> *The finding of an asymmetrical distribution of the shear modulus, which aligns with crustal dichotomy, is important because it confirms that the crustal dichotomy, which likely formed very early in evolution, continues to influence the thermochemical evolution of Mars to this day. This has already been postulated in several studies, but has not yet been confirmed from data: The thicker crust of the southern hemisphere with a higher concentration of heat sources and lower thermal conductivity than the mantle, results in a thinner lithosphere, higher temperatures, and more prolonged volcanic activity, in contrast to the thinner crust of the northern hemisphere (e.g., Schumacher and Breuer 2006; Plesa et al. 2018; Thiriet et al. 2018, Knapmeyer-Endrun et al. 2021; Plesa et al. 2022; Bonnet-Gibet et al. 2022).*

Response: Thank you for this clear and helpful summary of our work, and for your positive assessment of the results. As discussed in detail in our responses to your comments below, we have made substantial revisions to the manuscript in light of critiques (most notably regarding the interpretation of inferred shear modulus asymmetries). We hope that the revised manuscript addresses your concerns.

All line numbers, table numbers, and figure numbers referenced below correspond to the *revised* manuscript. Several sections have been substantially rewritten (e.g., *A Thermal Anomaly Beneath Mars's Southern Highlands*), so many line numbers indicated below will not align with those of the original submission.

However, the interpretation of the results in the present study differs in that the authors preferred model assumes that the mantle below the southern hemisphere has a melt fraction of more than 2% uniformly over the mantle depth range. In dynamic 3D models (Plesa et al., 2018; Plesa et al. 2022), temperatures below the southern hemisphere are indeed higher than underneath the northern hemisphere but present melting is found to be more localized, in regions of particularly thickened crust, such as below the Tharsis and Elysium region. The models with parameterized convection by Thiriet et al. and Bonnet-Gibet et al. indeed show melting or higher temperature beneath the entire southern hemisphere—however, it should be noted that these models only calculate mean profiles and cannot resolve the mantle convection pattern and the crustal thickness variations. What all these models—3D and parameterized—show, however, is partial melting below the lithosphere and not throughout the entire mantle down to the CMB, contrary to the authors' preferred model or at the CMB if a basal magma ocean does exist. Due to the pressure dependence of the melting temperature, this is not possible, or only under very specific conditions. To my knowledge, there is no model or scenario showing this. Also not the model by Bonnet-Gibet et al., which the authors cite as a possible scenario for the formation of the crustal thickness dichotomy. In addition, it should be noted that the model by Bonnet Gibet et al. is from its concept similar to the other models mentioned above, i.e. crust formation is preferential underneath thickened crust due to thermal insulation of the crust and its enrichment in radiogenic heat sources.

Response: Thank you for pointing this out. Although through-going melt could in principle explain the shear-modulus variations presented in Figure 2 of the manuscript, we agree with the reviewer that the studies of Gibet et al. (2022) and Thiriet et al. (2018) do not support through-going partial melt in the Martian mantle and that this interpretation is difficult to reconcile with the high pressures expected at depth in Mars's interior. We therefore agree that the original version of the manuscript overstates the case for widespread mantle melt and this should not be invoked as the preferred explanation for our tidal tomography result (i.e., Fig. 2 of the manuscript).

In seeking a better physical explanation for observed shear modulus asymmetries, we have re-examined the literature on the relationship between shear modulus and crystalline temperature in mantle rocks. In the original version of the manuscript, we had dismissed a purely thermal explanation on the grounds that the standard $d\mu/dT$ derived from experiments at seismic periods on olivine would require an implausibly large ($\gtrsim 1000$ K) temperature contrast to reproduce our inferred shear modulus differences. However, as highlighted by Jackson and Faul (2010), the effective shear modulus of olivine at long forcing periods (i.e., periods much longer than seismic timescales, such as the Martian annual period of 5.94×10^7 s) is substantially more sensitive to temperature than at seismic periods. This is because, at long forcing periods, anelastic relaxation mechanisms (e.g., grain-boundary sliding, diffusion creep, which are strongly temperature dependent; Faul and Jackson (2015)) contribute significantly to the effective shear modulus.

Based on the above, we have extracted the experimental data from Jackson and Faul (2010) shear modulus vs forcing timescale curves evaluated at different temperatures (specifically the 'background' Burgers model in Figure 1a of that work), fit each temperature curve with a power law function, and extended these fits to the Martian annual period (5.94×10^7 s). The resulting values are shown below in R.1

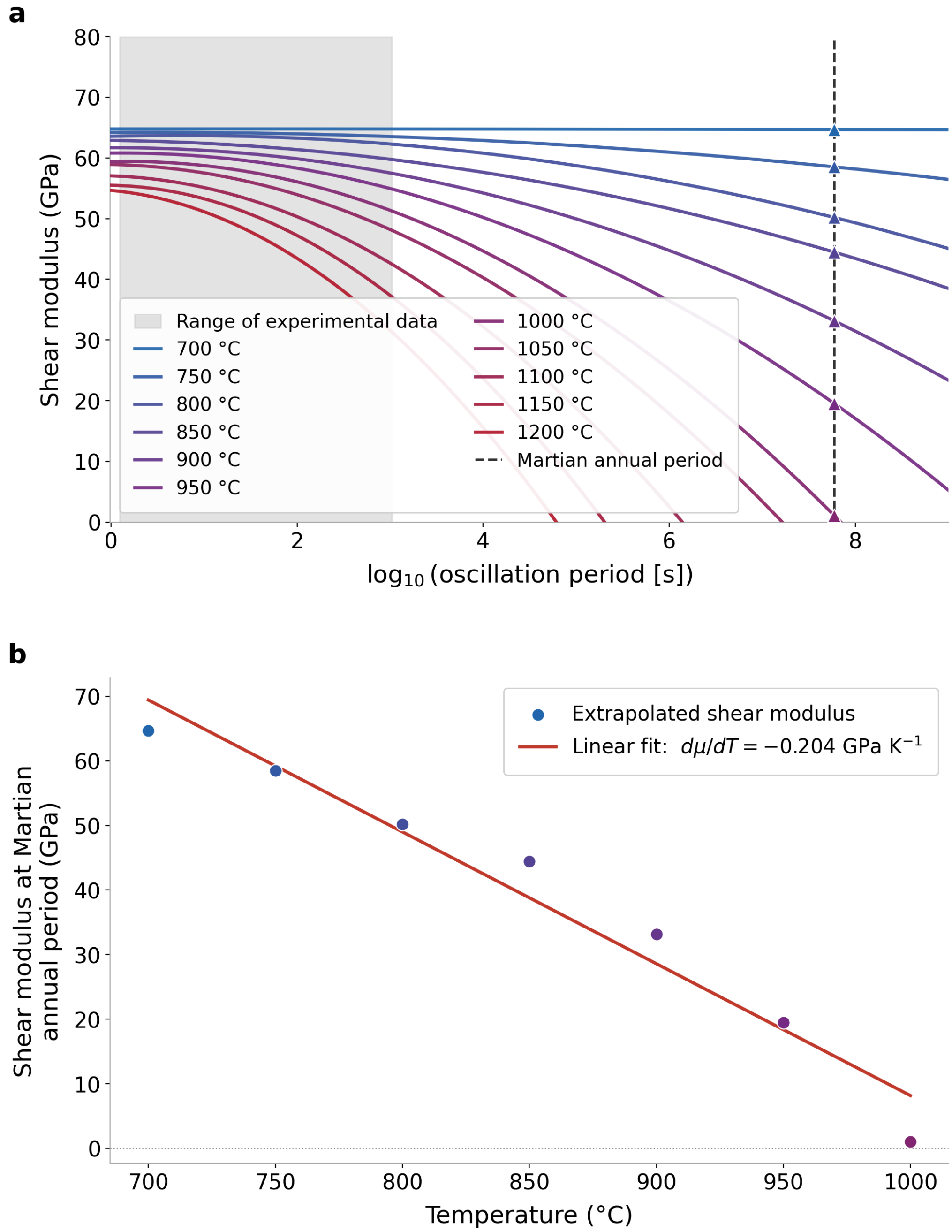

R.1: Sensitivity of olivine shear modulus to temperature at the Martian tidal period. (a) Shear modulus versus oscillation period for the background-only Burgers model of olivine rheology evaluated from experiments on crystals at temperatures between 700–1200°C (see Fig. 1a of Jackson and Faul (2010)). Colored curves are power-law fits extended beyond the range of experimental data (gray shading) to the Martian annual period (5.94×10^7 s; dashed vertical line). **(b)** Shear moduli evaluated at the annual period as a function of temperature. A linear trendline (red) yields $d\mu/dT \approx -0.204$ GPa K^{-1} which is used to recover hemispheric variations in temperature from shear modulus asymmetries (see Fig. 3 of the revised manuscript).

From these curves, we estimate an approximate (linear) transfer function between shear modulus and temperature change at the Martian annual period of:

$$\frac{d\mu}{dT} \approx -0.204 \text{ GPa K}^{-1}.$$

Which would require a temperature contrast of approximately:

$$\Delta T \sim \frac{\Delta\mu}{|d\mu/dT|} \approx 200\text{–}400 \text{ K}.$$

to explain our tidal tomography result (see updated Fig. 3 of the manuscript, also R. 2 below). We note that several anelastic laws have been published (e.g., Yamauchi and Takei 2016; McCarthy et al. 2011), but we have chosen that of Jackson and Faul 2010 as it has been used in several analogous applications for the Moon (Nimmo et al. 2012), Mars (Nimmo and Faul 2013), and the Earth (Dalton and Faul 2010; Lau and Faul 2019). Moreover, an explanation for mantle asymmetries based on a few hundred Kelvin temperature differences should be more consistent with existing literature on Mars's geodynamics (e.g., Gibet et al. (2022)) than an explanation that involves through-going partial melt. For example, a crystalline temperature anomaly is more consistent with the observations described in Figure 3 of Plesa et al. (2016) (panel f) as well as Sun and Tkalčić (2024) which describe differences in seismic wave attenuation between the Northern and Southern Highlands mantle (but do not find attenuation values which would indicate widespread partial melt in the Southern highlands).

In addition to this temperature anomaly, we infer a potential iron enrichment of up to 5% over the Southern highlands mantle (note, we now consider iron enrichment (Fayalite-Forsterite Mol fraction difference) instead of Pyroxene to Olivine depletion (as before) since the former is expected drive larger variations in density following melting and, by extension, COM–COF offset). As before, the inferred range of iron enrichment is derived by comparing the COM-COF offset to shear modulus asymmetries from our tidal tomography inversion. Notably, we would require COM-COF offsets that are approximately 50$\times$ observed values (Smith et al. (2001)) if we invoked iron enrichments in isolation to explain shear modulus anomalies (see R. 2 below, similar arguments could be made for other compositional variations e.g., water content), suggesting that inferred asymmetries are indeed largely thermal and not compositional.

Even if our results do not support a through-going partial melt anomaly, we note that the relatively elevated temperatures expected beneath the Southern highlands would make localized melting in this region (e.g., below the lithosphere) more likely than the Northern lowlands (as also noted by the reviewer). In principle, such a thermal state might be expected to enhance volcanism in the Southern hemisphere. This expectation is paradoxical in light of observations, which instead indicate more prominent young volcanic activity in the North, as discussed in lines 247–256 of the manuscript.

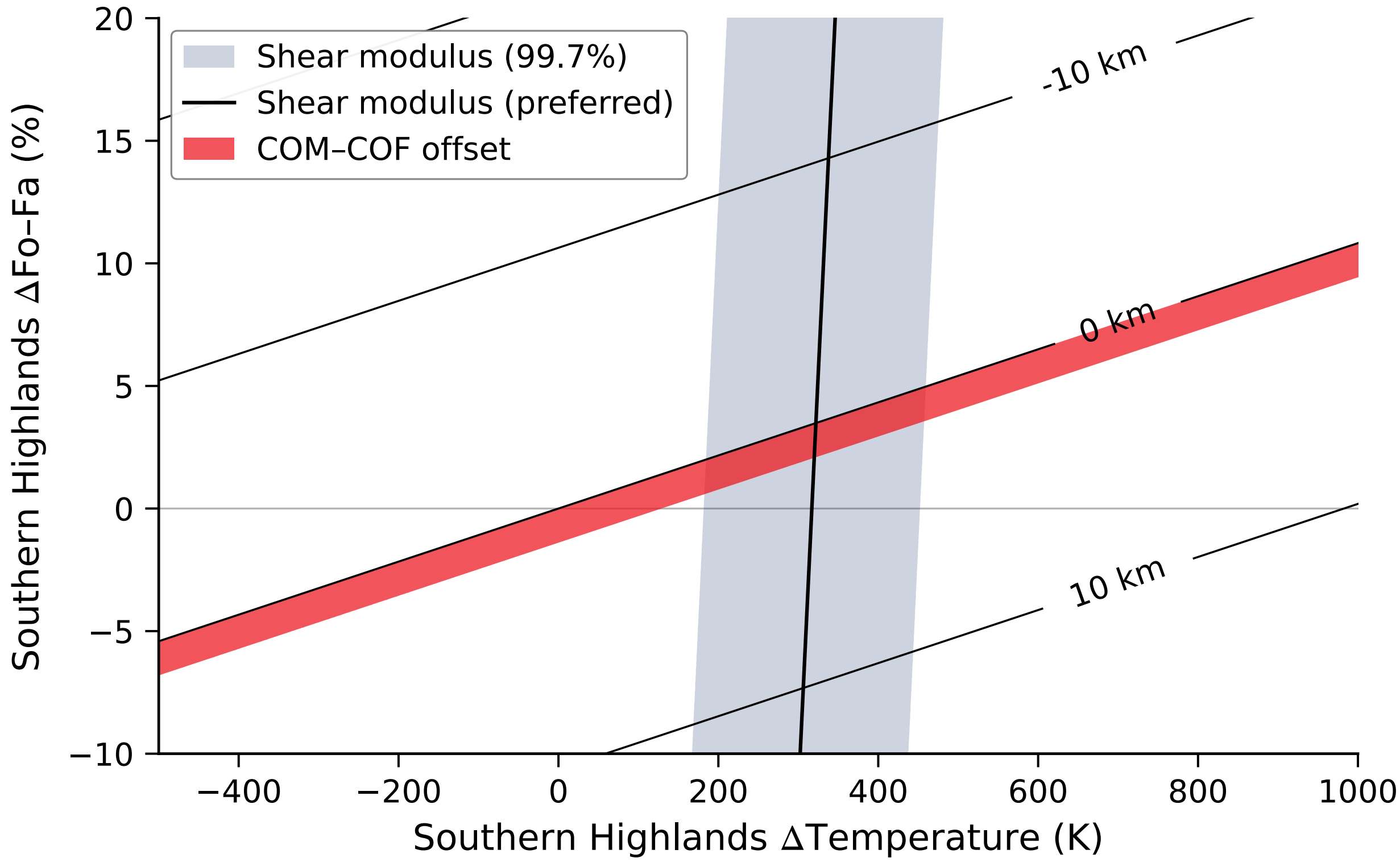

R.2: Relationship between temperature, composition, and inferred shear modulus variations in the Martian mantle. Shaded regions indicate the constrained ranges of the inferred Martian effective hemispheric shear modulus variation and the impact of mantle structure on the center-of-mass center-of-figure (COM–COF) offset as a function of Southern Highlands mantle temperature anomaly and fayalite–forsterite mol fraction contrast (Fa–Fo) (for details regarding calculations, see Methods). Values are projected onto a degree-1 pattern with poles centered at 45°N 138°W (Northern lowlands) and 45°S 42°E (Southern highlands). Black contours indicate COM-COF offsets at 10 km intervals. Since the COM–COF offset is sensitive to the structure of both the crust and mantle, our modelled 0–1.31 km range of values accounts for the possibility of (i) an anomalous COF that is fully attributable elevated topography over the Southern highlands (paired with Airy or Pratt compensation of this structure at depth) or an anomalous COM arising from a relatively dense crust over the Northern Lowlands (i.e., the 0 km bound) Smith et al. (2001), Wieczorek and Zuber (2004), and Wieczorek et al. (2022), (ii) an anomalous COM that is solely attributable to mantle density variations (i.e., the 1.31 km bound) and (iii) intermediate cases. The blue shaded area and thick solid black line respectively denote the 99.7% confidence bounds and preferred value for the overall shear modulus difference of $81 \pm 60\%$ inferred from gravity data in this work (see Fig. 2 of the revised text). By identifying overlapping portions of parameter space that satisfy both the 99.7% range of inferred shear modulus variation and COM–COF offset, we infer that hemispheric asymmetries can be explained by a temperature anomaly of 200–400 K and an iron enrichment of up to 5% in the Southern Highlands mantle.

Based on this comment, we have made the following changes:

- → We have changed the title of the paper to "Tidal Tomography Reveals a Thermal Anomaly Beneath Mars's Crustal Dichotomy"
- → We have heavily updated Figure 3 (which previously involved solving for the extent of Pyroxene depletion and extent of partial melting in the Southern highlands mantle using observed shear modulus and COM-COF offset). We have changed this to instead more directly infer iron content variations (i.e., ΔFa-Fo or change in fayalite to forsterite mol fraction) vs. temperature variations. Based on this Figure, we claim the existence of a temperature variation of approximately 200–400 K and ΔFa-Fo mol fraction variation of up to 5% over the Southern highlands mantle. Figure 3 also contains contour lines for the COM-COF offset to better support the argument that inferred compositional anomalies are relatively small.
- → We have heavily modified lines 183-202 to refer to the updated result (i.e., the inference of temperature variations of 200–400 K and compositional variations of up to 5%). In this modified paragraph, we discuss our logic for using the COM-COF offset to constrain the compositional component of inferred asymmetries.
- → We have changed the title of the section "A Partially Molten Mantle Beneath Mars's Southern Highlands" to "A Thermal Anomaly Beneath Mars's Southern Highlands".
- → We have edited Figure 4 to remove the depiction of through-going partial melt in Mars's mantle. Even so, we have kept the "Melt Ascent" in the crust in line with the prediction that there should be elevated amounts of partial melt in this region if this Southern highlands mantle is warmer than that in the north (though, we have changed the label associated with this feature within the Figure).
- → We have incorporated the Figure shown above (i.e., indicating the relationship between shear modulus and timescale for different temperature curves from Jackson and Faul (2010)) as Extended Data Fig. 5.
- → We have heavily modified the "Modeling Partial Melt and Pyroxene Depletion" (now titled "Modeling Temperature and Iron Content") to include discussion of our new method for calculating a transfer function between temperature and shear modulus variations (and other quantities). In this section we refer to the added Extended Data Fig. 5.
- → We have made numerous small edits throughout the manuscript, including to the abstract, the "Martian Volcanism and Future Measurements" section, and the Methods to accommodate the new result focused on 200–400 K temperature variations as an explanation for shear modulus variations.

> *In my opinion, the present study does not achieve what is suggested in some places in the manuscript, namely that it can also explain the origin of the dichotomy (different crust thicknesses in the southern and northern hemispheres). If the authors want to explain the origin of the dichotomy (see lines 60-65) with today's observations, they must explain what differences in internal structure can be expected for these different scenarios. The Bonnet-Gibet et al. model is preferred (but see the point above regarding the degree of uniform melting across the entire mantle depth range). Later, however, it is also stated that impact models cannot be ruled out as the cause of the dichotomy.*

Response: Thank you for your comment. We agree that the manuscript could be more direct

R.3: Possible mechanisms contributing to the Martian crustal dichotomy and their expected mantle signatures. The "Present-Day Structure" column indicates whether a given process is expected to produce a present-day thermal (**T**) and/or compositional (**C**) asymmetry.

**Process**	**Present-Day Structure**	**Notes**
Giant impact	**C**	Shock melting may generate a substantial compositional heterogeneity in the mantle (Marinova et al. (2008), Reese et al. (2011), Cheng et al. (2024), and Shaw (1970)), but is not expected (in isolation) to result in long-lived secular temperature anomalies of several hundred Kelvin in a given hemisphere (see e.g., Fig. S2 of Cheng et al. 2024).
Spontaneous degree-1 upwelling	**T**	Predicts a warmer mantle beneath the upwelling hemisphere; secondary compositional variations may arise through sporadic decompression melting Zhong and Zuber (2001) and Roberts and Zhong (2006).
Southern-crust insulation / radiogenic heating	**T**	Thick, heat-producing Southern Highlands crust suppresses heat loss and promotes long-lived mantle warming; secondary compositional differences may develop through sporadic decompression melting Plesa et al. (2018) and Gibet et al. (2022).

regarding the relationship between our core result and existing hypotheses for the formation of the crustal dichotomy. In particular, (as the reviewer mentions) it would be informative to clarify the relationship between our result and predictions for the structure of the present-day mantle from three hypotheses in the literature: a catastrophic impact, spontaneous upwelling/mantle convection over one hemisphere, and thermal insulation over the Southern highlands. Based on a literature search, we have added a short table (see above) detailing predictions from these hypotheses.

As summarized in R.3 above, the main distinction among different scenarios for the evolution of the crustal dichotomy is whether they are expected to produce a predominantly thermal or compositional present-day mantle asymmetry. Our preferred model indicates a 200–400 K temperature anomaly beneath the Southern Highlands, accompanied by, at most, only a modest compositional component (i.e., Fo-Fa mol fraction variation or iron enrichment between 0 and 5%; see Fig. 3 of the revised manuscript and R. 2). This pattern is, at first glance, more consistent with spontaneous degree-1 upwelling and/or thermal insulation by the thick Southern Highlands crust, both of which predict a primarily thermal mantle anomaly (Zhong and Zuber (2001), Roberts and Zhong (2006), Plesa et al. (2018), and Gibet et al. (2022)). By contrast, a giant impact alone may produce a large accompanying compositional heterogeneity in the mantle but is not expected to generate the inferred long-lived, thermal anomaly of several hundred Kelvin over a single hemisphere (e.g., Cheng et al. (2024), Fig. S2). However, we also expect that scenarios involving degree-1 upwelling

or thermal insulation would tend to *deplete* the Southern Highlands mantle in iron (opposite the enrichment we infer). A hybrid scenario could reconcile this discrepancy. For example, an early Northern hemisphere impact may initially (relatively) enrich the Southern Highlands mantle in iron (Reese et al. (2011)) and could also trigger the subsequent thermal insulation or degree-1 upwelling (Citron et al. (2018)) in the antipodal Southern hemisphere. This process would produce a dominantly thermal anomaly with only a small residual (<5% Fo-Fa) compositional component, consistent with our result.

Based on the reviewer's comment, we have made the following modifications:

→ We have heavily revised the second paragraph of the section "A Thermal Anomaly Beneath Mars's Southern Highlands" (lines 203-219) to more explicitly connect our tidal tomography result to hypotheses for the formation of the crustal dichotomy. In particular, the revised text now notes that an impact is not expected to produce a long-lived thermal anomaly over one hemisphere (in isolation), and that spontaneous degree-1 upwelling and Southern Highlands thermal insulation is expected to deplete (not enrich) this region in iron (opposite observations). We then state that our observations can be explained by a hybrid scenario (i.e., an impact over the Northern hemisphere followed by thermal insulation or degree-1 upwelling below the Southern Highlands mantle).

→ To more directly highlight the hypotheses related to thermal insulation by a thick Southern highlands crust on Mars, we have altered language in the introduction (lines 55 - 66) of the manuscript discussing this hypothesis (and citing Gibet et al. (2022) and Plesa et al. (2018)) and its relationship to predictions from hypotheses involving a hemisphere-scale impact and degree-1 convection.

I suspect that the manuscript's statement that non-zonal 3-degree components indicate a lower shear modulus underneath the southern hemisphere is most likely independent of the cause of the origin.

Response: That is correct. To be clear, the degree-3 time variations in Mars's gravity field that we describe require the presence of degree-1 variations in the shear modulus of the mantle. This shear modulus could be the result of either a compositional heterogeneity or a thermal heterogeneity (though, the somewhat small COM-COF offset limits potential compositional heterogeneities, see our discussion in response to the reviewer's first comment). This inference can help constrain models for the origin of the crustal dichotomy, but is independent of these models.

To make this point more clear, we have made the following changes:

→ We have incorporated a statement at the beginning of the second paragraph of the section "A Thermal Anomaly Beneath Mars's Southern Highlands" which clarifies that observed asymmetries in the Martian mantle provide independent constraints on the geodynamic processes that formed the crustal dichotomy.

→ We have altered lines 84 - 87 of the introduction of the manuscript to make it more clear that Mars's gravity field can be used to directly constrain deep-seated heterogeneities in Mars's interior.

At this point, it should also be emphasized that the observation that the southern hemisphere has a higher topography is usually explained by the fact that the crust beneath the southern hemisphere is about 25 km thicker than beneath the northern hemisphere (crustal thickness dichotomy). However, there are alternative models that do not assume a variation in crustal thickness but postulate a lateral variation in crustal density (e.g., Ojha et al. 2020; Belleguic et al. 2005; Goosens et al. 2017; Plesa et al 2022). This may also explain the observed offset between COM and COF. I think that the results of the present work tend to rule out those models that assume that there is no dichotomy in crustal thickness. In these scenarios, the difference between COF and COM is attributed to differences in crustal density, and in models with almost constant crustal thickness, a temperature difference (or a difference in the degree of melting) between the hemispheres is more difficult to explain, as the insulation of the crusts is similar.

Response: Thank you for the comment. We agree that the manuscript should more clearly acknowledge that the observed COM–COF offset does not uniquely require a hemispheric variation in crustal thickness, and may also be explained by a (approx. 200 kg m-3) lateral variation in crustal density (Kim et al. 2023; Wieczorek et al. 2022). The 0–1.31 km COM–COF range adopted in our revised manuscript (see Fig. 3) should encompass both end-member interpretations (for example, if the Southern highlands modifies the COF by 1.31 km relative to the COM, then mantle density variations should not contribute more than '0' km; a similar argument can be made if the elevated density of the Northern lowlands displaces the COM by 1.31 km relative to the COF). In this framework, if crustal topography/density variations do not displace either the COM or COF, then the 1.31 km COF-COM offset would be due fully to mantle density variations. This logic was not stated clearly in the original text. Therefore, in line with the reviewer's comment, we have made the following changes:

- → We have revised the first paragraph of the introduction to note that the Martian crustal dichotomy may be expressed either as an approximately 25 km hemispheric contrast in crustal thickness or, alternatively, as a lateral crustal-density contrast of order 200 kg m^{-3} (Wieczorek and Zuber (2004), Wieczorek et al. (2022), Goossens et al. (2017), Plesa et al. (2022), Belleguic et al. (2005), and Ojha et al. (2020)).
- → We have revised the caption of Fig. 3 to make explicit that the adopted COM–COF constraint range accommodates the endmember cases of this quantity being attributable solely to elevated topography over the Southern highlands, a lateral variation in crustal density, or mantle density variations.

Moreover, as the reviewer points out, although the distinction between a crustal thickness and a crustal density variation does not affect our core result (because the non-zonal degree-3 tidal response is only weakly sensitive to crustal structure; see Fig. 1b of the revised manuscript), it does affect how that result should be interpreted in the context of formation models for the crustal dichotomy. In particular, if the dichotomy reflects lateral crustal density variations rather than a thicker Southern Highlands crust, then thermal insulation becomes a less plausible explanation for the inferred present-day mantle temperature anomaly. In this case, our results would more uniquely favor degree-1 upwelling as the hypothesis for the formation of the crustal dichotomy. To make this point more clear, we have made the following additional change:

- → We have revised the final sentences of the second paragraph of the section "A Thermal

Anomaly Beneath Mars's Southern Highlands" (lines 216-219) to clarify that, if the crustal dichotomy reflects lateral density variations rather than a thicker Southern Highlands crust, our results would more uniquely favor hypotheses for degree-1 upwelling over the Southern highlands mantle.

> *In addition to the general comments, I have some specific questions/comments about the method and the discussion of the results.*

Response: Thank you. We have addressed these comments below.

> *1. Line 90: Some metrics should be provided to prove that the additional estimated parameters are not just absorbing unmodeled errors or residual noise. Does the addition of these time-variable gravity coefficients ameliorate the fit quality or the quality of orbit reconstruction?*

Response: Thank you for this comment. We have performed additional analysis of post-fit tracking data residuals and the value of the estimated empirical accelerations. We used independent data for this test, i.e. data from 2017 that were not used in the determination of the gravity field and associated parameters. We find that the RMS of fit for our test arcs improves only marginally ($< 1\%$) for some arcs (and for some, the RMS is marginally higher); see the example figures we include here showing changes in RMS with and without the inclusion of the order degree-3 time-varying gravity terms:

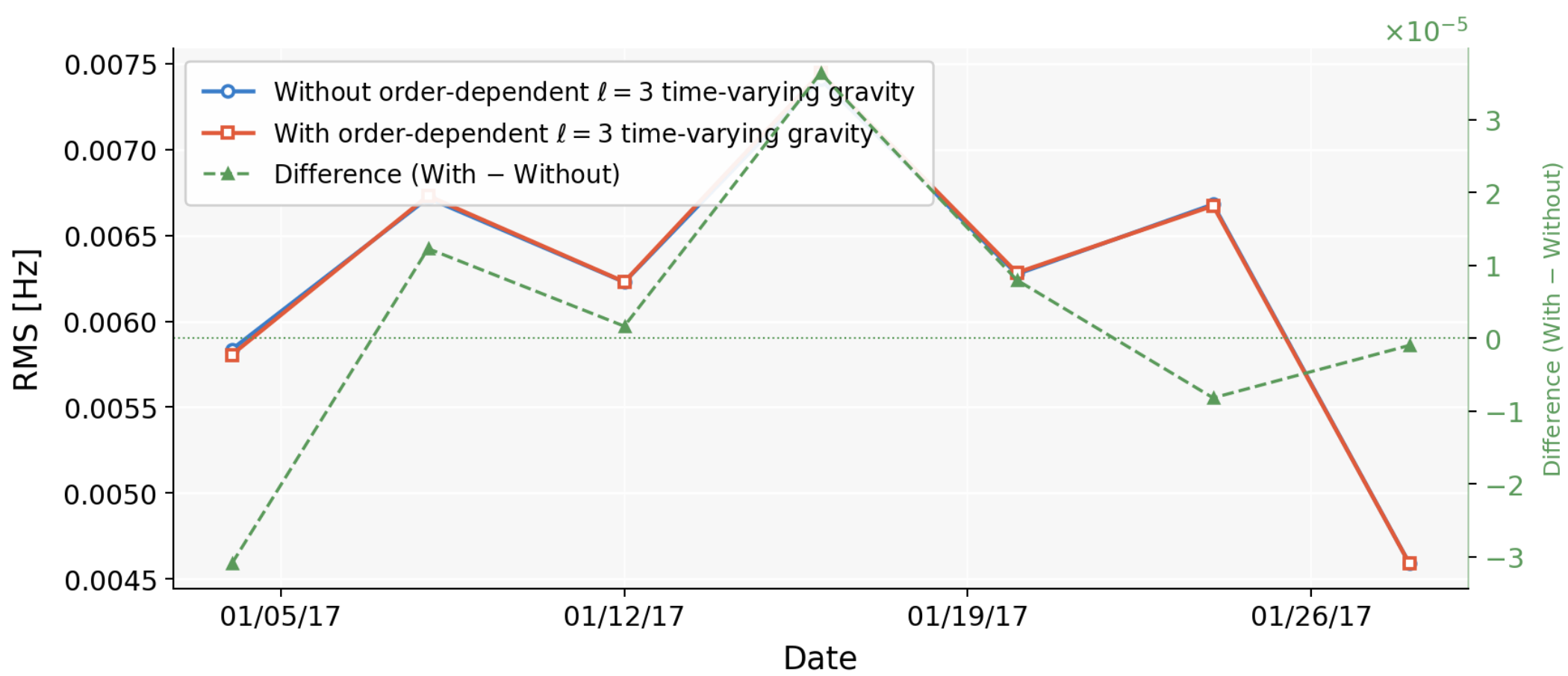

R.4: RMS of fit (Hz) for 2017 test arcs, comparing solutions without (blue) and with (red) inclusion of order-dependent $\ell = 3$ time-varying gravity terms. The green dashed line (right axis) shows the difference between the two solutions.

We note that this is expected. In general, it is true that adding more parameters to be estimated will improve the fit (which then in and of itself does not necessarily indicate whether the parameters are geophysically meaningful). These long-period time-varying effects can only be determined by stacking many years of data. We thus do not expect that every single arc has a marked improvement in fit. An improvement in fit will more readily come from improvements in the static gravity field

(which did not change substantially by adding the degree-3 time-varying components).

We also investigated the a posteriori value of the estimated empirical accelerations. As explained in the manuscript, these parameters are used to absorb mismodeling, most notably of diurnal effects and non-conservative forces. The periodic accelerations have a period equal to the spacecraft orbital period and they thus are not expected to influence the longer-period time-varying effects much, but nonetheless, their a posteriori value can be used to probe changes between parameterizations. We find slight improvements in the amplitude of the along-track accelerations when we include the additional gravity parameters (see the included Figure). However, the changes are again marginal. We find a more mixed result for the amplitude of the cross-track accelerations (see Figures below).

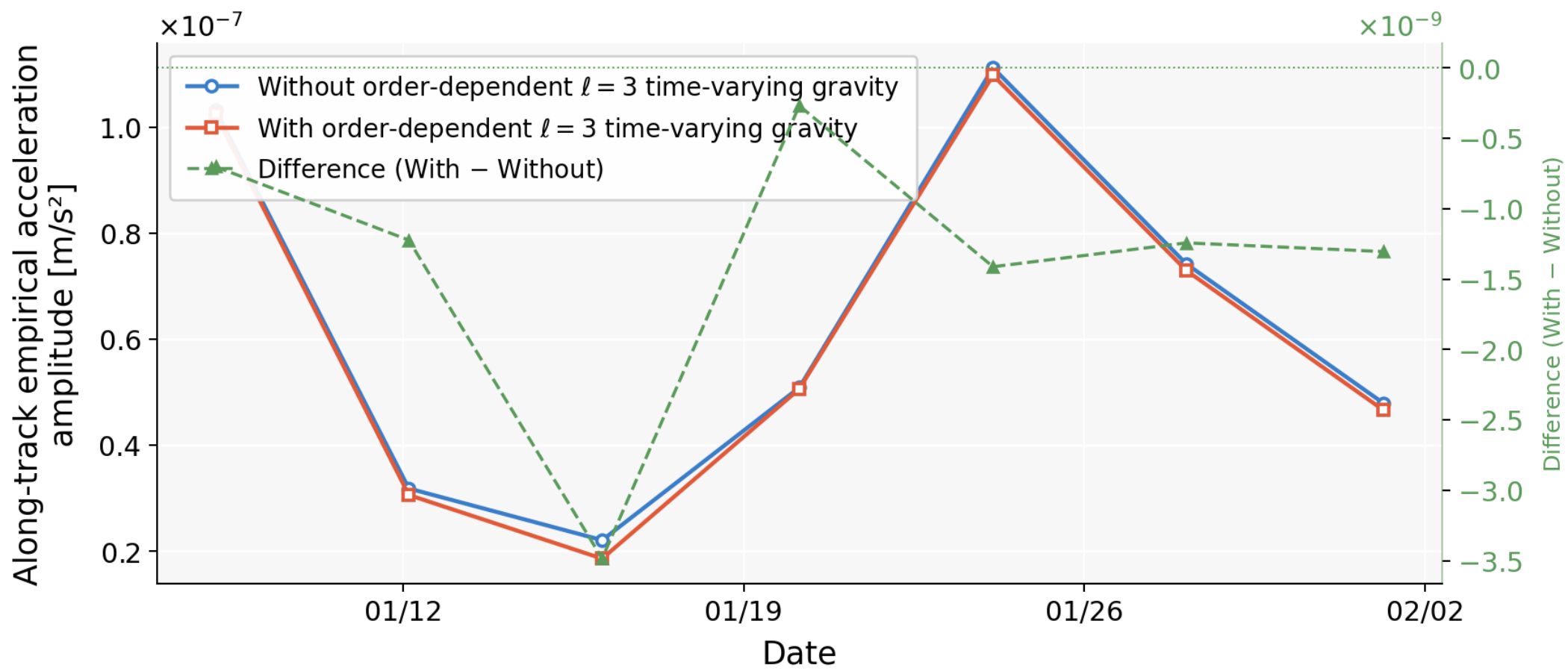

R.5: Along-track empirical acceleration amplitude for 2017 test arcs, comparing solutions without (blue) and with (red) inclusion of order-dependent $\ell = 3$ time-varying gravity terms. The green dashed line (right axis) shows the difference between the two solutions.

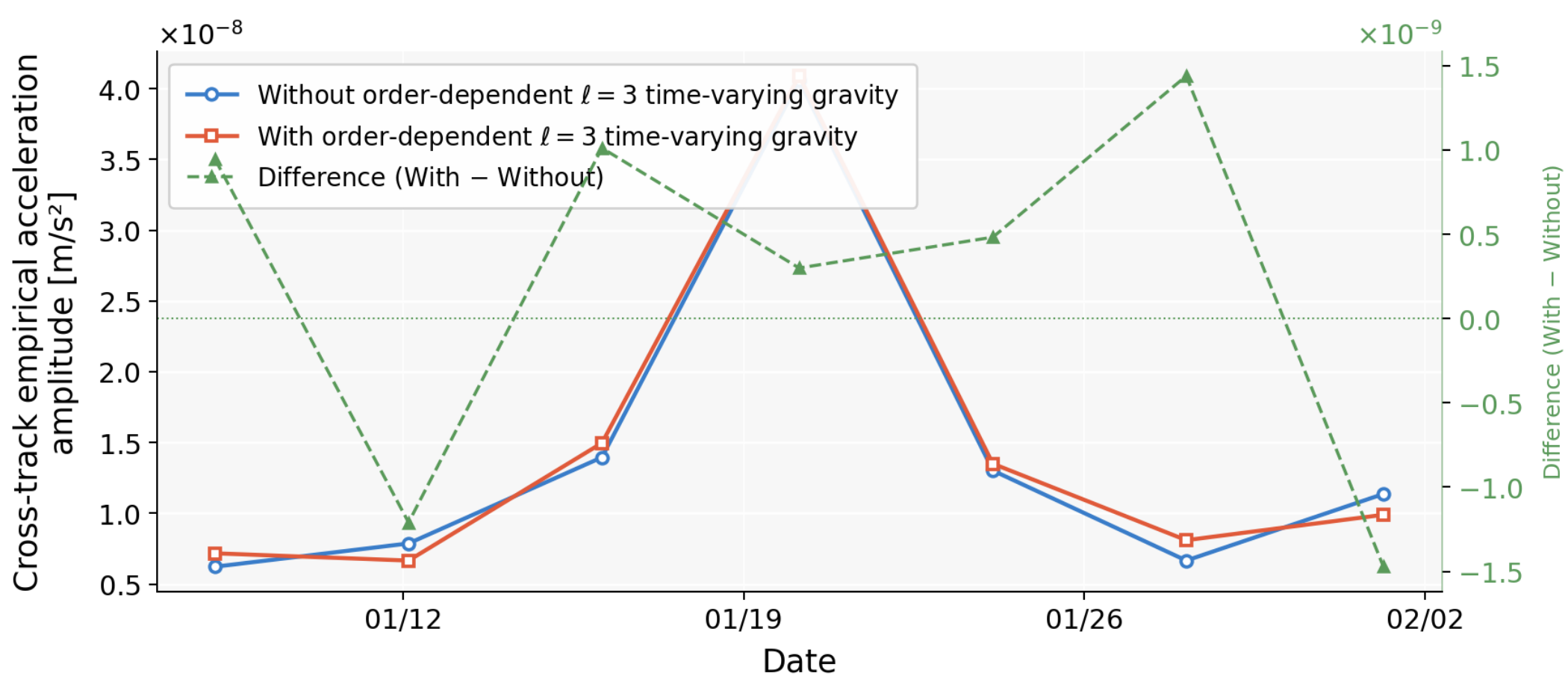

R.6: Cross-track empirical acceleration amplitude for 2017 test arcs, comparing solutions without (blue) and with (red) inclusion of order-dependent $\ell = 3$ time-varying gravity terms. The green dashed line (right axis) shows the difference between the two solutions.

Overall, the improvements and changes between analyses with different parameterizations that we find are marginal. We put more confidence in the fact that we find statistically and geophysically meaningful time-varying gravity coefficients which are robust. Even so, we agree with the reviewer that it is necessary to point out these nuances in order to provide a transparent characterization of our inversion procedure and give the reader appropriate context for evaluating the robustness of the estimated coefficients. As such, we have made the following changes:

→ We have included the RMS and empirical acceleration figures (R.4, R.5, and R.6 above) as Extended Data Fig. 6 in the revised manuscript.

→ We have added several lines of additional text to the "Gravity Field Inversion" section (lines 598-607) discussing the impact of incorporating the $\ell = 3$ time-variable gravity field coefficients on RMS and empirical acceleration amplitudes, and note that improvements in these parameters are marginal (though, this is expected). We refer to the newly added Extended Data Fig. 6 in the added text.

2. Line 101: Since the atmospheric calibration is a key step in the methodology proposed in this work, it would be important to quantify the effect and relevance of atmospheric variability and model variability. Especially when computing the direct effect of the gravity field due to the atmosphere: what is the effect of model variability? Do different (but reasonable) inputs to the model produce appreciably different gravity contributions? Do different models produce compatible results?

Response: Thank you for this important comment. We agree that the sensitivity of our results to atmospheric model choice warrants additional consideration. We address this below.

Our gravity field estimation follows the methodology of Genova et al. (2016), in which the contributions due to the atmosphere are estimated prior to solving for time-variable gravity coefficients and tidal parameters. Genova et al. (2016) derived these coefficients from surface pressure grids from the Mars Global Reference Atmospheric Model (Mars-GRAM 2010; Justh (2014)), at a 2-hour cadence. Genova et al. (2016) found that the key variables that impact predicted pressure field (and therefore the atmospheric gravity correction) include: (i) the solar flux (ii) the level of atmospheric dust opacity (background or dust storm conditions). Genova et al. (2016) assessed the sensitivity of the gravity solution to (i) and (ii) and find that that whereas the incident solar flux has a monotonic impact on gravity coefficients, dust opacity tends to amplify the impact of changes in incident solar flux on Mars's time-variable gravity coefficients. To verify, we have re-computed these variations for two endmember cases for comparison to our nominal atmosphere (i.e., an average solar flux scenario, 'Avg EUV', $F_{10.7} \approx 130$ sfu): (1) a cold atmosphere case with minimum solar flux ($F_{10.7} \approx 70$ sfu or solar flux units where $1\ \text{sfu} = 10^{-22}$ W m^{-2} Hz^{-1}) combined with dust-storm conditions (column-integrated visible optical depth $\tau \gtrsim 5$ Madeleine et al. (2011)) or 'Cold min EUV dust', and (2) a warm atmosphere case with maximum solar flux ($F_{10.7} \approx 200$ sfu) combined with the same dust opacity or 'Warm max EUV dust' (models are extracted from Millour et al. (n.d.)). We find that variations in the retrieved $\Delta C_{3m}^{A,B}$ and $\Delta S_{3m}^{A,B}$ across these permutations are stable across multi-year timescales (see R.7 below) and are generally smaller than uncertainties presented in Table 1 and Extended Data Table 1 in the revised manuscript (see also R.8 below).

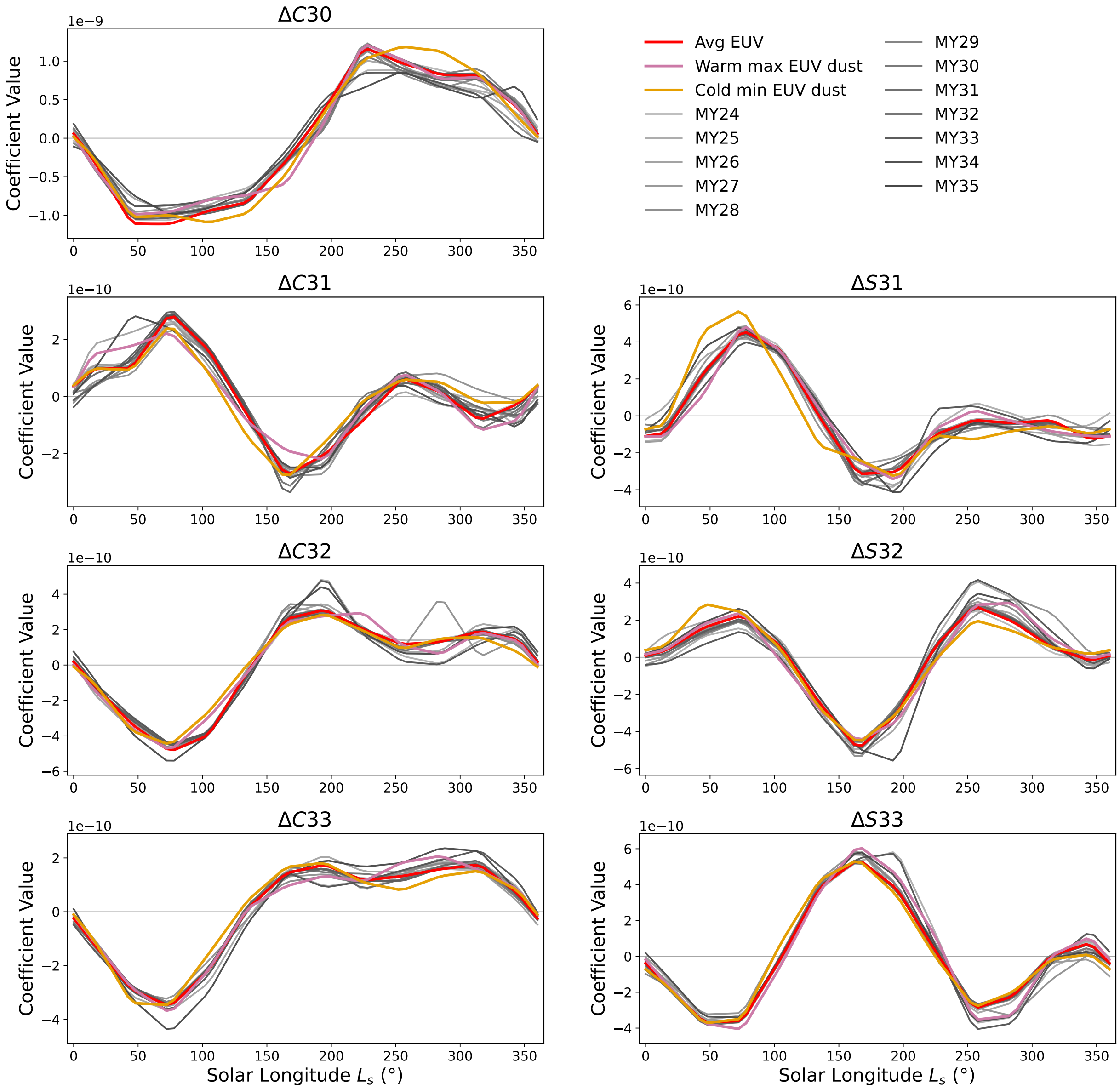

R.7: Seasonal evolution of degree-3 gravity coefficients across models for Mars's atmosphere. Curves show the 4π-normalized, demeaned seasonal time series of ΔC_{30}, ΔC_{31}, ΔS_{31}, ΔC_{32}, ΔS_{32}, ΔC_{33}, and ΔS_{33} as a function of solar longitude L_s. Colored curves denote the average-extreme ultraviolet (EUV) (red), warm/max-EUV with dust (pink), and cold/min-EUV with dust (orange) atmospheric scenarios, while gray curves show the individual realizations of the Avg EUV model from MY24 through MY35. Horizontal gray lines mark zero amplitude in each panel.

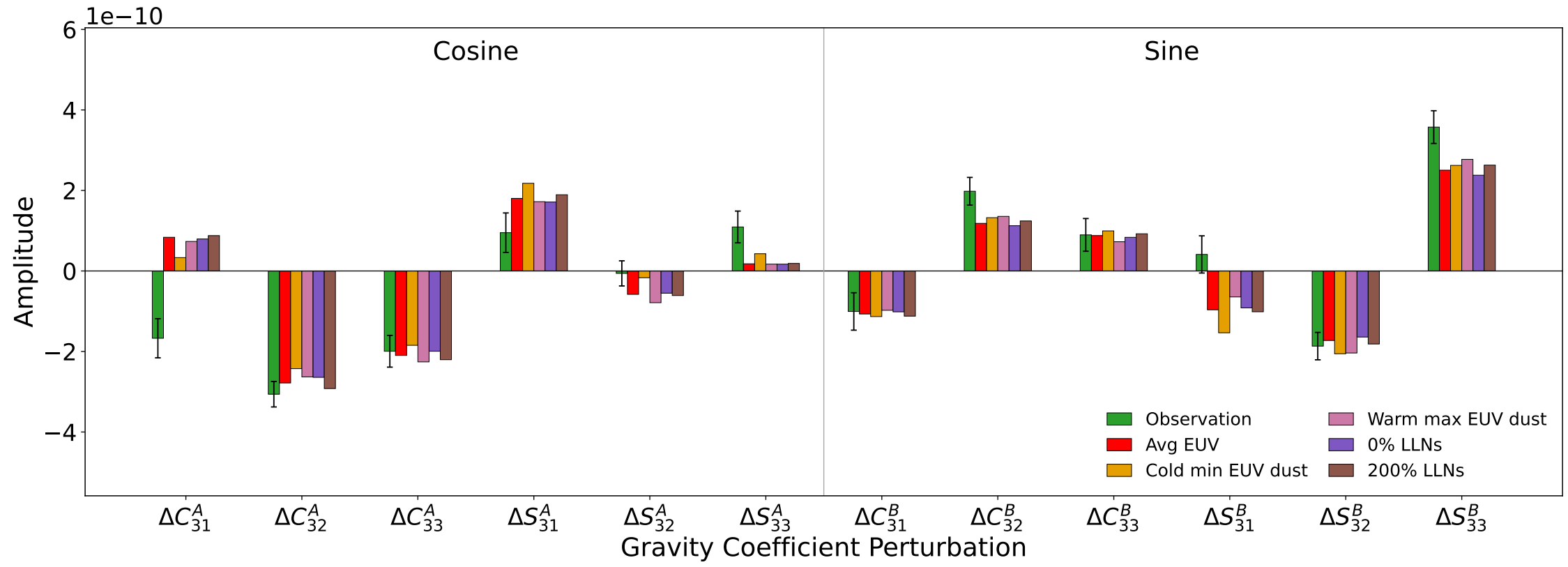

R.8: Sensitivity of the degree-3 gravity signals to atmospheric models and assumed loading Love numbers. Bars show the total observed seasonal degree-3 perturbations (green), the nominal atmospheric correction using the average-EUV scenario (red), atmospheric corrections for the cold/minimum-EUV/dust and warm/maximum-EUV/dust endmember scenarios (orange and pink), and cases with 0% and 200% the loading Love numbers k'_ℓ assigned to the nominal Average EUV atmosphere (purple and brown, see Equation 3 of the revised text). The left and right groups show the cosine and sine terms, respectively. Error bars on the observations denote 15× formal uncertainties.

As an additional check, we have directly computed the predicted impact of variations in (i) - (ii) above and re-run tidal tomography inversions adjusting the coefficients of the solid body tidal response (presented in Table 1 of the manuscript) to account for differences between each of these models, including at degree-2. The figure below shows the resulting posteriors for recovered degree-1 variations in shear modulus (analogous to panels a–c of Fig. 2 of the manuscript; see R.9 below):

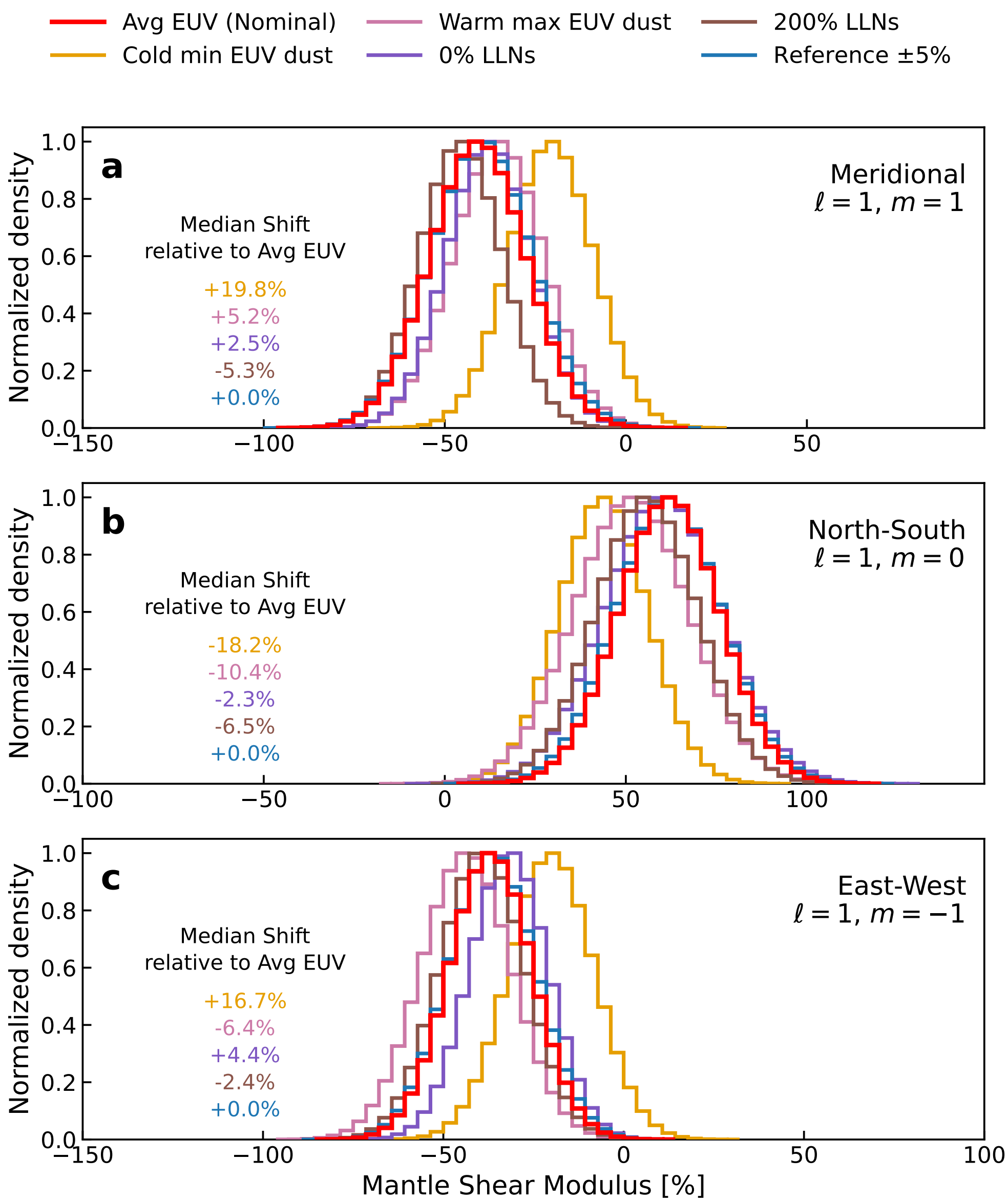

R.9: Sensitivity of tidal tomography results to atmospheric models, assumed loading Love numbers, and the choice of reference model. Posterior distributions of the degree-1 mantle shear-modulus coefficients for **(a)** the meridional $\ell = 1, m = 1$ term, **(b)** the north–south $\ell = 1, m = 0$ term, and **(c)** the east–west $\ell = 1, m = -1$ term for scenarios of the nominal atmospheric correction using the average-EUV scenario (red), atmospheric corrections for the cold/minimum-EUV/dust and warm/maximum-EUV/dust endmember scenarios (orange and pink), cases with 0% and 200% the loading Love numbers k'_ℓ assigned to the nominal Average EUV atmosphere (purple and brown, see Equation 3 of the revised text), and a case where the reference shear and bulk moduli of the crust and mantle are sampled from a Gaussian distribution centered about nominal values (see Table 1 of the revised manuscript) and a standard deviation equal to 5% of these values (light blue). Median shifts for each scenario relative to the average EUV case is shown to the left in each plot.

Based on the above, we find that the change in posteriors across atmosphere models is up to 20% of their median values (with largest shifts occurring for the 'Cold min EUV dust' case). These changes are certainly substantial, though, recovered posteriors indicate north-south patterns which still align with the crustal dichotomy (degree-1 order-0 variations remain significant at the 3-sigma level, and degree-1 order-1 variations remain significant at the 2-sigma level).

We agree with the reviewer that this sensitivity analysis should be incorporated into the manuscript. We have therefore made the following changes:

- → We have incorporated additional text in the Methodology section ('Gravity Field Inversion', lines 608-674) describing our procedure for correcting for Mars's atmosphere. In this added text (lines 654-674), we discuss the impact of different assumed atmospheric models on the time-variable gravity coefficients presented in Table 1 and Extended Data Table 1 as well as the results presented in Fig. 2 of the revised manuscript. In the added text, (line 669), we also note that different atmospheric models produce shifts of up to 20% in inferred shear modulus asymmetries.
- → We have included incorporated R.7, R.8, and R.9 above as Extended Data Figs. 7, 8, and 9.
- → We have added $1-\sigma$ uncertainties to red bars (i.e., for the atmosphere) in Fig. 1a based on inter-annual variations in the gravity field predicted by the Avg EUV model.

We also note that this response (including changes to the manuscript) overlaps substantially with comments raised by Referee #2 regarding the impact of the atmosphere on our inversion results.

> *3. Additionally, in the manuscript it is mentioned that MCD is used for computing surface pressure fields (thus the indirect effect of atmospheric loading), while DTM- Mars is used to compute the direct effects and drag (not written explicitly, but it's mentioned that the same method of Genova et al., 2015 was used). Are these two models consistent? Can they be safely used independently to compute different contributions of the atmosphere to the gravity field?*

Response: We thank the reviewer for raising this point regarding the consistency between the atmospheric models used at different altitude regimes. The use of different models reflects the current limitations in the observational coverage of the Martian atmosphere. In particular, significant gaps in measurements at intermediate and high altitudes prevent the development of a single model capable of consistently representing both the lower atmosphere (troposphere and lower thermosphere) and the upper thermosphere across all relevant spatial and temporal scales.

For this reason, general circulation models, such as the MCD or Mars-GRAM, are used to describe the lower atmosphere and to generate surface pressure fields. These fields are required to compute the gravity contribution associated with atmospheric mass redistribution. At higher altitudes, however, GCMs rely on simplified or theoretical thermospheric representations, which are limited by the scarcity of in situ data, particularly above 200 km, as discussed by Bruinsma and Lemoine (2002).

To accurately model the atmospheric density in the altitude range relevant for spacecraft dynamics, the Drag Temperature Model Mars is adopted. In our previous work, Genova et al. (2015), this model was further refined using MRO tracking data to improve the temporal variability of the main atmospheric constituents. Importantly, this refinement does not make the thermospheric model

inconsistent with the GCM-based pressure fields used at lower altitudes. DTM-Mars is a semi-empirical model of the upper atmosphere whose background temperature structure is derived from GCM-based representations of Martian atmospheric variability (Bruinsma and Lemoine (2002)). The refinement introduced by Genova et al. (2015) adjusts the relative abundances of the main atmospheric species in the thermosphere, under the assumption of diffusive equilibrium, to better match the radio-tracking constraints, but it does not alter the underlying large-scale thermodynamic structure. As a result, although the GCM and DTM-Mars apply to different altitude regimes, they share a common physical foundation and are internally consistent.

In any case, we agree with the reviewer that it is important to more clearly discuss the use of the two models in the manuscript. We have therefore made the following changes:

→ We have a paragraph in lines 634-641 discussing the use of GCMs and DTM-Mars, clarifying that GCMs generate surface pressure fields for the atmospheric loading correction and DTM-Mars models thermospheric density and drag. We note that this approach is consistent because the two models apply to different altitude regimes but draw from the same observations and underlying physical assumptions for the martian atmosphere.

> *4. Inversion procedure: Do you solve for degree 2 time-variable gravity terms? From the text it seems you do, but the results are never discussed, which is quite puzzling to me. The inversion solves for shear modulus variations at degree 1,2,3, so I don't understand why observations of degree-2 time variable gravity are not used. I understand the argument that degree-3 is enhanced through mode coupling; however, the model should be able to fit degree-2 terms simultaneously. There is a mention that the degree-2 terms should not be much influenced by lateral variations, however it would like to see that.*

Response: Thank you for this comment. Our gravity inversion procedure does solve for the order-dependent degree-2 terms alongside the degree-3 terms. Originally, we excluded all degree-2 terms (zonal and non-zonal) as constraints on the inversion based on the assumption that the impact of degree-1 (or degree-3) shear modulus variations on degree-2 gravity (i.e., in the presence of degree-2 forcing) is very small (see e.g., discussion in Park et al. (2025)). Changes in degree-2 response in the presence of degree-2 structure could also theoretically occur for Mars, though excluding degree-2 time-variable gravity would not allow for constraints on this potential structure.

As such, we agree with the reviewer that incorporating the degree-2 time varying gravity would make the inversion (and associated results) more complete and self-consistent. We have therefore re-run tidal tomography inversions for the manuscript including the degree-2 time-variable gravity field recovered from the gravity inversion step. We find that the inclusion of the degree-2 terms has minimal impact on our inversion results– this is implied by the fact that the posteriors for degree-2 terms, exhibit minimal variance across accepted models in Extended Data Figure 1 (see also the updated Extended Data Figures 2 and 3).

Based on this comment, we have made the following changes (note that these draw from the same underlying analysis as changes made in response to a similar comment by reviewer 2):

→ We have revised Figure 2 of the manuscript (as well as Extended data Figures 2 and 3) based on results from inversions which incorporate the degree-2 time variable gravity field terms as an additional constraints. We have also modified Extended data Figure 1 to incorporate

posterior distributions (and observations) for the degree-2 time-varying gravity field.

→ We have adjusted the third paragraph of our results section (i.e., 'Modeling Mars's Mantle Structure', lines 145-149) to incorporate discussion of the impact of incorporating degree-2 time variable gravity field as a constraint on inversions. We note in this added text that the lack of deviation between degree-2 terms and expectations for a spherically symmetric interior suggest minimal $\ell = 2$ structure in Mars's mantle.

→ In making changes associated with this comment, we encountered an error in the plot generation script in the original version of the manuscript which impacted the degree-2 order-0,2 histograms in Extended Data Figure 2, which has now been corrected.

> *Additionally, the supplementary information shows the posterior distribution of the observations. I understand the argument for not using C30 as an observation (i.e., it's not mainly related to mantle elastic properties), however the best-fit interior model should satisfy these constraints. Especially because the measured C30 values have been already calibrated for atmospheric contributions, so they should naturally be consistent with the other observations that have been used for inversion.How do the authors explain this apparent inconsistency? This, from my understanding, cannot be justified by saying that these encompass unknown atmospheric effects, because the very same unknown atmospheric effects would change the other C_{lm}.*

Response: Thank you for this comment. To clarify, the degree-3 (and degree-2) zonal time-variable gravity field terms were originally excluded due to the fact that the impact of condensation of the atmosphere into the poles over the seasonal period (i.e., the growth and sublimation of the polar caps) is expected to overshadow the solid-body response for these harmonics. We should also note that only the time-varying gravity field has been used to directly infer this seasonal polar mass change in past literature. For example, in Konopliv et al. (2016) and Genova et al. (2016), the zonal harmonics are used to directly constrain the ice mass variations (see e.g., Genova et al. (2016), which recover seasonal mass changes of $6.2\times10^{1}5$ kg and $8.4\times10^{1}5$ kg for the northern and southern caps, respectively), rather than the ice mass variation being used to predict corrections to the zonal gravity field. As such, these inferences are **not** used to calibrate ΔC_{30} and ΔC_{20} values in their work (or ours). This contrasts with modelled variation in the gaseous atmosphere, which has been more extensively validated against several independent observations (e.g., pressure logs, temperature measurements, occultation profiles; Montabone et al. (2006), Guo et al. (2009), and Millour et al. (n.d.)).

That being said, we agree with the reviewer that the manuscript should, for consistency, demonstrate that inversion results are compatible with past estimates of polar ice mass variation for Mars. We have therefore re-run our inversions incorporating the zonal terms as constraints (and run a parallel set of inversions which fully exclude zonal terms, but incorporates the non-zonal degree-2 gravity field as a constraint, see above comment). For these inversions, we assume that zonal terms comprise the sum of the solid body response and the impact of seasonal mass changes of 6.2×10^{15} kg and 8.4×10^{15} kg for the northern and southern caps, respectively. Posterior distributions for inversions that include and exclude zonal harmonics are shown below in R.10.

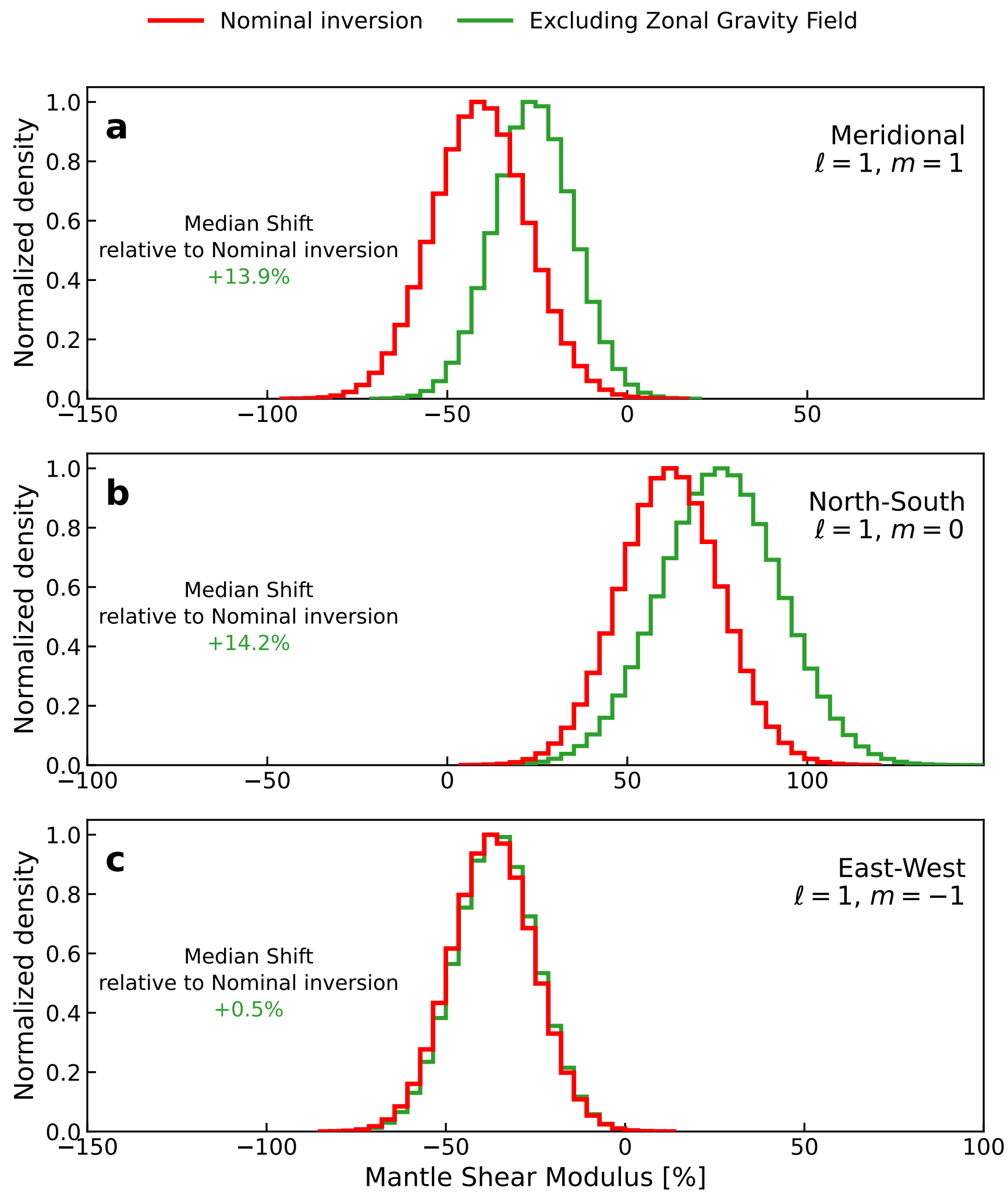

R.10: Comparison of tidal tomography results with an inversion that excludes zonal harmonics. Posterior distributions of mantle shear modulus asymmetries from an inversion that excludes zonal gravity harmonics in Table 1 and Extended Data Table 1 (green) including **(a)** the meridional $\ell = 1, m = 1$ term, **(b)** the north–south $\ell = 1, m = 0$ term, and **(c)** the east–west $\ell = 1, m = -1$ term. Posterior distributions are overlain with results from the nominal tidal tomography inversion presented in Fig. 2 or the revised manuscript (red). Median shifts relative to the nominal inversions are shown to the left in each panel.

As shown in R.10 above, the inclusion of zonal terms appears to shift the posterior distributions by up to 15%. We suspect that this shift is mainly attributable to the impact of the ΔC_{30}^{B} term, which shows a relatively wide posterior spread in Extended Data Fig. 1b of the updated manuscript (suggesting that the observational bounds on this parameter are influencing the acceptance of models in MCMCs).

Based on this discussion, we have made the following changes:

→ We have made several updates, including to Figs. 2 and 3, as well as Extended Data Figs

1,2, and 3 and Extended Data Tables 3 and 4, to incorporate the new results that use the time-variable zonal gravity field as constraints on tidal tomography inversions.

→ We have incorporated discussion of the inclusion of zonal harmonics as constraints on inversions (including the impact of this change on posterior distributions) in lines 723-727 of the methodology and added R.10 above as extended data Figure 10.

We note that this comment is closely related to comments raised by Referee #2 (specifically, those concerning the treatment of the zonal ΔC_{20} and ΔC_{30} observations), and so our response and the associated changes to the manuscript overlap substantially with our response to those comments.

> *5. Additionally: the methodology relies on the use of externally-computed loading Love numbers:*
> *a. how were these computed, based on which interior structure?*

Response: Thank you for this comment. We compute loading Love numbers k'_ℓ for a spherically symmetric, self-gravitating 1D elastic Mars using the semi-analytic method described in Wagner et al. 2024. The reference radial interior used for these calculations is the same as that used as the reference model for tidal tomography inversions (also shown below in R.11 for reference):

R.11: Assumed 1D reference interior model for Mars. Model is constrained by Mars's degree-2 tidal deformation, Moment of Inertia, Mean density, and seismic wave arrival times Stähler et al. (2021)

Radius (km)	Density (kg/m³)	Bulk Modulus (GPa)	Shear Modulus (GPa)
0 – 1830	6200	142.8	0.0
1830 – 3340	3600	130.2	79.5
3340 – 3390	3300	104.3	61.0

As an additional check (and to more generally probe the impact of our choice of loading Love number), we have conducted an additional set of inversions which assume k'_ℓ that are 200% and 0% (relative to baseline values noted above, the '0%' indicates a case with no deformational response to surface loads). Posteriors for these inversions are shown in R.9 above, and indicate that these changes induce shifts of, at most, ∼5% relative to the results of nominal inversions. In other words, our choice of loading Love number (i.e., associated with the uncertainty of the assumed 1D model, which is much smaller than 100%) has an effectively negligible effect on inversions results.

In any case, we agree with the reviewer that these methods and the impact of assumed k'_ℓ can be more clearly described in the manuscript. We have therefore made the following changes:

→ We have revised lines 619-622 in the methodology to describe the methods used to compute loading Love numbers, including mention of the semi-analytic approach described in Wagner et al. 2024. We mention of the assumed interior model in the added text.

→ We incorporate discussion of varying k'_ℓ on our inversion results in lines 629-633 of the methodology.

→ We incorporate results associated with varying k'_ℓ by 200% and 0% relative to baseline values into Extended Data Figure 9 of the revised manuscript.

b. does the best-fit model reproduce the input loading Love numbers? These depend quite heavily on the viscosity structure of the mantle. The viscosity on the other hand depends strongly on the partial melt fraction. Thus, it would be an important self-consistency check.

Response: We agree that it is useful to check consistency between our solution and input loading Love numbers. To that end, we have carried out finite element simulations of the degree-2 and degree-3 loading Love numbers for the assumed 1D reference model with imposed degree-1 mantle shear modulus variations corresponding to the maximum a-posteriori values from Figure 2 a-c of the revised text (i.e., North–South, meridional, and East–West patterns with peak-to-peak amplitudes (and 3-σ uncertainties) of 60 ± 40%, 42 ± 38%, and 36 ± 34% respectively) and replacing the 'effective shear modulus' with the elastic shear modulus. For these calculations, we use an in-house finite element model following the methodology of Berne et al. (2023a), Berne et al. (2023b), Berne et al. (2024), and Berne et al. (2026) which is bechmarked against Lov3D for tidal Love numbers. Our finite element method involves solving Poisson's Equation and the Equation of motion for a laterally heterogeneous planetary body in a non-rotating and non-translating reference frame (see e.g., Supplementary of Berne et al. (2023a) for details on the implementation of the numerical code). We subdivide numerical domains into 150,000 nodes. For input forcings, we apply degree-2 and degree-3 fields across each spherical harmonic order, and average the resultant responses per degree.

The results for the degree-2 and degree-3 loading Love number derived from numerical models and that assumed as inputs into calculations are shown in the Table below. Generally, we find good agreement (<1% differences) between our result and input loading Love numbers:

R.12: Comparison of assumed and predicted Love numbers. The absolute difference is |Predicted − Assumed|, and the relative difference is expressed as a percentage of the assumed value.

Quantity	Assumed	Predicted	Abs. difference	Rel. difference (%)
k'_3	-0.09109	-0.09113	4.0×10^{-5}	0.044%
k'_2	-0.14126	-0.14127	1.0×10^{-5}	0.0071%

We should note that, for these Love-number calculations, we ignore potential mode coupling effects. This is because mode-coupling between degree-2 forcing and degree-3 response induced by the imposed 3D shear-modulus heterogeneity is expected to be to be very small relative to the within-harmonic (same-degree) response. This expectation follows from the fact that mode-coupling contributions enter with an additional small factor $\mathcal{O}(\delta\mu/\mu_0)$ relative to the within-harmonic response, and for the loading problem the within-harmonic forcing at degrees 2 and 3 are of comparable magnitude for atmospheric loading (this is a key distinction from body tides, where the degree-3 forcing is roughly five orders of magnitude smaller than the degree-2 forcing — see Part 1 of our response to Reviewer #2's LOV3D comment for a fuller discussion). Any mode-coupling of a degree-2 surface load into a degree-3 response by 3D structure, or vice versa, is therefore overshadowed by the direct within-harmonic response of the load at the same harmonic.

Based on this discussion (and to better justify our assumed values for the loading Love numbers) we have made the following changes:

→ We have added text noting the adopted loading Love numbers ($k'_2 = -0.14126$ and $k'_3 = -0.09109$) in line 622.

→ We note in lines 625-629 that finite-element calculations for our maximum a-posteriori model reproduce loading Love numbers to within $< 1\%$ of their nominal values.

6. line 144-148: "These amplitudes represent averages over the entire mantle depth range. When the mantle is subdivided into multiple layers, we find that the trade-off between the amplitude of heterogeneities in separate layers precludes statistically significant constraints on shear modulus structure in any layer." Does that mean that a vertical variation in shear modulus is not required but possible? Are the data 'good' enough to conclude this (see points above)? Furthermore, see also my comment above that a homogenous distribution of melt down to the CMB is unlikely.

Response: Thank you for your comment. We are indeed unable to differentiate radial variations in shear modulus in Mars's mantle. This is because the sensitivity kernel of degree-3 gravity field variations for degree-1 variations vs. depth varies sufficiently gradually within Mars's mantle so as to produce a substantial amount of non-uniqueness between the amplitude of allowable asymmetries over a given depth. To provide a quantiative example, if the cumulative sensitivity of the degree-3 gravity field is 50% between the depths of 50 km and 1000 km, and the remaining 50% sensitivity occurs between 1000 km and 1500 km, and a 10% total mantle shear modulus is needed to explain observations, then we could place either a 20% shear modulus variation in the 50 - 1000 km depth range or a similar shear modulus variation in the 1000 - 1500 km region to explain observations. The fact that the crust is very thin and has a relatively low impact on degree-3 gravity means that even ± 100% shear modulus variations result in $< 1\%$ changes to Mars's degree-3 response (and so can be confidently rejected as the sole source of degree-1 variations in structure following our tidal tomography inversion, see e.g., Extended Data Figure 2), though this logic cannot be extended to large radial regions of the mantle.

As an additional check on the above logic, we have carried out an inversion for which we split the mantle into two layers (radius 1830 - 2520 km and 2520 - 3340 km) and attempt to recover shear modulus variations in either layer. The results is shown in a corner plot below. As expected, the inferred shear modulus in the upper and lower portions of the mantle are highly correlated (i.e., they non-uniquely trade off with each other).

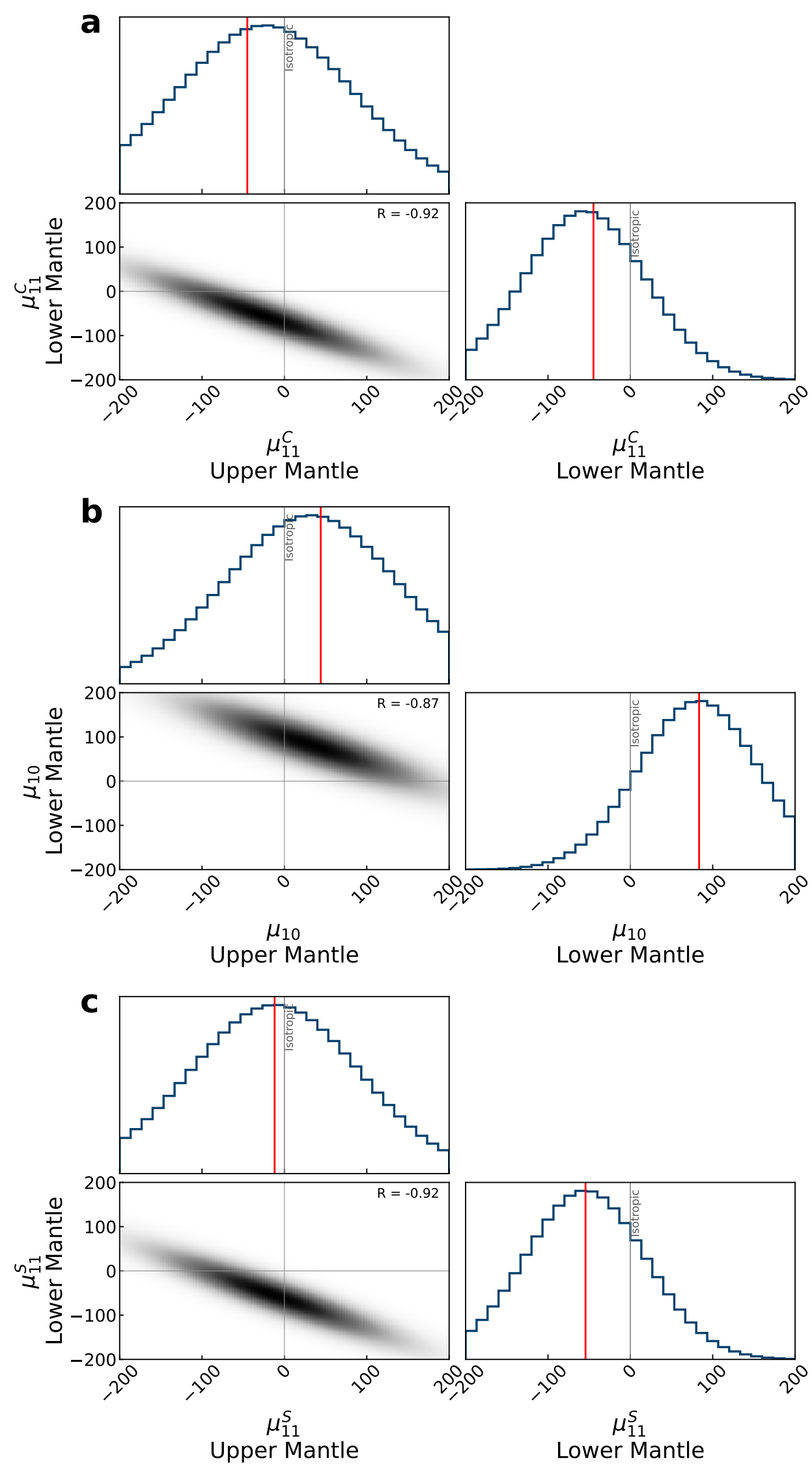

R.13: Corner plots for degree-1 shear-modulus perturbations in an inversion with the mantle subdivided into multiple layers. Degree-1 coefficients **(a)** μ_{11}^C, **(b)** μ_{10}, and **(c)** μ_{11}^S are derived for an inversion that subdivides the martian mantle into a lower portion (radius 1830 - 2520 km) and an upper portion (radius 2520 - 3340 km). Pearson correlation coefficients R are shown alongside 2D posterior distributions (values close to -1 indicate a substantially non-unique trade-off between recovered heterogeneity amplitudes). Red lines on 1D histograms indicate the median (50th percentile) of distributions. Gray vertical lines in 1D histograms indicate values expected for a laterally isotropic reference interior.

To more directly answer the reviewer's questions "Does that mean that a vertical variation in shear modulus is not required but possible? Are the data 'good' enough to conclude this (see points above)?"; yes and no. Vertical variations are certainly possible, but the degree-3 (or degree-2) gravity field is not sufficient to resolve variations at depth in a statistically rigorous way. In the future, higher-order variations in time-variable gravity (i.e., from spacecraft with more precise

gravity experiments) or independent measurements from e.g., magnetic induction or seismology could more precisely constrain the depth of inferred asymmetries, but this is beyond the scope of the current work.

We should also note that, in response to the reviewer's first comment, we have altered our inference of an average partial melt anomaly of 2% to an inference of a temperature variation of 200–400K, which we believe is much more consistent with independent observational constraints (e.g., differences in seismic quality factors between Southern highlands and Northern lowlands Sun and Tkalčić (2024)) as well as theoretical/modelling studies of heat flow in Mars's interior (e.g., Gibet et al. (2022) and Plesa et al. (2018)). Of course, the described degree-1 temperature anomaly/shear modulus variations that exists below the Southern highlands could be concentrated in a particular depth extent of the mantle (e.g., these could be concentrated below the lithosphere Gibet et al. (2022) or at the base of the mantle Samuel et al. (2023)), but this cannot be differentiated from a uniform (i.e., through-going) case based on our analysis.

Based on this discussion, we have made the following changes:

→ We have incorporated the Figure (i.e., the corner plot) mentioned in our response to this comment as an additional plot (Extended Data Figure 4).

→ We have revised the text near lines 158-165 to clarify that the recovered heterogeneity amplitudes represent depth-averaged variations across the mantle, and that this choice is made because the sensitivity of degree-3 time-variable gravity to the depth of 3D structure changes only gradually with depth. We also more explicitly state that subdividing the mantle into multiple layers prevents statistically significant constraints on the vertical distribution of the inferred asymmetries at any given region within the mantle. Finally, we note that our inversions still allow the possibility of more vertically localized structure in the interior (e.g., at the base of the lithosphere or near the CMB). We also refer to the added Extended Data Figure 4 in this revised text.

> *A question in the same direction: Fig 2 does not show a variation in shear modulus e.g. underneath Tharsis as suggested by dynamic models. Would you be able to see that in the data if existent?*

Response: Thank you. A few possible reasons for why a shear modulus anomaly is not detected below Tharsis from our tidal tomography inversion include:

(i) Variations are too small (in terms of total amplitude) relative to anomalies associated with the crustal dichotomy.

(ii) Structural anomalies associated with Tharsis are very shallow.

(iii) Anomalies associated with Tharsis are spatially confined in terms of lateral wavelength (i.e., associated with degrees larger than $\ell = 3$) in the mantle.

To clarify this logic, and in line with the reviewer's comment, we have made the following changes:

→ We have altered lines 175-181 to discuss reasons why we do not observe a shear modulus anomaly below Tharsis, including (i), (ii), and (iii) mentioned above.

7. The whole discussion about the offset in COM-COF is not clear: Fig.3: The effect of topography has been accounted for resulting in a remaining offset of 0 – 1.82 km. Does this topography correction only account for topography or also crust thickness variation in case of Airy compensation (which crustal density is assumed)? Where is the uncertainty in the COM-COF offset (0-1.82km) coming from? Please provide the values of the offset from literature and the value for the correction.

Response: Thank you. To be clear, we derive values for the COM-COF offset by extracting measurements for this quantity from Table 4 of Smith et al. (2001). The COM-COF offset is measured in three directions (i.e., Δx = 233 m, Δy = 1428 m, and Δz = 2986m) and has a total magnitude of 3.3 km. Smith et al. (2001) do not provide a formal uncertainty for COM-COF offsets shown in their Table 4, however, the uncertainty of similar parameters derived for the Moon by the same author (Smith et al. (1997)) are of order 10-20 m, which is a negligibly small portion (<1%) of the 0 - 1.31 km range of COM-COF offsets considered in Figure 3.

To derive the 1.31 km bound, we project the offset vector (which has a maximal magnitude in the 64.1N, 279.3W direction) onto the pole of the hemisphere whose 'North' pole is centered at the location of the maximum shear modulus value shown in Figure 2 (i.e., 45N, 138W). The magnitude of the resulting vector is 1.31 km (hence, the upper bound noted by the reviewer and used in Figure 3). We interpret this as an upper bound because, in the end-member case where crustal structure (i.e., topography, thickness variations, and density variations) make no contribution to the COM–COF offset, the full projected 1.31 km offset would be produced entirely by mantle density variations.

For the 0 km bound, we assume the opposite (i.e., the COM-COF offset can be fully explained by either crustal density or topography variations, see our earlier response to the reviewer's fourth comment). As the reviewer mentions, if the COF is due to the shape of the outer surface, this would require either Airy compensation of surface topography (i.e., crustal thickness variations) or Pratt compensation (i.e., crustal density variations) to cancel the effect of the mass displacement (and changes to COM) associated with these patterns. Since Airy or Pratt isostasy should, to first order (e.g., ignoring elastic effects in the crust or slight variations in gravitational forces due to the thickness of the crust) effectively fully compensate the total mass deficit due to topographic variations, we expect that the assigned crustal density should be independent of the COM-COF offset in this scenario. Put another way, we intend for the '0 km' bound to approximately represent an endmember scenario in which the COM-COF offset can be fully explained by the structure of the crust, and so mantle density variations must not produce additional perturbations to this quantity.

We recognize, of course, that additional scenarios could theoretically exist for Mars which would not be represented by our 0 - 1.31 km bounds (e.g., if the crustal COM-COF offset would produce a '2 km' displacement in the direction of the 45N, 138W, then mantle density variations would need to impact the COM-COF offset be -0.69 km to explain observations). While it is possible to design our bounds to accommodate a wider range of such scenarios, we note that the inclusion of the COM–COF offset constraint (at least, in the revised version of the manuscript) is mainly to demonstrate that we would require an implausibly large COM-COF offset (approximately 50× the magnitude of observations) to explain observed asymmetries in terms of compositional variations, which suggests that inferred anomalies in the Southern highlands mantle are instead largely thermal.

Based on this discussion, we have made the following changes:

→ We have incorporated additional discussion regarding our inference of the 1.31 km bound (i.e., projection of COM-COF offset values from Table 4 of Smith et al. (2001)) into the newly added "Modeling Temperature and Iron Content" section of the Methods (i.e., lines 823-844). In this added text, we directly state the COM-COF offsets for Mars and mention the likely uncertainties for these parameters based on the above discussion. In addition, we note that this range is not representative of all possible scenarios for density variations in the mantle (as mentioned above).

→ In the caption of Figure 3, we incorporate additional discussion regarding assumptions that are used to justify the '0' km bound in Figure 3 (e.g., Airy/Pratt isostasy of this topography)

In Eq. 13 rMoho is constant. However, with the assumed crust thickness variation rMoho varies between southern and northern hemisphere. Which value has been chosen and how large is the introduced error? If only the topography is considered for the COM-COF offset without crust thickness variation, what are the results when crust thickness variations are considered? The contribution from the mantle is likely be smaller – possibly even not needed. The red shaded area will be narrower and/or shifted?

Response: Thank you for pointing this out. From Equation 14 in the main text, the COM–COF offset associated with a degree-1 density anomaly distributed through the mantle is

$$\delta z_{COM-COF}^{mantle} = \frac{1}{M}\sqrt{\frac{\pi}{12}}\,\Delta\rho_{mantle}\left(\bar{r}_{Moho}^4 - r_{CMB}^4\right), \tag{R1}$$

where $\bar{r}_{Moho}$ is the mean Moho radius and $\Delta\rho_{mantle}$ is the peak-to-peak hemispheric density contrast within the mantle. To evaluate the effect of a degree-1 variation in Moho depth, we instead write the Northern and Southern Moho radii as

$$r_{Moho}^N = \bar{r}_{Moho} + \frac{\Delta r_{Moho}}{2}, \qquad r_{Moho}^S = \bar{r}_{Moho} - \frac{\Delta r_{Moho}}{2},$$

where Δr_{Moho} is the peak-to-peak hemispheric variation in Moho radius. The corresponding COM–COF contribution from Moho relief is then controlled by the density contrast across the Moho, $\Delta\rho_M = \rho_{mantle} - \rho_{crust}$, and is

$$\delta z_{COM-COF}^{Moho} = \frac{1}{2M}\sqrt{\frac{\pi}{12}}\,\Delta\rho_M\left[\left(r_{Moho}^N\right)^4 - \left(r_{Moho}^S\right)^4\right]. \tag{R2}$$

Substituting $r_{Moho}^{N,S} = \bar{r}_{Moho} \pm \Delta r_{Moho}/2$ gives

$$\delta z_{COM-COF}^{Moho} = \frac{1}{M}\sqrt{\frac{\pi}{12}}\,\Delta\rho_M\left[\bar{r}_{Moho}^3\Delta r_{Moho} + \frac{1}{4}\bar{r}_{Moho}\Delta r_{Moho}^3\right]. \tag{R3}$$

Relative to the mantle contribution in Equation R1, the ratio is therefore

$$\frac{\delta z_{COM-COF}^{Moho}}{\delta z_{COM-COF}^{mantle}} = \frac{\Delta\rho_M}{\Delta\rho_{mantle}}\frac{\bar{r}_{Moho}^3\Delta r_{Moho} + \frac{1}{4}\bar{r}_{Moho}\Delta r_{Moho}^3}{\bar{r}_{Moho}^4 - r_{CMB}^4}. \tag{R4}$$

For a representative mean Moho radius of $\bar{r}_{Moho} = 3340$ km, a core–mantle boundary radius of $r_{CMB} = 1830$ km, a hemispheric Moho variation of $\Delta r_{Moho} = 25$ km, a crust–mantle density contrast of $\Delta\rho_M \sim 600$ kg m^{-3}, and a representative mantle density anomaly of $\Delta\rho_{mantle} \sim 10$ kg m^{-3}, we obtain

$$\frac{\delta z^{Moho}_{COM-COF}}{\delta z^{mantle}_{COM-COF}} \sim 0.5.$$

A hemispheric Moho variation of this magnitude could contribute to the COM–COF offset at the level of roughly 50%, which is certainly not negligible. However, we should point out that variations in surface topography (i.e., Airy isostasy) would create a variation in COM change in the opposite direction of that produced by Moho depth variations:

$$\delta z^{topo}_{COM-COF} = \frac{1}{2M}\sqrt{\frac{\pi}{12}}\,\rho_c\left[\left(r^N_{surf}\right)^4 - \left(r^S_{surf}\right)^4\right], \tag{R5}$$

where $r^N_{surf} = \bar{R} - \Delta h/2$ and $r^S_{surf} = \bar{R} + \Delta h/2$ for an elevated Southern Highlands surface, $\bar{R}$ is Mars's mean radius, Δh is the peak-to-peak hemispheric topographic variation, and ρ_c is crustal density. Expanding to leading order gives

$$\delta z^{topo}_{COM-COF} \approx -\frac{1}{M}\sqrt{\frac{\pi}{12}}\,\rho_c\bar{R}^3\Delta h. \tag{R6}$$

Under Airy isostasy,

$$\rho_c\Delta h \approx (\rho_m - \rho_c)\Delta r_{Moho},$$

and so

$$\delta z^{topo}_{COM-COF} \approx -\frac{1}{M}\sqrt{\frac{\pi}{12}}\,(\rho_m - \rho_c)\bar{R}^3\Delta r_{Moho}. \tag{R7}$$

This has nearly the same magnitude as the Moho contribution, differing only by the replacement $\bar{r}^3_{Moho} \to \bar{R}^3$. For $\bar{R} = 3390$ km and $\bar{r}_{Moho} = 3340$ km, the ratio is $(\bar{R}/\bar{r}_{Moho})^3 \approx 1.04$, so the two effects nearly cancel in the total COM shift. The net effect of these two contributions is therefore approximately zero change in COM position, though not necessarily in COF position. To estimate the latter, we ignore any contribution to the COM and consider only the geometric effect of a degree-1 surface topography anomaly. For a surface of the form

$$r_{surf}(\theta) = \bar{R} + a\cos\theta,$$

where a is the amplitude of the degree-1 topography, the center of figure is given by the centroid of the enclosed volume:

$$\delta z^{topo}_{COF} = \frac{\int_V z\,dV}{\int_V dV}.$$

Expanding to first order in $a/\bar{R} \ll 1$ gives

$$\delta z_{COF}^{topo} \approx a = \frac{\Delta h}{2}, \tag{R8}$$

where Δh is the peak-to-peak topographic contrast associated with the degree-1 shape. Thus, a hemispheric topographic contrast of a few kilometers naturally produces a COF offset of order kilometer (which would be captured by our '0 km' bound used in Figure 3). In any case, we would like to emphasize that changes in COM–COF offset produced by Moho depth variations remain much smaller than the offsets expected if asymmetries in Mars's interior were attributable solely to compositional variations in mantle structure (see discussion above).

Based on the above discussion, we have made the following changes to the manuscript

→ We have altered the text of the section 'Modeling Temperature and Iron Content' (lines 834-844) to note that Moho depth changes can alter COM-COF offsets by up to roughly 50%, but these variations would be negated by mirrored topography at the surface (i.e., Airy compensation). In this added text, we also emphasize that these potential uncertainties are much smaller than the change in COM–COF (several tens of km) needed to explain mantle asymmetries from our tidal tomography result in terms of compositional variations.

Equation 14 to calculate the density variation: Mars mantle composition has a higher iron content; this might change the assumed parameters. Furthermore, how realistic is it to apply this equation for the entire mantle, in particular considering that there is likely a density crossover between mantle material and melt at a depth of about 600-700 km.

Response: Thank you for the comment. We agree that two clarifications are needed here. First, Equation 14 (now Equation 18) is written in terms of density *changes* relative to a reference mantle, not the absolute iron content of the Martian mantle. As such, the fact that Mars has a high bulk iron content does not directly affect our calculation; it only matters insofar as it changes the assumed sensitivity $\frac{d\rho}{dX_{\mathrm{Fe}}}$. Because Equation 18 is linear in $\Delta\rho$, any fractional change in the adopted $\frac{d\rho}{dX_{\mathrm{Fe}}}$ produces the same fractional change in the inferred COM–COF contribution.

To make this more concrete, for a 1% iron enrichment and our adopted density sensitivity of

$$\frac{d\rho}{dX_{\mathrm{Fe}}} = 9.7\,\mathrm{kg\,m^{-3}\,\%^{-1}},$$

Equation 14 predicts a COM–COF contribution of approximately

$$\delta z_{COM-COF} \approx 0.88\ \mathrm{km}.$$

A 5% change in the assumed density sensitivity would therefore change this value by only

$$0.05 \times 0.88\ \mathrm{km} \approx 0.04\ \mathrm{km}.$$

Thus, modest uncertainty in the density sensitivity has only a minor effect on the COM–COF offset predicted for a given compositional anomaly.

Second, we agree that Equation 18 is only a depth-averaged, first-order estimate and does not capture all depth-dependent effects, including a possible density crossover near 600–700 km depth.

To assess the magnitude of this effect, we can split Equation 14 into two radial contributions, one from the upper 700 km of the mantle and one from the deeper mantle:

$$\delta z_{COM-COF} \propto \Delta\rho_{upper} \left(r_{Moho}^4 - r_{700}^4\right) + \Delta\rho_{lower} \left(r_{700}^4 - r_{CMB}^4\right),$$

where $r_{700} = r_{Moho} - 700$ km. For $r_{Moho} = 3340$ km and $r_{CMB} = 1830$ km, the upper 700 km contributes approximately 67% of the total radial weighting in Equation 18, while the lower mantle contributes the remaining 33%. Therefore, if the density sensitivity in only the upper 700 km were changed by 5%, the total COM–COF offset predicted for a 1% iron enrichment would change by only

$$0.67 \times 0.05 \times 0.88 \text{ km} \approx 0.03 \text{ km}.$$

Similarly, a 5% change confined to the lower mantle would alter the predicted offset by only

$$0.33 \times 0.05 \times 0.88 \text{ km} \approx 0.01 \text{ km}.$$

These values are negligible compared to the tens of kilometers in COM–COF offset that would be required to explain the inferred shear modulus anomaly using composition alone. Our conclusion that the inferred asymmetry is dominantly thermal is therefore insensitive to modest uncertainty in both the absolute density sensitivity and its depth dependence.

Based on this discussion, we have made the following changes:

→ We have added text in the section "Modeling Temperature and Iron Content" (lines 819-822) to clarify that Equation 18 is formulated in terms of lateral density perturbations within the mantle, so the absolute mantle density and baseline iron content do not directly affect calculations, except insofar as they modify the assumed sensitivity of density to iron enrichment (that is, the transfer function in Equation 12 of the manuscript).

→ We have added text in 'Modeling Temperature and Iron Content' (lines 839-841) to clarify that we expect uncertainty in the COM-COF offset due to a potential density crossover in the deep mantle to be less than 0.1 km, which is much smaller than the COM-COF offset required to explain asymmetries purely in terms of compositional changes.

> *8. Fig. 3 caption: "we infer a partial melt anomaly of >2% by volume as well as up to 60% pyroxene depletion over the Southern highlands mantle." Why 60% pyroxene depletion? On the figure it looks like up to 75% pyroxene.*

Response: Thank you for the comment. In the original version of the manuscript, we based the range of potential pyroxene depletion (i.e., up to 60%) on the overlap of the preferred shear modulus variation and the COM-COF offset constraint (i.e., the black line in Figure 3) rather than the overlap of the 99.7% shear modulus constraint with the COM-COF offset constraint. Considering the latter, we would have expected up to 75% pyroxene depletion (as the reviewer notes).

This distinction is now somewhat moot since, in response to the reviewer's first comment, we have altered our inference of partial melt to an inference of temperature variation and Fa-Fo mol fraction variations. Nonetheless, we agree that in the updated version of the manuscript, we should use the full overlap between the 99.7% shear modulus constraint and COM-COF offset range when stating values for inferred compositional changes based on the analysis in Figure 3. We have therefore made the following changes:

→ We have altered text throughout the manuscript to ensure we state an iron enrichment of up to 5% based on the overlap between the 99.7% shear modulus constraint and COM-COF offset range in Figure 3.

→ We have altered the caption of the updated Figure 3 to clarify that the inferred up to 5% iron enrichment is based on the overlap between the 99.7% shear modulus constraint and COM-COF offset range.

> *9. Line 561: The authors simply assume a reference value for the shear moduls of the mantle, crust and core. Can a variation of these reference values influence the results?*

Response: Thank you for the comment. Generally speaking, no. This is because changes in the degree-0 (i.e., 1D) shear modulus values assigned to the mantle, crust, and core are (in isolation) expected to only substantially impact the within-harmonic response of the interior to tidal forces (i.e., the non-mode coupled component of response). This would correspond to, for example, forcing at degree-3 order-1 producing a response at degree-3 order-1. The expected within-harmonic component of forcing at Mars is much (at least 3 orders of magnitude) smaller than mode coupled components of response reported in Table 1 of the manuscript. This follows from Equation 4 of the manuscript, which predicts that forcing above $\ell = 2$ rapidly diminishes with increasing spherical harmonic according to $\sim \frac{R^{\ell-2}}{r^{\ell-2}}$ (r is the distance of the Sun and R is Mars's radius). Based on this relation, we expect Mars's degree-3 forcing to be approximately 1×10^{-5} smaller than degree-2 forcing (note, this is much smaller than the ratio of degree-3 to degree-2 forcing at the Moon of ∼200, which is the reason Park et al. (2025) consider 1D shear modulus variation for tidal tomography inversions in their work).

Even so, changes in the degree-0 shear modulus can conditionally have an impact on Mars's mode coupled response *if* there is 3D structure. Even in this case, the effect is only to impact the absolute 3D shear modulus variation (i.e., in terms of dimensional units e.g. Giga-Pascals) that is needed to explain a given signal. Since our inversions directly normalize 3D shear modulus variations by reference values into percentages (see the LHS of Equation 7), these potential impacts are implicitly accounted for in our method.

As an additional check, we have carried out tidal tomography inversions for which we sample the 1D shear and bulk moduli of the crust and mantle from a Gaussian distribution centered about our nominal reference model values (see Table 1 of the manuscript text) and with a standard deviation equal to 5% of these values (see "Reference ±5%" in R.9 above). The resulting posterior distributions for recovered degree-1 variations in mantle shear modulus are essentially indistinguishable from those of our nominal inversion.

1D variations could also theoretically impact the inferred order-dependent degree-2 terms used as constraints on the inversion (see response to earlier comment), though we find pretty good agreement between our recovered value (from gravity field inversions) and model predictions (see Extended Data Figure 1a), and do not attempt to further constrain 1D variations based on this parameter (which is beyond the scope of the current work).

In any case, we recognize the need to make more clear in the manuscript the impact (or lack thereof) of the choice of reference model on our inversion results. We have therefore made the following changes:

→ We have added text in lines 745-749 to clarify that, although the adopted 1D reference shear modulus could affect the non-mode-coupled response of Mars's interior, direct forcing at degree 3 is expected to be negligible relative to the mode-coupled degree-3 signals used in our inversion and is therefore ignored.

→ We have added text immediately after Equation 12 (lines 754-757) to state explicitly that, because the inverted 3D shear modulus perturbations are normalized by 1D reference shear moduli, the influence of the adopted reference model on the recovered amplitudes is implicitly accounted for in our method.

→ We have incorporated the "Reference ±5%" case discussed above into Extended Data Fig. 9 of the revised manuscript.

10. Line 569-570 "We do not consider lateral changes in density (i.e., which may violate constraints from static gravity measurements.." The authors predict a variation in depletion which is necessarily attributed to density variation in the mantle. It looks like that they violate their own assumption.

Response: Thank you. In principle, it is possible to jointly use the static gravity (including COM-COF offset) and time-variable gravity to constrain Mars's mantle structure, but this procedure would involve simultaneously solving for variations in the thickness and density of the crust. This crustal thickness estimation is complicated, and involves several additional modelling assumptions (e.g., a porosity model for the crust, see e.g., Wieczorek et al. (2013)). We correspondingly believe it would be beyond the scope of the current work to jointly perform this inversion alongside tidal tomography, but is a potential area of useful future research.

We therefore do not incorporate the COM-COF observation directly into tidal tomography inversions but only consider this constraint in an a-posteriori sense for comparison to inferred shear modulus asymmetries. However, as the reviewer notes, we agree that it is important to more clearly explain the trade-off between the structure of the mantle/crust for our interpretation of the COM-COF and shear modulus variations for these a-posteriori calculations. Therefore, aside from the changes to the manuscript discussing the full range of effects impacting the COM-COF offset for Mars noted above, we have made the following additional changes:

→ In line with our response to an earlier comment by the reviewer, we have modified the caption of Figure 3 to note that interpretations of COM-COF offset are sensitive to both the structure of the crust and mantle.

→ We have modified lines 741-742 to clarify that COM-COF offset is used only for a-posteriori calculations described in Figure 3.

11. Line 217-222 "For example, if upwelling below the Southern highlands occurred after the initial crystallization of Mars's primordial crust, the resulting thermal anomaly could have raised parts of the solid lower crust above the Curie temperature [14]. This heating, in turn, would have allowed affected crust to acquire magnetization from Mars's ancient dynamo between 4.5 and 4.1 Gya [13, 46]." Not sure that I understand this argumentation. The higher heat flow in the southern hemisphere reduces the thickness of the remanent magnetized layer here, but we see a stronger field in the southern hemisphere. What do the authors want to explain here?

Response: Thank you for the comment. We should clarify that whether or not the Southern highlands' crust's remnant magnetized layer becomes thinner or thicker depends on the timing of the cessation of the dynamo relative to when maximal heating associated with the crustal dichotomy occurs. For example, if the thermal anomalies result in a temperature that is broadly higher than the Curie temperature following cessation of the dynamo, then this would result in a thinner magnetized layer over the Southern highlands (as the reviewer notes). By contrast, if thermal anomalies over the Southern highlands elevated temperatures above the Curie temperature prior to cessation of the dynamo, but temperatures also dropped below the Curie temperature prior to dynamo cessation, then we would expect this region to be more magnetized than the northern hemisphere crust. The latter scenario is more consistent with the observed stronger magnetization of the Southern Highlands, and is the case we intend to highlight in the manuscript.

As pointed out by the reviewer, these nuances (and, in particular, the relationship between the cessation of the dynamo and heating) are not clearly discussed in the current version of the manuscript. We have therefore made the following changes:

→ We have revised discussion of Southern highlands' crustal magnetization in lines 234-240 to clarify that the effect of a thermal anomaly on the magnetization of the Southern highlands crust depends on its timing relative to the cessation of Mars's ancient dynamo. In particular, the revised text now states heating above the Curie temperature followed by cooling before dynamo cessation could allow the Southern Highlands crust to acquire the stronger remanent magnetization observed today.

> *Minor comments: 12. Line 68: The definition of resolution of the gravity field. I believe that here you are assuming that you need to resolve an entire wavelength, rather than half, as used by other authors. Worth mentioning.*

Response: Thank you for the comment. We agree that the text should be more precise about the convention being used. Here, we use a full-wavelength estimate for the characteristic spatial scale associated with spherical harmonic degree ℓ, namely $\sim 2\pi R/\ell$. To make this clear, we have made the following change to the manuscript:

→ We have modified lines 67–69 of the manuscript to clarify that $\sim 2\pi R/\ell$ refers to a full-wavelength at a given degree.

> *13. Line 77: This depends on the wavelength. Long-wavelength static gravity, exactly like tides, is more sensitive to the deep interior. Rephrase this sentence. As it is written it's incorrect.*

Response: Thank you. We certainly agree that long-wavelength static gravity fields have greater sensitivity to deep interior structure than shorter-wavelength static fields. Our intended point, however, was that even at long wavelengths the static gravity field are *most* sensitive to shallow density structure (i.e., compared to deeper structure). This can be inferred from the dependence of static gravity on internal density structure over a given ℓ (see e.g., Eq. 4 of Hemingway and Mittal (2019)) which is weighted by $r^{\ell+2}$, where r is radial distance from the COM of the body (and has a maximum value at R, the outer radius). This contrasts with the low-order non-zonal time-variable

gravity field's sensitivity to shear modulus variations considered in our manuscript, which are most sensitive to variations in the deep mantle (see e.g., Fig 1b of our manuscript).

To make this more clear, we have made the following changes to the manuscript:

→ We have modified the sentence in lines 78-84 to clarify that, although long-wavelength static gravity fields are sensitive to deep interior density structure, their signal remains most sensitive to density variations in the uppermost crust. We clarify that this stands in contrast to the low-order non-zonal time-variable gravity field considered here, which is most sensitive to shear modulus variations in the deep mantle.

> *14. Table 1: Although mentioned in the methods, it is absolutely unclear from the main text what is the frequency of the periodic gravity signals that are estimated.*

Response: Thank you for this comment. We agree that the period of the estimated degree-3 non-zonal gravity coefficient variations could be more directly stated in the main text of the manuscript (i.e., outside of the methods or Table 1 caption). We have therefore made the following change:

→ We have modified text in the first paragraph of the 'Measuring Mars's Seasonal Tidal Response' section (lines 93-96) to clarify that we estimate and report coefficients at the seasonal (or annual) timescale, corresponding to a 687-day period.

> *15. Line 603: The supplementary information discusses how the likelihood function is designed. In the discussion a covariance of the model is introduced but not defined. How is this computed?*

Response: Thank you for pointing this out. We agree that the description of the covariance term in the original manuscript was unclear. In the original implementation, the quantity denoted as "model covariance" was not computed from forward-model errors. Rather, it was introduced only as a bookkeeping term so that the total covariance used in the likelihood corresponded to the 15× formal uncertainties discussed earlier in the manuscript. This information was mistakenly left out of the methods section text and, in any case, is unnecessarily indirect (i.e., it would be clearer to describe the likelihood in terms of the observational covariance alone). We have therefore made the following change:

→ We have revised the text immediately following Equation 14 (lines 786-790) to clarify that the covariance matrix used in the likelihood is defined directly from the 15×-inflated formal uncertainties of the gravity coefficients listed in Table 1. We have also removed mention of model covariance in the updated text.

References (Referee #1)

Belleguic, V., P. Lognonné, and M. Wieczorek (2005). "Constraints on the Martian lithosphere from gravity and topography data". In: *Journal of Geophysical Research: Planets* 110.E11, E11005. DOI: 10.1029/2005JE002437.

Berne, Alexander et al. (2026). "[In preparation: Berne et al. 2026]". In: *Manuscript in preparation*. Placeholder entry — update with full reference once available.

Berne, Alexander et al. (2023a). "Inferring the mean thickness of the outer ice shell of Enceladus from diurnal crustal deformation". In: *Journal of Geophysical Research: Planets* 128.6, e2022JE007712. DOI: 10.1029/2022JE007712.

— (2023b). "Using tidally-driven elastic strains to infer regional variations in crustal thickness at Enceladus". In: *Geophysical Research Letters* 50.—, pp. 311–318. DOI: 10.1029/2023GL106656.

Berne, Alexander et al. (2024). "Jet activity on Enceladus linked to tidally driven strike-slip motion along tiger stripes". In: *Nature Geoscience* 17, pp. 385–391. DOI: 10.1038/s41561-024-01418-0.

Bruinsma, Sean and Frank G. Lemoine (2002). "A preliminary semiempirical thermosphere model of Mars: DTM-Mars". In: *Journal of Geophysical Research: Planets* 107.E10, p. 5085. DOI: 10.1029/2001JE001508.

Cheng, Kar Wai et al. (2024). "Mars's Crustal and Volcanic Structure Explained by Southern Giant Impact and Resulting Mantle Depletion". In: *Geophysical Research Letters*. DOI: 10.1029/2023GL105910.

Citron, Robert I., Michael Manga, and Eh Tan (2018). "A hybrid origin of the Martian crustal dichotomy: Degree-1 convection antipodal to a giant impact". In: *Earth and Planetary Science Letters* 491, pp. 58–66. DOI: 10.1016/j.epsl.2018.03.031.

Dalton, Colleen A. and Ulrich H. Faul (2010). "The oceanic and cratonic upper mantle: Clues from joint interpretation of global velocity and attenuation models". In: *Lithos* 120.1–2, pp. 160–172. DOI: 10.1016/j.lithos.2010.08.020.

Faul, Ulrich H. and Ian Jackson (2015). "Transient Creep and Strain Energy Dissipation: An Experimental Perspective". In: *Annual Review of Earth and Planetary Sciences* 43, pp. 541–569. DOI: 10.1146/annurev-earth-060313-054732.

Genova, A. et al. (July 2016). "Seasonal and static gravity field of Mars from MGS, Mars Odyssey and MRO radio science". In: *Icarus* 272, pp. 228–245. DOI: 10.1016/j.icarus.2016.02.050.

Genova, Antonio et al. (2015). "Long-term variability of CO2 and O in the Mars upper atmosphere from MRO radio science data". In: *Journal of Geophysical Research: Planets* 120.5, pp. 849–868. DOI: 10.1002/2014JE004770.

Gibet, Valentin Bonnet et al. (2022). "A Positive Feedback Between Crustal Thickness and Melt Extraction for the Origin of the Martian Dichotomy". In: *Journal of Geophysical Research: Planets* 127.12, e2022JE007472. DOI: 10.1029/2022JE007472.

Goossens, Sander et al. (2017). "Evidence for a low bulk crustal density for Mars from gravity and topography". In: *Geophysical Research Letters* 44.15, pp. 7686–7694. DOI: 10.1002/2017GL074172.

Guo, X. et al. (2009). "Fitting the Viking lander surface pressure cycle with a Mars general circulation model". In: *Journal of Geophysical Research: Planets* 114.E7, E07006. DOI: 10.1029/2008JE003302.

Hemingway, Douglas J. and Tushar Mittal (2019). "Enceladus's Ice Shell Structure as a Window on Internal Heat Production". In: *Icarus* 332, pp. 111–131. DOI: 10.1016/j.icarus.2019.03.011. URL: https://doi.org/10.1016/j.icarus.2019.03.011.

Jackson, Ian and Ulrich H. Faul (2010). "Grainsize-sensitive viscoelastic relaxation in olivine: Towards a robust laboratory-based model for seismological application". In: *Physics of the Earth and Planetary Interiors* 183.1–2, pp. 151–163. DOI: 10.1016/j.pepi.2010.09.005.

Justh, H. L. (2014). *Mars Global Reference Atmospheric Model 2010 Version: Users Guide*. Tech. rep. NASA/TM-2014-217499. Huntsville, AL: NASA Marshall Space Flight Center.

Kim, D. et al. (2023). "Global Crustal Thickness Revealed by Surface Waves Orbiting Mars". In: *Geophysical Research Letters* 50.12, e2023GL103482. DOI: 10.1029/2023GL103482.

Konopliv, A. S., R. S. Park, and W. M. Folkner (2016). "An Improved Mars Gravity Field and Love Number from MRO, Mars Odyssey, and MEX Tracking Data". In: *Icarus* 274, pp. 253–260. DOI: 10.1016/j.icarus.2016.02.052.

Lau, Harriet C. P. and Ulrich H. Faul (2019). "Anelasticity from seismic to tidal timescales: Theory and observations". In: *Earth and Planetary Science Letters* 508, pp. 18–29. DOI: 10.1016/j.epsl.2018.12.009.

Madeleine, J.-B. et al. (2011). "Revisiting the radiative impact of dust on Mars using the LMD Global Climate Model". In: *Journal of Geophysical Research: Planets* 116, E11010. DOI: 10.1029/2011JE003855.

Marinova, Margarita M., Oded Aharonson, and Erik Asphaug (2008). "Mega-impact formation of the Mars hemispheric dichotomy". In: *Nature* 453.7199, pp. 1216–1219. DOI: 10.1038/nature07070. URL: https://doi.org/10.1038/nature07070.

McCarthy, Christine, Yasuko Takei, and Tomohiko Hiraga (2011). "Experimental study of attenuation and dispersion over a broad frequency range: 2. The universal scaling of polycrystalline materials". In: *Journal of Geophysical Research: Solid Earth* 116.B9, B09207. DOI: 10.1029/2011JB008384.

Millour, E et al. (n.d.). *THE MARS CLIMATE DATABASE (VERSION 5.3)*. Tech. rep. URL: http://www-mars.lmd.jussieu.fr.

Montabone, L. et al. (2006). "Validation of martian meteorological data assimilation for MGS/TES using radio occultation measurements". In: *Icarus* 185.1, pp. 113–132. DOI: 10.1016/j.icarus.2006.07.012.

Nimmo, F. and U. H. Faul (2013). "Dissipation at tidal and seismic frequencies in a melt-free, anhydrous Mars". In: *Journal of Geophysical Research: Planets* 118.12, pp. 2558–2569. DOI: 10.1002/2013JE004499.

Nimmo, F., U. H. Faul, and E. J. Garnero (2012). "Dissipation at tidal and seismic frequencies in a melt-free Moon". In: *Journal of Geophysical Research: Planets* 117.E9, E09005. DOI: 10.1029/2012JE004160.

Ojha, Lujendra et al. (2020). "Groundwater production from geothermal heating on early Mars and implication for early martian habitability". In: *Science Advances* 6.49, eabb1669. DOI: 10.1126/sciadv.abb1669.

Park, R. S. et al. (2025). "Thermal asymmetry in the Moon's mantle inferred from monthly tidal response". In: *Nature* 641, pp. 1188–1192. DOI: 10.1038/s41586-025-08949-5.

Plesa, Ana C. et al. (2018). "The thermal state and interior structure of Mars". In: *Geophysical Research Letters* 45.22, pp. 12198–12209. DOI: 10.1029/2018GL080728. URL: https://doi.org/10.1029/2018GL080728.

Plesa, Ana-Catalina et al. (2016). "How Large Are Present-Day Heat Flux Variations Across the Surface of Mars?" In: *Journal of Geophysical Research: Planets* 121.12. Verify: cited as "Plesa et al. 2017" in the response text., pp. 2386–2403. DOI: 10.1002/2016JE005126.

Plesa, Ana-Catalina et al. (2022). "Interior dynamics and thermal evolution of Mars — a geodynamic perspective". In: *Advances in Geophysics*. Vol. 63. Elsevier, pp. 179–230. DOI: 10.1016/bs.agph.2022.07.005.

Reese, C. C., C. P. Orth, and V. S. Solomatov (2011). "Impact megadomes and the origin of the martian crustal dichotomy". In: *Icarus* 213.2, pp. 433–442. DOI: 10.1016/j.icarus.2011.03.028.

Roberts, James H. and Shijie Zhong (2006). “Degree-1 convection in the Martian mantle and the origin of the hemispheric dichotomy”. In: *Journal of Geophysical Research* 111.E06013. DOI: 10.1029/2005JE002668.

Samuel, Henri et al. (2023). “Geophysical evidence for an enriched molten silicate layer above Mars’s core”. In: *Nature* 622, pp. 712–717. DOI: 10.1038/s41586-023-06601-8. URL: https://www.nature.com/articles/s41586-023-06601-8.

Shaw, Denis M. (1970). “Trace element fractionation during anatexis”. In: *Geochimica et Cosmochimica Acta* 34.2, pp. 237–243. DOI: 10.1016/0016-7037(70)90009-8.

Smith, David E. et al. (1997). “Topography of the Moon from the Clementine Lidar”. In: *Journal of Geophysical Research: Planets* 102.E1, pp. 1591–1611. DOI: 10.1029/96JE02940. URL: https://doi.org/10.1029/96JE02940.

Smith, David E. et al. (2001). “Mars Orbiter Laser Altimeter: Experiment summary after the first year of global mapping of Mars”. In: *Journal of Geophysical Research: Planets* 106.E10, pp. 23689–23722. DOI: 10.1029/2000JE001364.

Stähler, Simon C. et al. (2021). “Seismic detection of the Martian core”. In: *Science* 373.6553, pp. 443–448. DOI: 10.1126/science.abi7730. URL: https://www.science.org/doi/10.1126/science.abi7730.

Sun, Xia and Hrvoje Tkalčić (2024). “Constraints on the Origin of the Martian Dichotomy From Southern Highlands Marsquakes”. In: *Geophysical Research Letters* 51.8, e2024GL110921. DOI: 10.1029/2024GL110921. URL: https://doi.org/10.1029/2024GL110921.

Thiriet, Mélanie et al. (2018). “Hemispheric Dichotomy in Lithospheric Thickness on Mars Caused by Differences in Crustal Structure and Composition”. In: *Journal of Geophysical Research: Planets* 123.4, pp. 823–848. DOI: 10.1002/2017JE005431.

Wagner, N. L. et al. (June 2024). “Evaluating the Use of Seasonal Surface Displacements and Time-Variable Gravity to Constrain the Interior of Mars”. In: *Journal of Geophysical Research: Planets* 129 (6). ISSN: 2169-9097. DOI: 10.1029/2023JE008053.

Wieczorek, Mark A. and Maria T. Zuber (2004). “Thickness of the Martian crust: Improved constraints from geoid-to-topography ratios”. In: *Journal of Geophysical Research: Planets* 109.E1, E01009. DOI: 10.1029/2003JE002153.

Wieczorek, Mark A. et al. (2013). “The Crust of the Moon as Seen by GRAIL”. In: *Science* 339.6120, pp. 671–675. DOI: 10.1126/science.1231530.

Wieczorek, Mark A. et al. (2022). “InSight Constraints on the Global Character of the Martian Crust”. In: *Journal of Geophysical Research: Planets* 127.5, e2022JE007298. DOI: 10.1029/2022JE007298. URL: https://doi.org/10.1029/2022JE007298.

Yamauchi, Hatsuki and Yasuko Takei (2016). “Polycrystal anelasticity at near-solidus temperatures”. In: *Journal of Geophysical Research: Solid Earth* 121.11, pp. 7790–7820. DOI: 10.1002/2016JB013316.

Zhong, Shijie and Maria T. Zuber (2001). “Degree-1 mantle convection and the crustal dichotomy on Mars”. In: *Earth and Planetary Science Letters* 189.1–2, pp. 75–84. DOI: 10.1016/S0012-821X(01)00345-4.

Referee #2

In this study, A. Berne and collaborators investigated potential lateral heterogeneity in Mars's internal structure by analyzing its gravitational response to seasonal tidal interactions with the Sun. For that purpose, they analyzed radio tracking data from multiple Martian orbiters (Mars Global Surveyor, Mars Odyssey, Mars Reconnaissance Orbiter), over a total of 16 years. The present study extends previous analyses evaluating the temporal evolution of the zonal terms at degrees 2 and 3 (Genova et al. 2016, Konopliv et al. 2016) by assessing the non-zonal terms.

Response: Thank you for this summary of our work. As discussed below, we have made substantial revisions to the manuscript in response to the comments noted by the reviewer. We hope that the revised manuscript addresses these concerns.

All line numbers referenced in our responses below correspond to the *revised* manuscript. Several sections have been substantially rewritten (e.g., *A Thermal Anomaly Beneath Mars's Southern Highlands*), so many line numbers indicated below will not align with those of the original submission.

Before proceeding, we also note that several of the reviewer's subsequent comments raise closely related concerns regarding (a) the treatment of the zonal ΔC_{20} and ΔC_{30} terms, and (b) the sensitivity of the time-variable gravity signal (and tidal tomography inverison results) to assumptions regarding the atmosphere and adopted loading Love numbers. To keep our response as clear as possible, we have re-arranged some of these sets of comments to provide a single consolidated response to each group. For the reviewer's convenience, we have numbered the comments and responses sequentially below (Comment 1, Comment 2, . . .), with each quoted reviewer comment preceding our corresponding response.

Comment 1.

[. . .] they [. . .] excluded non-zonal degree 2 terms from their analysis.

Response: Thank you for this comment. In our gravity inversion procedure, we solve for order-dependent (i.e., zonal and non-zonal) degree-2 terms (i.e., alongside the zonal and non-zonal degree-3 terms). The original manuscript, however, did not use order-dependent degree-2 terms as a constraint on the likelihood function for tidal tomography inversions (i.e., Equation 14). Our original motivation for this choice was that the degree-2 response is dominated by the main (i.e., within-harmonic) response to the degree-2 tide, and is minimally sensitive to the lateral shear-modulus variations that are the focus of this work.

Regardless, we agree with the reviewer that including order-dependent degree-2 terms would makes our tidal tomography inversion more complete, and we have re-run our analysis with this parameter as an additional constraint on the likelihood function. We find that (i) the recovered 3D shear-modulus structure is essentially unchanged and (ii) there is effectively negligible spread in the posterior distribution for order-dependent degree-2 terms produced by these updated inversions (see Extended Data Figure 1a in the revised manuscript), suggesting minimal impact on our main inversion results (see e.g., Fig. 2 and 3 of the manuscript).

Based on this comment, we have made the following changes (note that these overlap substantially with changes made in response to a comment by reviewer 1):

- → We have revised Figure 2 of the manuscript (as well as Extended data Figures 2 and 3) based on results from inversions which incorporate the degree-2 time variable gravity field terms as an additional constraints. We have also modified Extended data Figure 1 to incorporate posterior distributions (and observations) for the degree-2 time-varying gravity field.
- → We have adjusted the third paragraph of our results section (i.e., 'Modeling Mars's Mantle Structure', lines 145-149) to incorporate discussion of the impact of incorporating degree-2 time variable gravity field as a constraint on inversions. We note in this added text that the lack of deviation between degree-2 terms and expectations for a spherically symmetric interior suggest minimal $\ell = 2$ structure in Mars's mantle.
- → In making changes associated with this comment, we encountered an error in the plot generation script in the original version of the manuscript which impacted the degree-2 order-0,2 histograms in Extended Data Figure 2, which has now been corrected.

Comment 2.

> *However, without sufficient justification, they did not assess the contribution of the seasonal tidal response to the zonal term [. . .].*
>
> *Finally, it is not entirely clear how the gravity signals of atmospheric forcing and the CO_2 cycle load are modeled and taken into account in the analysis. These various elements cast doubt on their analysis and conclusion regarding the signatures of internal heterogeneity in the seasonal tidal response. Further justification and testing are needed to support their analysis and conclusion.*
>
> *As shown by previous studies as well as on Fig. 1 in the Supplementary Information, the zonal term 30 in the time-varying gravity field is much larger than the non-zonal term, by at least one order of magnitude. The time variations of the zonal term 30 over the 16 years covered by MGS, ODY, MRO can be explained at first order by the polar ice cap mass exchanges. The observed 30 gravity signal include also the signatures due to the mantle response to the surface load as well as the 30 component of the seasonal tidal response. It seems to me that their proposed solution would be more robust if they could also reproduce the 30 zonal term and the different contributions (atmospheric/polar ice cap load, mantle response to the surface load, and tidal response).*

Response: Thank you for this comment. The reviewer raises concerns regarding inclusion of the zonal ΔC_{20} and ΔC_{30} terms, and has (correctly) identified three distinct contributions to this signal: (i) the direct gravitational signature of the atmosphere and mass exchange over polar caps (ii) the deformational response of these interior to those surface loads, and (iii) the seasonal solid body tide. Below is a discussion of each of these contributions (and ways that our inversion could account for these variables).

- → *Atmosphere (contribution to the gravity field (i, atmospheric) and surface deformation (ii)).* Following Genova et al. (2016), we compute the atmospheric contribution to the time-variable gravity

field coefficients from surface-pressure grids (Mars-GRAM 2010) as

$$\Delta C_{\ell m}^{\text{atm}}(t) \;=\; \frac{3\,(1+k'_\ell)}{\bar{\rho} g\,R\,(2\ell+1)} \int P_s(\theta,\phi,t)\,Y_{\ell m}^{*}(\theta,\phi)\,d\Omega, \tag{1}$$

where P_s is the time-varying surface pressure, g is the surface gravity, $\bar{\rho}$ and R are Mars's mean density and radius, and k'_ℓ is the onder-independent degree-ℓ loading Love number. The $(1+k'_\ell)$ factor folds the mantle elastic response to the atmospheric load directly into this expression, so that the direct atmospheric contribution and its associated mantle response are treated consistently and do not need to be modelled separately. The justification for adopting onder-independent k'_ℓ (i.e., loading Love numbers that are non-order dependent) in the presence of 3D interior structure is deferred to our response to a separate comment below.

→ *Polar-cap load (contribution to the gravity field (i, cap) and surface deformation (ii)).* In past literature, the seasonal mass exchange over the polar caps has been recovered directly from the observed time-variable zonal gravity field by Genova et al. (2016) (6.2×10^{15} kg for the northern cap and 8.4×10^{15} kg for the southern cap). Because these mass values were obtained directly from the observed zonal coefficients *without* applying a $(1+k'_\ell)$ correction, these mass exchange values implicitly absorb the mantle elastic response. In other words, with our adopted $k'_2=-0.14126$ and $k'_3=-0.09109$ (see response to a later comment), surface deformation would account for ∼8% of the total zonal signal at degree 2 and ∼6% of the degree 3 signal.

→ *Solid-body tidal response (contribution (iii)).* Once contributions (i) and (ii) have been folded into the forward model as above, the only remaining contribution is the seasonal solid-body tidal response.

For context, in the original version of the manuscript (Extended Data Figure 1), our constraints for ΔC_{20} and ΔC_{30} are corrected for the impact of the atmosphere (including both the direct gravitational signal and the deformational response discussed above, see also our response to a later comment on corrections for the atmosphere) but forward models did not incorporate effects related to seasonal polar cap mass exchange, which is why the predicted and observed zonal terms appeared to disagree. Based on this (and the discussion above), we have re-run our inversions incorporating the zonal terms as constraints (and run a parallel set of inversions which fully exclude zonal terms, but incorporates the non-zonal degree-2 gravity field as a constraint, see earlier comment). For these inversions, we assume that zonal terms comprise the sum of the solid body response and the impact of seasonal mass changes of 6.2×10^{15} kg and 8.4×10^{15} kg for the northern and southern caps, respectively (these values are derived from Genova et al. (2016)). Posterior distributions for inversions that include and exclude zonal harmonics are shown below in R.14.

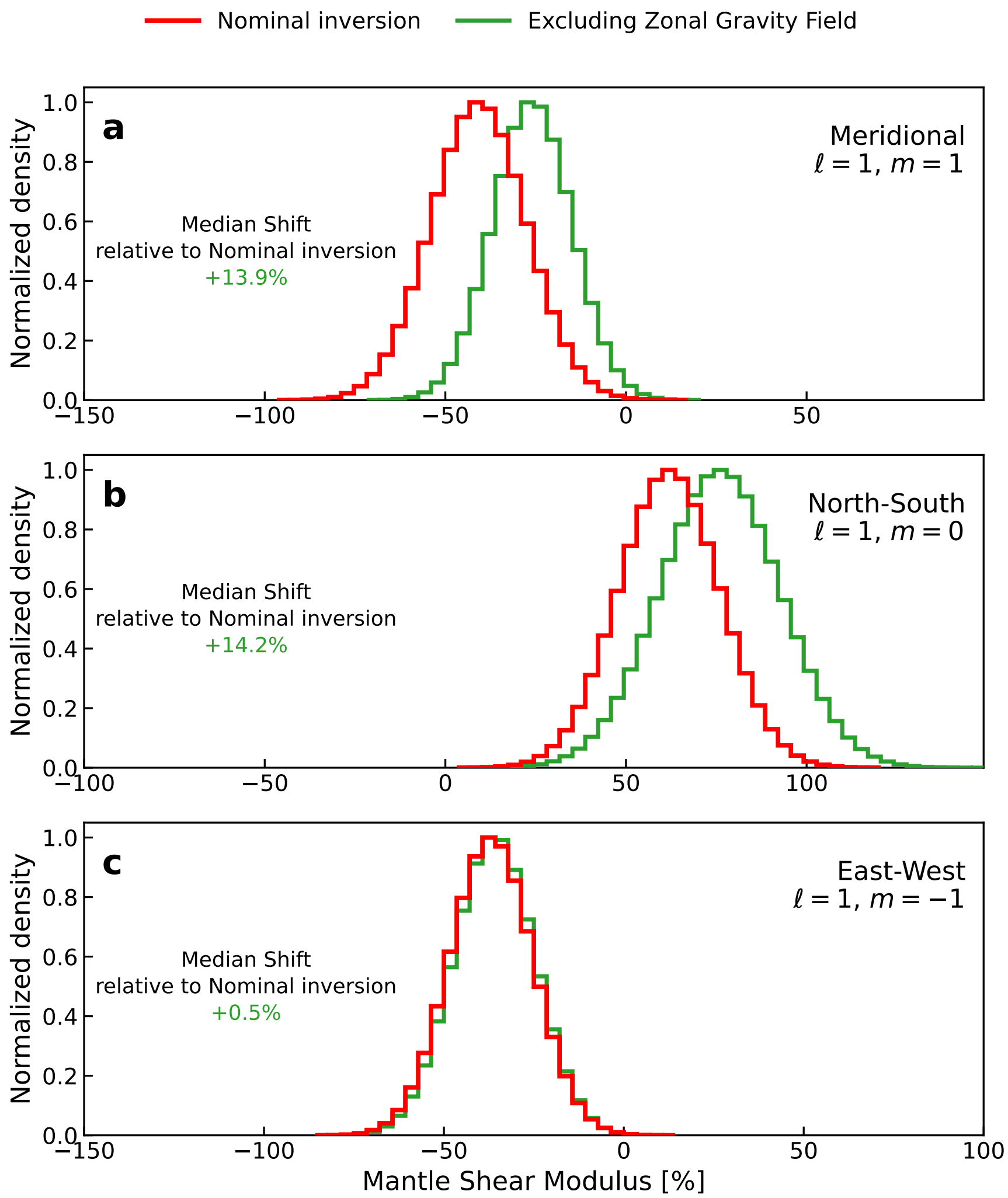

R.14: Comparison of tidal tomography results with an inversion that excludes zonal harmonics. Posterior distributions of mantle shear modulus asymmetries from an inversion that excludes zonal gravity harmonics in Table 1 and Extended Data Table 1 (green) including **(a)** the meridional $\ell = 1, m = 1$ term, **(b)** the north–south $\ell = 1, m = 0$ term, and **(c)** the east–west $\ell = 1, m = -1$ term. Posterior distributions are overlain with results from the nominal tidal tomography inversion presented in Fig. 2 of the revised manuscript (red). Median shifts relative to the nominal inversions are shown to the left in each panel.

As shown in above, the inclusion of zonal terms appears to shift the posterior distributions by up to 15%. We suspect that this shift is mainly attributable to the impact of the ΔC_{30}^{B} term, which shows a relatively wide posterior spread in Extended Data Fig. 1b of the updated manuscript (suggesting that the observational bounds on this parameter are influencing the acceptance of models in MCMCs).

Based on this discussion, we have made the following changes to the manuscript:

→ We have made several updates, including to Figs. 2 and 3, as well as Extended Data Figs

1,2, and 3 and Extended Data Tables 3 and 4, to incorporate the new results that use the time-variable zonal gravity field as constraints on tidal tomography inversions.

→ We have incorporated discussion of the inclusion of zonal harmonics as constraints on inversions (including the impact of this change on posterior distributions) in lines 723-727 of the methodology.

We note that this comment is closely related to comments raised by Referee #1 (specifically, those concerning the treatment of the zonal ΔC_{20} and ΔC_{30} observations), and so our response and the associated changes to the manuscript overlap substantially with our response to those comments.

Comment 3.

> *Furthermore, they focused solely on annual (seasonal) temporal variations, while acknowledging that the degree 3 signal is stronger at diurnal frequencies.*

Response: Thank you for this comment. We focus on the seasonal (rather than diurnal) degree-3 response is because, in our gravity field inversion, we estimate small periodic empirical accelerations in the along- and cross-track directions with characteristic frequencies closely tied to the orbital period ($\sim$ a few hours). These empirical parameters are necessary to absorb residual mismodeling of short-period (e.g., diurnal) non-conservative forces (e.g., drag, radiation pressure), ensuring that the resulting solution reflects only the physically meaningful longer-period variations. But, as a consequence, their inclusion makes it considerably more difficult to cleanly separate a physically meaningful diurnal gravity signal from the other short-period perturbations they are designed to absorb. In other words, correlations between empirical accelerations and a potential diurnal tidal signal introduce substantial additional uncertainty in the recovery of gravity fields at diurnal frequencies. For these reasons, we have focused our analysis on the annual degree-3 variations in this work, though we recognize that attempting to recover diurnal tidal signals could be a useful direction for future work to validate the results in our manuscript.

In any case, we agree with the reviewer that a better discussion/justification for our reasons to focus solely on the seasonal signal (as opposed to the diurnal signal) is warranted in the manuscript. We have therefore made the following changes:

→ We have revised lines 682-688 to clarify that empirical accelerations absorb short-period forces in our gravity field inversion step, making extraction of the diurnal tidal response using our method difficult (hence the reason we do not attempt to recover these signals, and instead focus on seasonal signals).

Comment 4.

> *Based on Figure 1, the predicted impact of the atmosphere appears substantially larger than the effect of the seasonal tidal response. Any error in atmospheric correction can lead to a significant change in the relative contribution of the seasonal tidal response.*
>
> *I did not find any information regarding the assumed value for the load Love number, k2' and k3'. The authors only mention "Mars's loading love numbers k'_ℓ consistent with theoritical predictions (Wagner et al 2024)," without specifying the exact values used. A range of possible values should be considered. The atmospheric component is estimated by the Mars Climate Database, based on the LMD GCM simulations. A discussion regarding the accuracy of the GCM prediction (and possibly comparison with other Mars GCMs) should be provided, as it is a critical aspect of the study. Any error in the atmospheric component can significantly alter the estimated amplitude of the tidal seasonal response.*

Response: Thank you for these comments. The reviewer raises concern regarding (a) the specific loading Love numbers k'_2 and k'_3 adopted in the analysis and (b) the impact of corrections associated with the atmosphere on our inversion results (e.g., the impact of error/variation in the choice of assumed atmospheric model). We address these comments below.

Loading Love numbers.

We use loading Love numbers of $k'_2 = -0.14126$ and $k'_3 = -0.09109$ as inputs into models to estimate the impact of atmospheric loading on Mars's gravity. We compute these values for a spherically symmetric, self-gravitating 1D elastic Mars using the semi-analytic method described in Wagner et al. 2024. The reference radial interior used for these calculations is the same as that used for tidal tomography inversions (see R.15 below).

R.15: Assumed 1D reference interior model for Mars. Model is constrained by Mars's degree-2 tidal deformation, Moment of Inertia, Mean density, and seismic wave arrival times Stähler et al. (2021)

Radius (km)	Density (kg/m³)	Bulk Modulus (GPa)	Shear Modulus (GPa)
0 – 1830	6200	142.8	0.0
1830 – 3340	3600	130.2	79.5
3340 – 3390	3300	104.3	61.0

As an additional check (and to more generally probe the impact of our choice of loading Love number on inversion results), we have conducted an additional set of inversions which assume k'_ℓ that are 200% and 0% (relative to baseline values noted above, the '0%' indicates a case with no deformational response to surface loads). Posteriors for these inversions are shown in R.18 below, and indicate that changes in assumed loading Love numbers induce shifts of, at most, $\sim$5% relative to the results of nominal inversions. In other words, our choice of loading Love number (i.e., associated with the uncertainty of the assumed 1D model, which is much smaller than 100%) has an effectively negligible effect on inversions results.

In any case, we agree with the reviewer that these methods and the impact of assumed k'_ℓ can be more clearly described in the manuscript. We have therefore made the following changes:

→ We have revised lines 619-622 in the methodology to describe the methods used to compute loading Love numbers, including the semi-analytic approach of Wagner et al. 2024, and mention of the assumed interior model.

→ We have added text noting the adopted loading Love numbers ($k_2' = -0.14126$ and $k_3' = -0.09109$) in line 622.

→ We incorporate discussion of varying k_ℓ' on our inversion results in lines 629-633 of the methodology.

→ We incorporate results associated with varying k_ℓ' by 200% and 0% relative to baseline values into Extended Data Figure 9 of the revised manuscript.

Atmosphere

Our gravity field estimation follows the methodology of Genova et al. (2016), in which the contributions due to the atmosphere are estimated prior to solving for time-variable gravity coefficients associated with the solid body response. Genova et al. (2016) derived these coefficients from surface pressure grids from the Mars Global Reference Atmospheric Model (Mars-GRAM 2010; Justh (2014)), at a 2-hour cadence (see also our earlier discussion in response to the reviewer's Comment 2). Genova et al. (2016) found that the key variables that impact predicted pressure field (and therefore the atmospheric gravity correction) include: (i) the solar flux (ii) the level of atmospheric dust opacity (background or dust storm conditions). Genova et al. (2016) assessed the sensitivity of the gravity solution to (i) and (ii) and find that that whereas the incident solar flux has an effectively monotonic impact on gravity coefficients, dust opacity tends to amplify the impact of changes in incident solar flux on Mars's time-variable gravity coefficients. To verify, we have re-computed these variations for two endmember cases for comparison to our nominal atmosphere (i.e., an average solar flux scenario, 'Avg EUV', $F_{10.7} \approx 130$ sfu): (1) a cold atmosphere case with minimum solar flux ($F_{10.7} \approx 70$ sfu or solar flux units where 1 sfu $= 10^{-22}$ W m^{-2} Hz^{-1}) combined with dust-storm conditions (column-integrated visible optical depth $\tau \gtrsim 5$ Madeleine et al. (2011)) or 'Cold min EUV dust', and (2) a warm atmosphere case with maximum solar flux ($F_{10.7} \approx 200$ sfu) combined with the same dust opacity or 'Warm max EUV dust' (models are extracted from Millour et al. (n.d.)). We find that variations in the retrieved $\Delta C_{3m}^{A,B}$ and $\Delta S_{3m}^{A,B}$ across these permutations are stable across multi-year timescales (see R.16 below) and are generally smaller than uncertainties presented in Table 1 and Extended Data Table 1 in the revised manuscript (see also R.17 below).

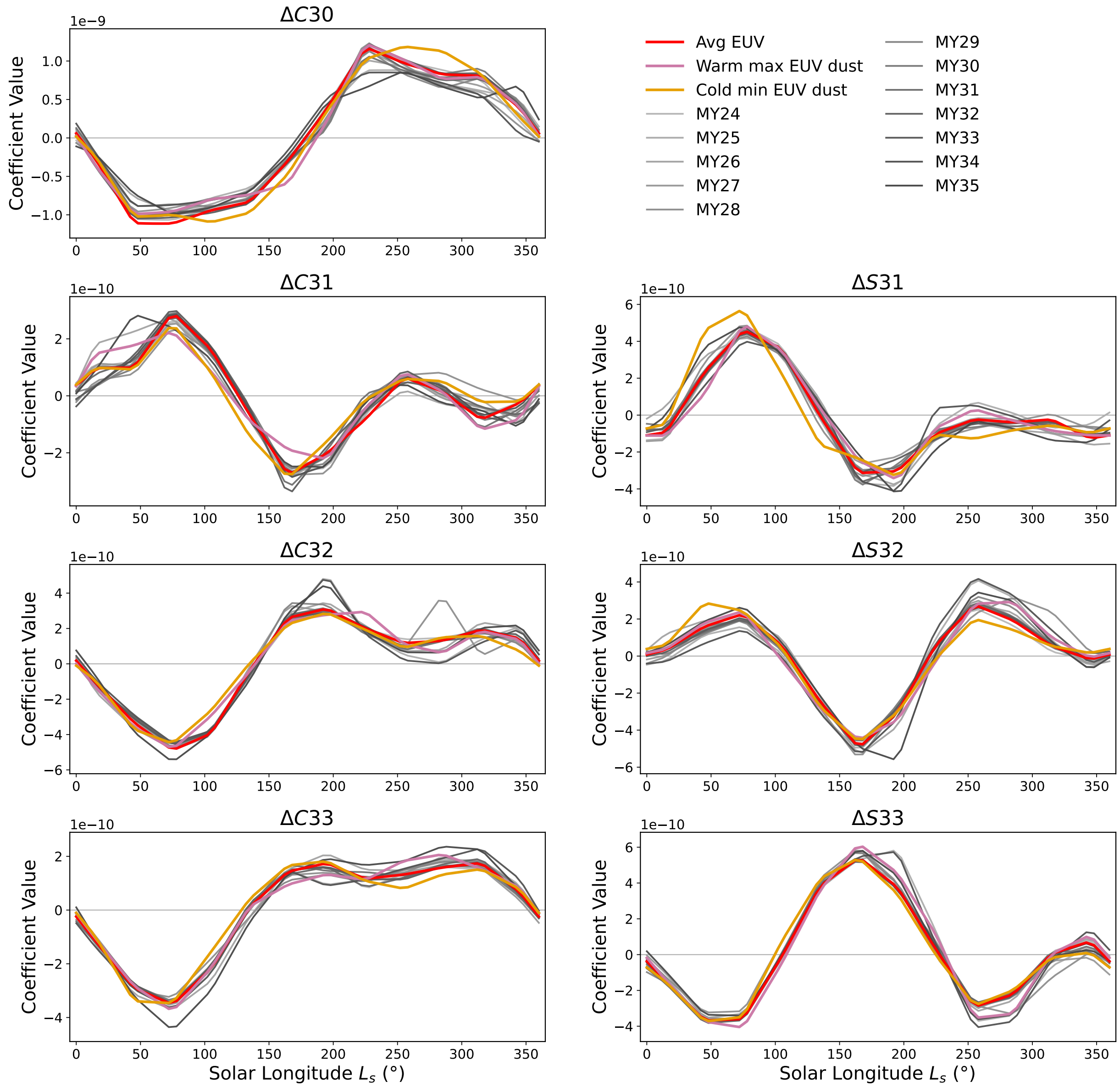

R.16: Seasonal evolution of degree-3 gravity coefficients across models for Mars's atmosphere. Curves show the 4π-normalized, demeaned seasonal time series of ΔC_{30}, ΔC_{31}, ΔS_{31}, ΔC_{32}, ΔS_{32}, ΔC_{33}, and ΔS_{33} as a function of solar longitude L_s. Colored curves denote the average-extreme ultraviolet (EUV) (red), warm/max-EUV with dust (pink), and cold/min-EUV with dust (orange) atmospheric scenarios, while gray curves show the individual realizations of the Avg EUV model from MY24 through MY35. Horizontal gray lines mark zero amplitude in each panel.

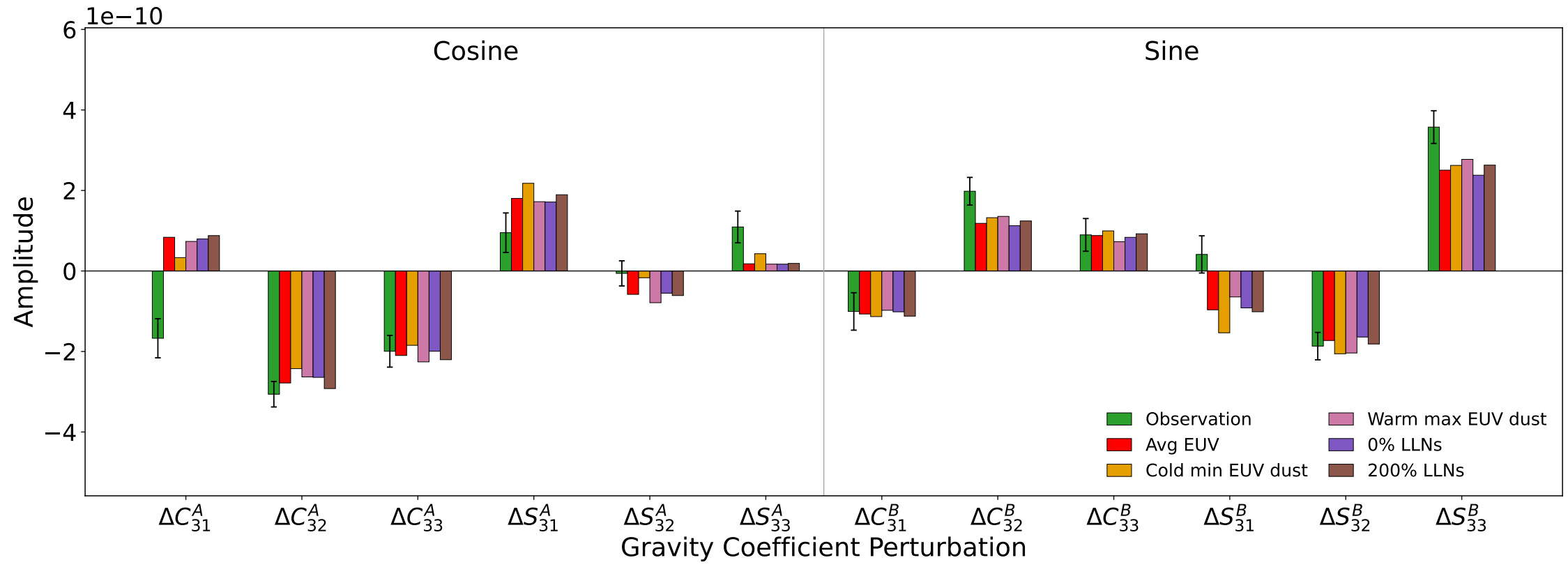

R.17: Sensitivity of the degree-3 gravity signals to atmospheric models and assumed loading Love numbers. Bars show the total observed seasonal degree-3 perturbations (green), the nominal atmospheric correction using the average-EUV scenario (red), atmospheric corrections for the cold/minimum-EUV/dust and warm/maximum-EUV/dust endmember scenarios (orange and pink), and cases with 0% and 200% the loading Love numbers k'_ℓ assigned to the nominal Average EUV atmosphere (purple and brown, see Equation 3 of the revised text). The left and right groups show the cosine and sine terms, respectively. Error bars on the observations denote 15× formal uncertainties.

As an additional check, we have directly computed the predicted impact of variations in (i) - (ii) above and re-run tidal tomography inversions (adjusting the solid body tidal response to account for differences between each of these models, including at degree-2). The figure below shows the resulting posteriors for recovered degree-1 variations in shear modulus (analogous to panels a–c of Fig. 2 of the manuscript; see R.18 below):

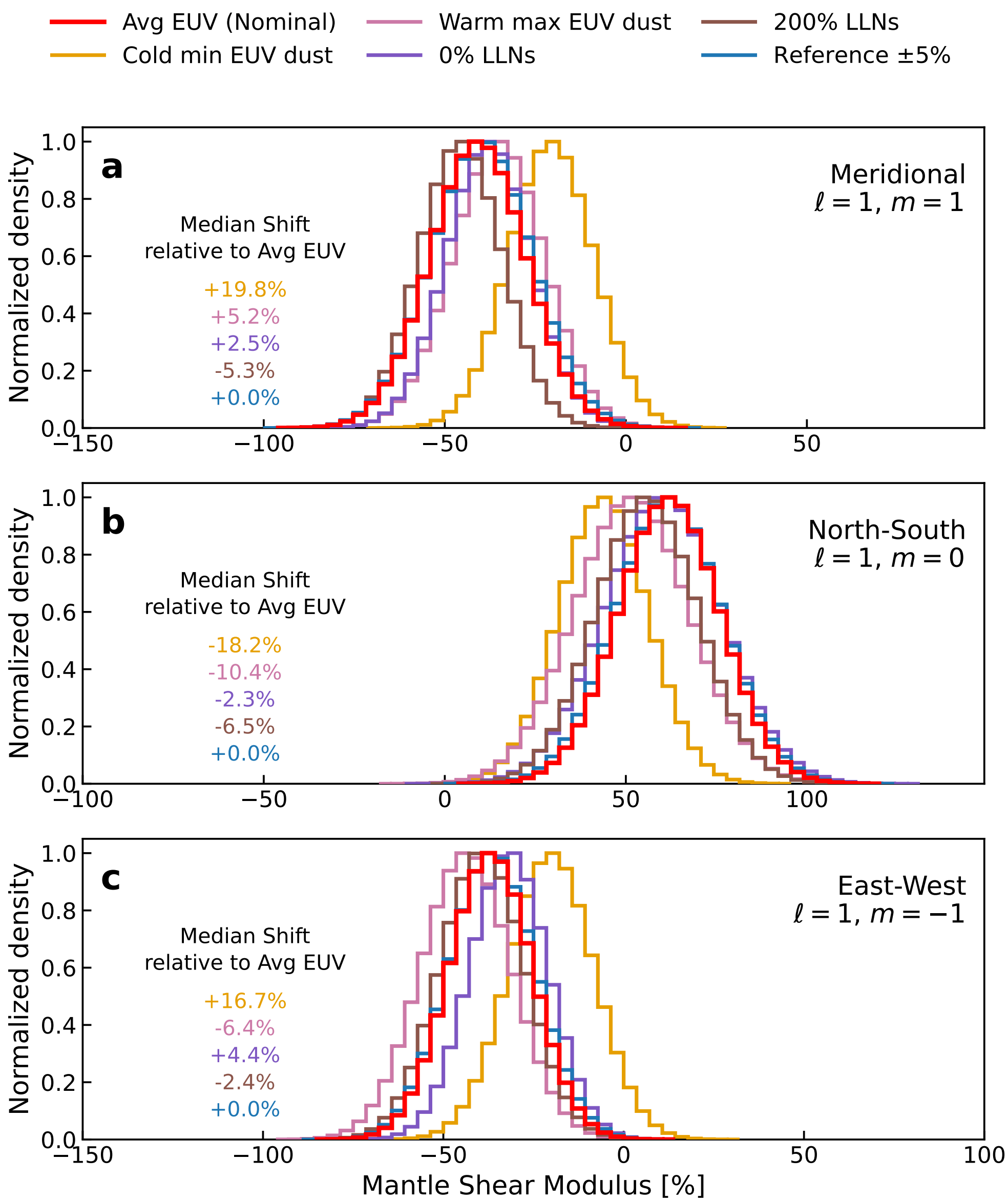

R.18: Sensitivity of tidal tomography results to atmospheric models, assumed loading Love numbers, and the choice of reference model. Posterior distributions of the degree-1 mantle shear-modulus coefficients for **(a)** the meridional $\ell = 1$, $m = 1$ term, **(b)** the north–south $\ell = 1$, $m = 0$ term, and **(c)** the east–west $\ell = 1$, $m = -1$ term for scenarios of the nominal atmospheric correction using the average-EUV scenario (red), atmospheric corrections for the cold/minimum-EUV/dust and warm/maximum-EUV/dust endmember scenarios (orange and pink), cases with 0% and 200% the loading Love numbers k'_ℓ assigned to the nominal Average EUV atmosphere (purple and brown, see Equation 3 of the revised text), and a case where the reference shear and bulk moduli of the crust and mantle are sampled from a Gaussian distribution centered about nominal values (see Table 1 of the revised manuscript) and a standard deviation equal to 5% of these values (light blue). Median shifts for each scenario relative to the average EUV case is shown to the left in each plot.

Based on the above, we find that the change in posteriors across atmosphere models is up to 20% of their median values (with largest shifts occurring for the 'Cold min EUV dust' case). These changes are certainly substantial, though, recovered posteriors indicate north-south patterns which still align with the crustal dichotomy (degree-1 order-0 variations remain significant at the 3-sigma level, and degree-1 order-1 variations remain significant at the 2-sigma level).

We agree with the reviewer that this sensitivity analysis should be incorporated into the manuscript. We have therefore made the following changes:

→ We have incorporated additional text in the Methodology section ('Gravity Field Inversion', lines 608-674) describing our procedure for correcting for Mars's atmosphere. In this added text (lines 654-674), we discuss the impact of different assumed atmospheric models on the time-variable gravity coefficients presented in Table 1 and Extended Data Table 1 as well as the results presented in Fig. 2 of the revised manuscript. In the added text, (line 669), we also note that different atmospheric models produce shifts of up to 20% in inferred shear modulus asymmetries.

→ We have included incorporated R.16, R.17, and R.18 above as Extended Data Figs. 7, 8, and 9.

→ We have added $1-\sigma$ uncertainties to red bars (i.e., for the atmosphere) in Fig. 1a based on inter-annual variations in the gravity field predicted by the Avg EUV model.

We also note that this comment overlaps substantially with comments raised by Referee #1 regarding the impact of the atmosphere on our inversion results. As such, much of the above discussion (and changes to the manuscript) are very similar to those mentioned in response to Referee #1's comments.

Comment 5.

> *I also find the data presentation problematic, as it compares observed data after atmospheric correction to the seasonal tidal response. It would be more relevant to graphically represent the total amplitude inferred from the observation and the contribution of each component, in order to explain the overall signal, including both observation and modeling uncertainties.*

Response: Thank you for this suggestion. We agree with the reviewer that it is more informative to show the *total* observed time-variable gravity signal alongside the individual modelled contributions (and their uncertainties), so that the relative magnitudes of the atmospheric and seasonal tidal contributions can be compared with each other. We have therefore made the following change:

→ We have added bars to Figure 1a of the revised manuscript to show the full time-variable gravity signal (i.e., uncorrected for the atmosphere), the predicted atmospheric contribution (from the nominal atmospheric model, see discussion above), and the observed (i.e., atmosphere-corrected) observation of the seasonal solid-body tidal response. In addition, to improve presentation of the data, we have incorporated a horizontal dashed line at y = 0 to indicate the prediction for the degree-3 gravity field for a spherically symmetric Mars with no atmosphere.

We note that the updated Fig. 1 of the manuscript also now incorporates both modelling and observational uncertainties for each set of bars (see response and changes to the manuscript made

in response to Comment 4 above).

Comment 6.

> *The assumption of a constant load Love number, not taking into account the effect of lateral heterogeneity (l. 506-509), should also be further justified, and some tests using the LOV3D code should be carried out to support this assumption. Overall, a more careful quantification and discussion of the different components contributing to the observed time-varying signal should be done, in order to make the interpretation in terms of seasonal tidal response more robust.*

Response: Thank you for this comment. We agree that the assumption of a 1D model when deriving the loading Love numbers we adopt for the atmospheric correction should be better justified, especially given our inference of $> 20\%$ degree-1 variations in mantle shear modulus. A useful starting point is to distinguish two qualitatively different impacts of 3D structure to variations on the loading love numbers:

→ the *within-harmonic* correction, i.e. a 3D modification of the degree-ℓ' response to a degree-ℓ' load; and

→ the *mode-coupling* correction, i.e. a 3D-induced coupling of a degree-$\ell_0 \neq \ell'$ load into a degree-ℓ' response.

Generally, mode-coupled responses are approximately 10^{-2} or less than the amplitude of forcing. Mode coupled responses therefore become significant only when *forcing* at another (usually higher) degree is much larger than the (within harmonic) forcing at the degree of interest. This is the case for body tides: on Mars, the degree-ℓ tidal potential scales as $W_\ell \propto (R_{\text{Mars}}/r_\odot)^\ell$ with $R_{\text{Mars}}/r_\odot \approx 1.5 \times 10^{-5}$, so that the degree-3 tidal forcing is roughly five orders of magnitude smaller than the degree-2 tidal forcing. In the body-tide problem, mode-coupling of the degree-2 response into a degree-3 response at even the 10^{-4}–10^{-3} level (we find coupling closer to 10^{-3} - 10^{-2}) would therefore exceed the intrinsic degree-3 tidal response by several orders of magnitude.

By contrast, for Mars's atmosphere, surface loads at degree-2 and degree-3 are of *comparable magnitude* — this is visible in the coefficients computed from Mars-GRAM 2010 and MCD surface pressure fields (e.g., Genova et al. (2016)). Any mode-coupling of a degree-2 surface load into a degree-3 response (which, as noted above, is suppressed by an additional factor $\approx 10^{-3} - 10^{-2}$) is therefore overshadowed by the direct within-harmonic response to the load. A similar argument could be applied for polar cap loading (i.e., in this case, the non-zonal within-harmonic degree-3 terms are expected to be approximately 10x larger than mode coupled terms at these harmonics Sanchez et al. (2006)).

The only remaining concern is then the sensitivity of the within-harmonic response of the interior to deep-seated mantle heterogeneities. We note that, at least for body tides, this is usually a small second-oder effect even for interiors large heterogeneity amplitudes (10's of %, see e.g., Fig. 4 of Rovira-Navarro et al. (2024)). As such, for models with $> 20\%$ degree-1 shear-modulus variations recovered in our tidal tomography inversions, we anticipate that the loading Love numbers k_2' and k_3' should differ from values produced by spherically symmetric models by only a small amount. We agree with the reviewer that this argument should also be verified numerically, however, we note that the LOV3D code of Rovira-Navarro et al. (2024) is currently restricted to computing

body tides and does not support surface loading, so it cannot be used to check the loading Love numbers directly. We therefore instead use an in-house 3D finite-element code that was used to benchmark LOV3D (following the methodology of Berne et al. (2023a), Berne et al. (2023b), Berne et al. (2024), and Berne et al. (2026)) and supports surface loading for 3D bodies. In brief, the code solves Poisson's equation and the elasto-gravitational equation of motion for a laterally heterogeneous, non-rotating, non-translating planetary body on an unstructured tetrahedral mesh with ∼150,000 nodes; the reference 1D background model is identical to that assumed for tidal tomography inversions, and the imposed 3D structure is the maximum a-posteriori model from our tidal tomography inversion (degree-1, order-$(-1, 0, 1)$ shear-modulus variations of 36%, 60%, 42% in the mantle). For input forcings, we apply degree-2 and degree-3 fields across each spherical harmonic order, and average the resultant responses per degree.

The results for the degree-2 and degree-3 loading Love number derived from numerical models and that assumed as inputs into calculations are shown in the Table below. Generally, we find good agreement (<1% differences) between our result and input loading Love numbers:

R.19: Comparison of assumed and predicted Love numbers. The absolute difference is |Predicted − Assumed|, and the relative difference is expressed as a percentage of the assumed value.

Quantity	Assumed	Predicted	Abs. difference	Rel. difference (%)
k'_3	-0.09109	-0.09113	4.0×10^{-5}	0.044%
k'_2	-0.14126	-0.14127	1.0×10^{-5}	0.0071%

Aside from the above, we also note that we have carried out sensitivity tests of our tidal tomography solution to the assumed values of our loading Love numbers and find these changes to be effectively negligible (see e.g., R.18 above).

Based on the reviewer's comment, we have made the following changes to the manuscript:

→ We have added text near lines 619-629 justifying the use of spherically symmetric models for computing loading Love numbers used in the work. We summarize the physical argument (mode coupling is negligible because atmospheric loads at degrees 2 and 3 are comparable in magnitude) and note that 3D finite-element calculations reproduce the adopted values to better than 1%.

We note that this comment overlaps substantially with Referee #1's comment 5b on our derivation of loading Love numbers, and so our response and associated manuscript changes draw on much of the same underlying analysis (including the values in the above Table).

Comment 7.

> *The lateral variations in the mantle's effective shear modulus (> 40%) required to explain the seasonal tidal response at degree 3 appears very large. While heterogeneities of melt distribution between the two hemispheres appear a reasonable explanation, the minimum inferred melt fraction of 2%, considered as their favored explanation (l. 176-179), seems rather low. According to Schmeling (1985), for example, except in very specific melt configurations, it is difficult to reduce the shear modulus to this extent*

with only 2%. The inferred shear modulus variations instead indicate larger variations in melt fraction between the two hemispheres, which is more difficult to explain. The authors compared their inferred values to typical values of the melt content expected in the asthenosphere beneath Earth's mid-oceanic ridges to justify the typical range inferred in their study. But it must be kept in mind that, in the terrestrial context, this melt fraction is achieved very locally in specific geodynamic contexts, whereas here, they need such high values on a global scale in the mantle, and not only in the asthenosphere, to explain the complex 3D tidal response. A more careful discussion of the link between the inferred shear modulus variations, the melt distribution and the geodynamic context is needed.

Response: Thank you for this comment. We agree with the reviewer that the original manuscript's interpretation of the inferred degree-1 shear-modulus asymmetry in terms of a $\geq$2% through-going partial melt fraction is difficult to reconcile with existing experimental and theoretical literature, and that a more careful discussion/analysis is needed. Based on this comment, we have substantially revised the interpretation of our tidal tomography result, moving away from an explanation that relies on widespread partial melt and toward one that invokes crystalline temperature variations. We address the reviewer's comment in two parts below: Part 1 discusses the revised physical interpretation of the shear modulus asymmetries in Mars's mantle, and Part 2 addresses the framing of this result in terms of predictions for the geodynamics of the Martian mantle.

Part 1: A revised interpretation of mantle shear modulus asymmetries.

We agree with the reviewer that invoking a $\geq$2% through-going melt fraction as the preferred explanation for our inferred shear-modulus asymmetry is problematic for several reasons:

1. First, as noted by the reviewer, the experimental work of Schmeling (1985) indicates that (except in very specific melt geometries e.g., highly interconnected media) a 2% melt fraction is insufficient to produce the $\gtrsim 20\%$ reduction in effective shear modulus inferred from our tidal tomography inversions. Reproducing our inferred asymmetry with a plausible melt geometry would therefore demand a larger (and more implausible) melt fraction than the value we report.
2. Second, and as also pointed out in a related comment by Referee #1, the high pressures expected throughout the mantle (especially in the lower portions) raise the temperature of the peridotite solidus and make a through-going (i.e., extending from beneath the lithosphere all the way to the CMB) partial melt layer difficult to sustain.
3. Third, existing geodynamical models of Mars (e.g., 3D convection models of Plesa et al. (2018) and Plesa et al. (2021) and the parameterized convection models of Thiriet et al. (2018) and Gibet et al. (2022)) do *not* predict widespread melt throughout the entire Southern Hemisphere mantle. These models instead produce (at most) localized, sub lithospheric melting beneath regions of thickened crust (e.g., Tharsis, Elysium), together with elevated mean mantle temperatures beneath the Southern Hemisphere.
4. Finally, we also agree with the reviewer that our original analogy invoking asthenospheric melt fractions inferred beneath Earth's mid-ocean ridges is misleading: those melt fractions are achieved locally in a very specific geodynamic setting (decompression melting beneath spreading centres) and cannot straightforwardly be extrapolated to the entirety of Mars's mantle depth range.

In light of these arguments, we agree with the reviewer that widespread through-going partial melt should not be our preferred explanation for the observed asymmetry. In seeking a better physical explanation for observed shear modulus asymmetries, we have re-examined the literature on the relationship between shear modulus and crystalline temperature in mantle rocks. In the original version of the manuscript, we had dismissed a purely thermal explanation on the grounds that the standard $d\mu/dT$ derived from experiments at seismic periods on olivine would require an implausibly large ($\gtrsim 1000$ K) temperature contrast to reproduce our inferred shear modulus differences. However, as highlighted by Jackson and Faul (2010), the effective shear modulus of olivine at long forcing periods (i.e., periods much longer than seismic timescales, such as the Martian annual period of 5.94×10^7 s) is substantially more sensitive to temperature than at seismic periods. This is because, at long forcing periods, anelastic relaxation mechanisms (e.g., grain-boundary sliding, diffusion creep, which are strongly temperature dependent; Faul and Jackson (2015)) contribute significantly to the effective shear modulus.

Based on the above, we have extracted the experimental data from Jackson and Faul (2010) shear modulus vs forcing timescale curves evaluated at different temperatures (specifically the 'background' Burgers model in Figure 1a of that work), fit each temperature curve with a power law function, and extended these fits to the Martian annual period (5.94×10^7 s). The resulting values are shown below in R.20.

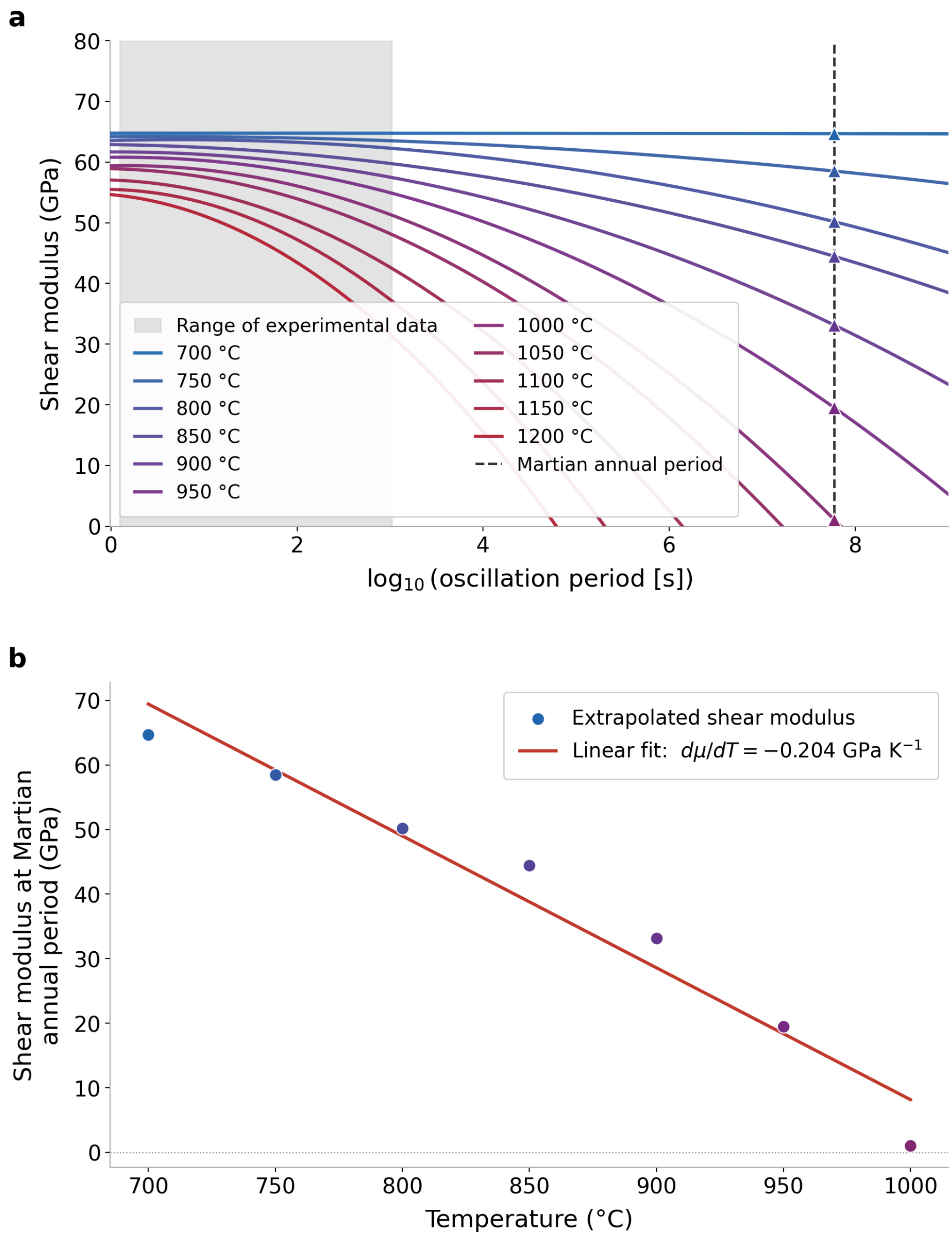

R.20: Sensitivity of olivine shear modulus to temperature at the Martian tidal period. (a) Shear modulus versus oscillation period for the background-only Burgers model of olivine rheology evaluated from experiments on crystals at temperatures between 700–1200°C (see Fig. 1a of Jackson and Faul (2010)). Colored curves are power-law fits extended beyond the range of experimental data (gray shading) to the Martian annual period (5.94×10^7 s; dashed vertical line). **(b)** Shear moduli evaluated at the annual period as a function of temperature. A linear trendline (red) yields $d\mu/dT \approx -0.204$ GPa K^{-1} which is used to recover hemispheric variations in temperature from shear modulus asymmetries (see Fig. 3 of the revised manuscript).

From these curves, we estimate an approximate (linear) transfer function between shear modulus and temperature change at the Martian annual period of

$$\frac{d\mu}{dT} \approx -0.204 \text{ GPa K}^{-1}.$$

Which would require a temperature contrast of approximately:

$$\Delta T \sim \frac{\Delta\mu}{|d\mu/dT|} \approx 200\text{–}400 \text{ K}.$$

to explain our tidal tomography result (see updated Fig. 3 of the manuscript, also R.21 below). We note that several anelastic laws have been published (e.g., Yamauchi and Takei 2016; McCarthy et al. 2011), but we have chosen that of Jackson and Faul 2010 as it has been used in several analogous applications for the Moon (Nimmo et al. 2012), Mars (Nimmo and Faul 2013), and the Earth (Dalton and Faul 2010; Lau and Faul 2019). Moreover, an explanation for mantle asymmetries based on a few hundred Kelvin temperature differences should be more consistent with existing literature on Mars's geodynamics (e.g., Gibet et al. (2022)) than an explanation that involves through-going partial melt. For example, a crystalline temperature anomaly is more consistent with the observations described in Figure 3 of Plesa et al. (2016) (panel f) as well as Sun and Tkalčić (2024) which describe differences in seismic wave attenuation between the Northern and Southern Highlands mantle (but do not find attenuation values which would indicate widespread partial melt in the Southern highlands).

In addition to this temperature anomaly, we infer a potential iron enrichment of up to 5% over the Southern highlands mantle (we believe it is more fitting to consider iron enrichment (Fayalite-Forsterite Mol fraction difference) instead of Pyroxene to Olivine depletion since the former is expected to drive larger variations in COM-COF offset). As before, the inferred range of iron enrichment is derived by comparing the COM-COF offset to shear modulus asymmetries from our tidal tomography inversion. Notably, we would require COM-COF offsets that are approximately $50\times$ observed values Smith et al. (2001) if we invoked iron enrichments in isolation to explain shear modulus anomalies (see R.21 below, similar arguments could be made for other compositional variations e.g., water content), suggesting that inferred asymmetries are indeed largely thermal and not compositional.

Even if our results do not support a through-going partial melt anomaly, we note that the relatively elevated temperatures expected beneath the Southern highlands would make localized melting in this region (e.g., below the lithosphere) more likely than the Northern lowlands (as also noted by the reviewer). In principle, such a thermal state might be expected to enhance volcanism in the Southern hemisphere. This expectation is paradoxical in light of observations, which instead indicate more prominent young volcanic activity in the North, as discussed in lines 247–256 of the manuscript.

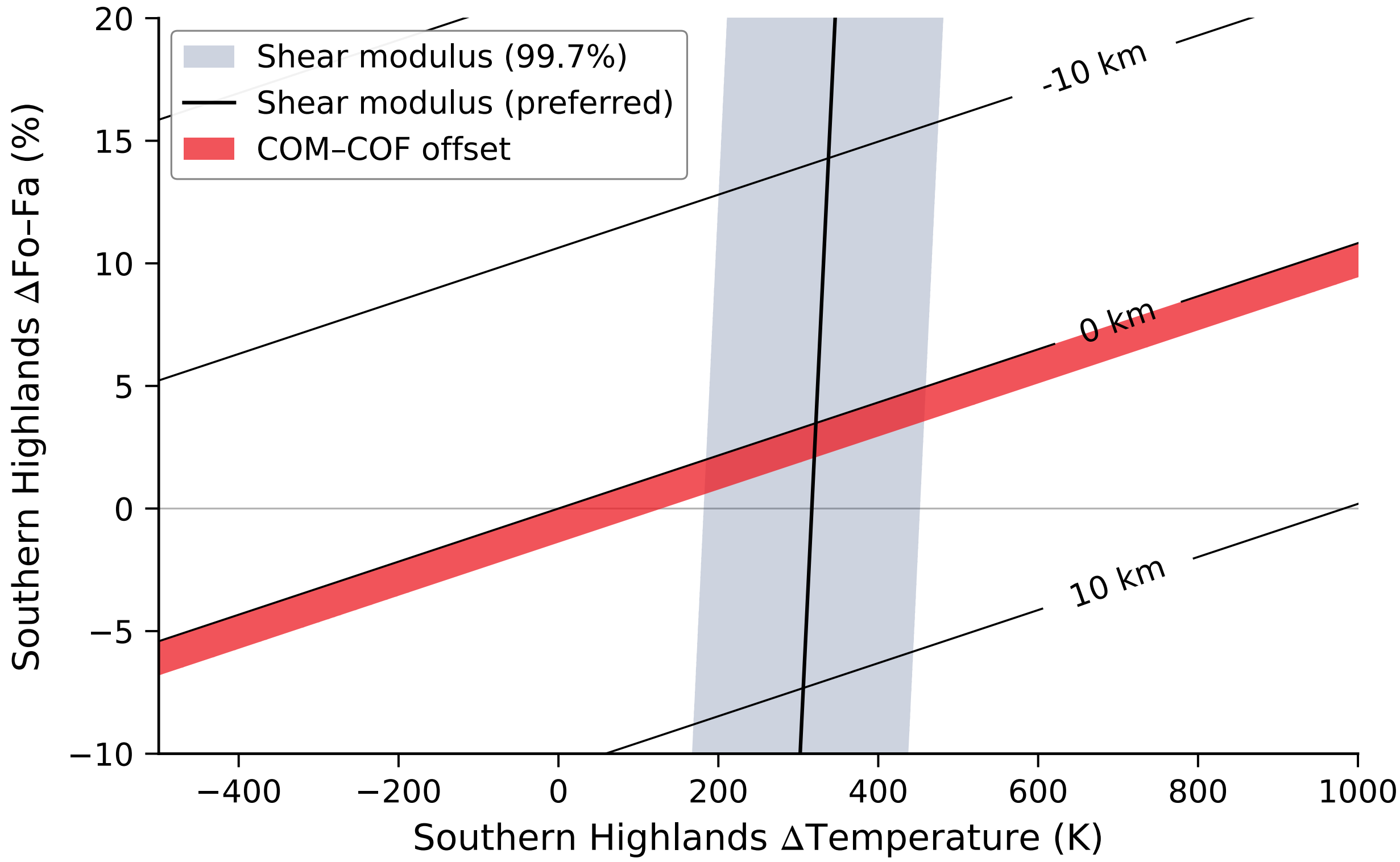

R.21: Relationship between temperature, composition, and inferred shear modulus variations in the Martian mantle. Shaded regions indicate the constrained ranges of the inferred Martian effective hemispheric shear modulus variation and the impact of mantle structure on the center-of-mass center-of-figure (COM–COF) offset as a function of Southern Highlands mantle temperature anomaly and fayalite–forsterite mol fraction contrast (Fa–Fo) (for details regarding calculations, see Methods). Values are projected onto a degree-1 pattern with poles centered at 45°N 138°W (Northern lowlands) and 45°S 42°E (Southern highlands). Black contours indicate COM-COF offsets at 10 km intervals. Since the COM–COF offset is sensitive to the structure of both the crust and mantle, our modelled 0–1.31 km range of values accounts for the possibility of (i) an anomalous COF that is fully attributable elevated topography over the Southern highlands (paired with Airy or Pratt compensation of this structure at depth) or an anomalous COM arising from a relatively dense crust over the Northern Lowlands (i.e., the 0 km bound) Smith et al. (2001), Wieczorek and Zuber (2004), and Wieczorek et al. (2022), (ii) an anomalous COM that is solely attributable to mantle density variations (i.e., the 1.31 km bound) and (iii) intermediate cases. The blue shaded area and thick solid black line respectively denote the 99.7% confidence bounds and preferred value for the overall shear modulus difference of $81 \pm 60\%$ inferred from gravity data in this work (see Fig. 2 of the revised text). By identifying overlapping portions of parameter space that satisfy both the 99.7% range of inferred shear modulus variation and COM–COF offset, we infer that hemispheric asymmetries can be explained by a temperature anomaly of 200–400 K and an iron enrichment of up to 5% in the Southern Highlands mantle.

Based on this comment, we have made the following changes:

- → We have changed the title of the paper to "Tidal Tomography Reveals a Thermal Anomaly Beneath Mars's Crustal Dichotomy"
- → We have heavily updated Figure 3 (which previously involved solving for the extent of Pyroxene depletion and extent of partial melting in the Southern highlands mantle using observed shear modulus and COM-COF offset). We have changed this to instead more directly infer iron content variations (i.e., ΔFa-Fo or change in fayalite to forsterite mol fraction) vs. temperature variations. Based on this Figure, we claim the existence of a temperature variation of approximately 200–400 K and ΔFa-Fo mol fraction of up to 5% over the Southern highlands mantle. Figure 3 also contains contour lines for the COM-COF offset to better support the argument that inferred compositional anomalies are relatively small.
- → We have heavily modified lines 183-202 to refer to the updated result (i.e., the inference of temperature variations of 200–400 K and compositional variations of up to 5%). In this modified paragraph, we discuss our logic for using the COM-COF offset to constrain the compositional component of inferred asymmetries.
- → We have changed the title of the section "A Partially Molten Mantle Beneath Mars's Southern Highlands" to "A Thermal Anomaly Beneath Mars's Southern Highlands".
- → We have edited Figure 4 to remove the depiction of through-going partial melt in Mars's mantle. Even so, we have kept the "Melt Ascent" in the crust in line with the prediction that there should be elevated amounts of partial melt in this region if this Southern highlands mantle is warmer than that in the north (though, we have changed the label associated with this feature within the Figure).
- → We have incorporated the Figure shown above (i.e., indicating the relationship between shear modulus and timescale for different temperature curves from Jackson and Faul (2010)) as Extended Data Fig. 5.
- → We have heavily modified the "Modeling Partial Melt and Pyroxene Depletion" (now titled "Modeling Temperature and Iron Content") to include discussion of our new method for calculating a transfer function between temperature and shear modulus variations (and other quantities). In this section we refer to the added Extended Data Fig. 5.
- → We have made numerous small edits throughout the manuscript, including to the abstract, the "Martian Volcanism and Future Measurements" section, and the Methods to accommodate the new result focused on 200–400 K temperature variations as an explanation for shear modulus variations.

Part 2: Framing results in a broader geodynamic context.

We also agree with the reviewer that the inferred thermal asymmetry should be framed more carefully in the context of literature on the geodynamics of the Martian interior (e.g., existing hypotheses for the formation of the Martian crustal dichotomy). Three classes of hypotheses for the formation of the crustal dichotomy include (i) a hemisphere-scale impact, (ii) spontaneous degree-1 mantle upwelling/convection, and (iii) thermal insulation of the Southern Hemisphere mantle by a thick Southern Highlands crust. These hypotheses are not mutually exclusive, but they do make different predictions for the structure of the Martian mantle. To make these predictions explicit, we have compiled them in R.22 below which indicates whether each process is expected to produce a predominantly *thermal* (**T**) or *compositional* (**C**) asymmetry in the present-day mantle.

R.22: Possible mechanisms contributing to the Martian crustal dichotomy and their expected mantle signatures. The "Present-Day Structure" column indicates whether a given process is expected to produce a present-day thermal (**T**) and/or compositional (**C**) asymmetry.

**Process**	**Present-Day Structure**	**Notes**
Giant impact	**C**	Shock melting may generate a substantial compositional heterogeneity in the mantle (Marinova et al. (2008), Reese et al. (2011), Cheng et al. (2024), and Shaw (1970)), but is not expected (in isolation) to result in long-lived secular temperature anomalies of several hundred Kelvin in a given hemisphere (see e.g., Fig. S2 of Cheng et al. 2024).
Spontaneous degree-1 upwelling	**T**	Predicts a warmer mantle beneath the upwelling hemisphere; secondary compositional variations may arise through sporadic decompression melting Zhong and Zuber (2001) and Roberts and Zhong (2006).
Southern-crust insulation / radiogenic heating	**T**	Thick, heat-producing Southern Highlands crust suppresses heat loss and promotes long-lived mantle warming; secondary compositional differences may develop through sporadic decompression melting Plesa et al. (2018) and Gibet et al. (2022).

As summarized in R.22 above, the main distinction among different scenarios for the evolution of the crustal dichotomy is whether they are expected to produce a predominantly thermal or compositional present-day mantle asymmetry. Our preferred model indicates a 200–400 K temperature anomaly beneath the Southern Highlands, accompanied by, at most, only a modest compositional component (i.e., Fo-Fa mol fraction variation or iron enrichment between 0 and 5%; see Fig. 3 of the revised manuscript and R.21). This pattern is, at first glance, more consistent with spontaneous degree-1 upwelling and/or thermal insulation by the thick Southern Highlands crust, both of which predict a primarily thermal mantle anomaly (Zhong and Zuber (2001), Roberts and Zhong (2006), Plesa et al. (2018), and Gibet et al. (2022)). By contrast, a giant impact alone may produce a large accompanying compositional heterogeneity in the mantle but is not expected to generate the inferred long-lived, thermal anomaly of several hundred Kelvin over a single hemisphere (e.g., Cheng et al. (2024), Fig. S2). However, we also expect that scenarios involving degree-1 upwelling or thermal insulation would tend to *deplete* the Southern Highlands mantle in iron (opposite the enrichment we infer). A hybrid scenario could reconcile this discrepancy. For example, an early Northern hemisphere impact may initially (relatively) enrich the Southern Highlands mantle in iron (Reese et al. (2011)) and could also trigger the subsequent thermal insulation or degree-1 upwelling (Citron et al. (2018)) in the antipodal Southern hemisphere. This process would produce a dominantly thermal anomaly with only a small residual (<5% Fo-Fa) compositional component, consistent with our result.

Based on the reviewer's comment, we have made the following modifications:

→ We have heavily revised the second paragraph of the section "A Thermal Anomaly Beneath Mars's Southern Highlands" (lines 203-219) to more explicitly connect our tidal tomography result to hypotheses for the formation of the crustal dichotomy. In particular, the revised text now notes that an impact is not expected to produce a long-lived thermal anomaly over one hemisphere (in isolation), and that spontaneous degree-1 upwelling and Southern Highlands thermal insulation is expected to deplete (not enrich) this region in iron (opposite observations). We then state that our observations can be explained by a hybrid scenario (i.e., an impact over the Northern hemisphere followed by thermal insulation or degree-1 upwelling below the Southern Highlands mantle).

→ To more directly highlight the hypotheses related to thermal insulation by a thick Southern highlands crust on Mars, we have altered language in the introduction (lines 55 - 66) of the manuscript discussing this hypothesis (and citing Gibet et al. (2022) and Plesa et al. (2018)) and its relationship to predictions from hypotheses involving a hemisphere-scale impact and degree-1 convection.

We note that this comment overlaps substantially with several comments raised by Referee #1 (in particular, those related to the plausibility of a through going partial melt layer and the link between the inferred shear-modulus asymmetry and existing hypotheses for the crustal dichotomy). Our response and associated manuscript changes accordingly draw on much of the same underlying analysis.

> *Overall, I find this study very interesting and promising; however, the study lacks modeling details and sensitivity tests to concretely demonstrate the contribution of the seasonal tidal response to the observed time-varying, degree-three gravity field. The interpretation in term of melt variations between the two hemispheres also required further testing and discussions.*

Response: We thank the reviewer for this positive assessment and for their detailed and constructive comments, and appreciate the time and care they devoted to evaluating the work. We hope that the changes made to our analysis and text address the reviewer's concerns and improve the clarity and strength of the manuscript.

References (Referee #2)

Berne, Alexander et al. (2026). "[In preparation: Berne et al. 2026]". In: *Manuscript in preparation*. Placeholder entry — update with full reference once available.

Berne, Alexander et al. (2023a). "Inferring the mean thickness of the outer ice shell of Enceladus from diurnal crustal deformation". In: *Journal of Geophysical Research: Planets* 128.6, e2022JE007712. DOI: 10.1029/2022JE007712.

— (2023b). "Using tidally-driven elastic strains to infer regional variations in crustal thickness at Enceladus". In: *Geophysical Research Letters* 50.—, pp. 311–318. DOI: 10.1029/2023GL106656.

Berne, Alexander et al. (2024). "Jet activity on Enceladus linked to tidally driven strike-slip motion along tiger stripes". In: *Nature Geoscience* 17, pp. 385–391. DOI: 10.1038/s41561-024-01418-0.

Cheng, Kar Wai et al. (2024). "Mars's Crustal and Volcanic Structure Explained by Southern Giant Impact and Resulting Mantle Depletion". In: *Geophysical Research Letters*. DOI: 10.1029/2023GL105910.

Citron, Robert I., Michael Manga, and Eh Tan (2018). "A hybrid origin of the Martian crustal dichotomy: Degree-1 convection antipodal to a giant impact". In: *Earth and Planetary Science Letters* 491, pp. 58–66. DOI: 10.1016/j.epsl.2018.03.031.

Dalton, Colleen A. and Ulrich H. Faul (2010). "The oceanic and cratonic upper mantle: Clues from joint interpretation of global velocity and attenuation models". In: *Lithos* 120.1–2, pp. 160–172. DOI: 10.1016/j.lithos.2010.08.020.

Faul, Ulrich H. and Ian Jackson (2015). "Transient Creep and Strain Energy Dissipation: An Experimental Perspective". In: *Annual Review of Earth and Planetary Sciences* 43, pp. 541–569. DOI: 10.1146/annurev-earth-060313-054732.

Genova, A. et al. (July 2016). "Seasonal and static gravity field of Mars from MGS, Mars Odyssey and MRO radio science". In: *Icarus* 272, pp. 228–245. DOI: 10.1016/j.icarus.2016.02.050.

Gibet, Valentin Bonnet et al. (2022). "A Positive Feedback Between Crustal Thickness and Melt Extraction for the Origin of the Martian Dichotomy". In: *Journal of Geophysical Research: Planets* 127.12, e2022JE007472. DOI: 10.1029/2022JE007472.

Jackson, Ian and Ulrich H. Faul (2010). "Grainsize-sensitive viscoelastic relaxation in olivine: Towards a robust laboratory-based model for seismological application". In: *Physics of the Earth and Planetary Interiors* 183.1–2, pp. 151–163. DOI: 10.1016/j.pepi.2010.09.005.

Justh, H. L. (2014). *Mars Global Reference Atmospheric Model 2010 Version: Users Guide*. Tech. rep. NASA/TM-2014-217499. Huntsville, AL: NASA Marshall Space Flight Center.

Lau, Harriet C. P. and Ulrich H. Faul (2019). "Anelasticity from seismic to tidal timescales: Theory and observations". In: *Earth and Planetary Science Letters* 508, pp. 18–29. DOI: 10.1016/j.epsl.2018.12.009.

Madeleine, J.-B. et al. (2011). "Revisiting the radiative impact of dust on Mars using the LMD Global Climate Model". In: *Journal of Geophysical Research: Planets* 116, E11010. DOI: 10.1029/2011JE003855.

Marinova, Margarita M., Oded Aharonson, and Erik Asphaug (2008). "Mega-impact formation of the Mars hemispheric dichotomy". In: *Nature* 453.7199, pp. 1216–1219. DOI: 10.1038/nature07070. URL: https://doi.org/10.1038/nature07070.

McCarthy, Christine, Yasuko Takei, and Tomohiko Hiraga (2011). "Experimental study of attenuation and dispersion over a broad frequency range: 2. The universal scaling of polycrystalline materials". In: *Journal of Geophysical Research: Solid Earth* 116.B9, B09207. DOI: 10.1029/2011JB008384.

Millour, E et al. (n.d.). *THE MARS CLIMATE DATABASE (VERSION 5.3)*. Tech. rep. URL: http://www-mars.lmd.jussieu.fr.

Nimmo, F. and U. H. Faul (2013). "Dissipation at tidal and seismic frequencies in a melt-free, anhydrous Mars". In: *Journal of Geophysical Research: Planets* 118.12, pp. 2558–2569. DOI: 10.1002/2013JE004499.

Nimmo, F., U. H. Faul, and E. J. Garnero (2012). "Dissipation at tidal and seismic frequencies in a melt-free Moon". In: *Journal of Geophysical Research: Planets* 117.E9, E09005. DOI: 10.1029/2012JE004160.

Plesa, Ana C. et al. (2018). "The thermal state and interior structure of Mars". In: *Geophysical Research Letters* 45.22, pp. 12198–12209. DOI: 10.1029/2018GL080728. URL: https://doi.org/10.1029/2018GL080728.

Plesa, Ana-Catalina et al. (2016). "How Large Are Present-Day Heat Flux Variations Across the Surface of Mars?" In: *Journal of Geophysical Research: Planets* 121.12. Verify: cited as "Plesa et al. 2017" in the response text., pp. 2386–2403. DOI: 10.1002/2016JE005126.

Plesa, Ana-Catalina et al. (2021). "Seismic Velocity Variations in a 3D Martian Mantle: Implications for the InSight Measurements". In: *Journal of Geophysical Research: Planets* 126.6, e2020JE006755. DOI: 10.1029/2020JE006755.

Reese, C. C., C. P. Orth, and V. S. Solomatov (2011). "Impact megadomes and the origin of the martian crustal dichotomy". In: *Icarus* 213.2, pp. 433–442. DOI: 10.1016/j.icarus.2011.03.028.

Roberts, James H. and Shijie Zhong (2006). "Degree-1 convection in the Martian mantle and the origin of the hemispheric dichotomy". In: *Journal of Geophysical Research* 111.E06013. DOI: 10.1029/2005JE002668.

Rovira-Navarro, Marc, Isamu Matsuyama, and Alexander Berne (2024). "A Spectral Method to Compute the Tides of Laterally Heterogeneous Bodies". In: *The Planetary Science Journal* 5.5, p. 129. DOI: 10.3847/PSJ/ad381f. URL: https://doi.org/10.3847/PSJ/ad381f.

Sanchez, Braulio V., David D. Rowlands, and Robert M. Haberle (2006). "Variations of Mars' Gravitational Field Based on the NASA/Ames General Circulation Model". In: *Journal of Geophysical Research: Planets* 111.E6, E06010. DOI: 10.1029/2005JE002442. URL: https://doi.org/10.1029/2005JE002442.

Schmeling, Harro (1985). "Numerical Models on the Influence of Partial Melt on Elastic, Anelastic and Electric Properties of Rocks. Part I: Elasticity and Anelasticity". In: *Physics of the Earth and Planetary Interiors* 41.1, pp. 34–57. DOI: 10.1016/0031-9201(85)90100-1.

Shaw, Denis M. (1970). "Trace element fractionation during anatexis". In: *Geochimica et Cosmochimica Acta* 34.2, pp. 237–243. DOI: 10.1016/0016-7037(70)90009-8.

Smith, David E. et al. (2001). "Mars Orbiter Laser Altimeter: Experiment summary after the first year of global mapping of Mars". In: *Journal of Geophysical Research: Planets* 106.E10, pp. 23689–23722. DOI: 10.1029/2000JE001364.

Stähler, Simon C. et al. (2021). "Seismic detection of the Martian core". In: *Science* 373.6553, pp. 443–448. DOI: 10.1126/science.abi7730. URL: https://www.science.org/doi/10.1126/science.abi7730.

Sun, Xia and Hrvoje Tkalčić (2024). "Constraints on the Origin of the Martian Dichotomy From Southern Highlands Marsquakes". In: *Geophysical Research Letters* 51.8, e2024GL110921. DOI: 10.1029/2024GL110921. URL: https://doi.org/10.1029/2024GL110921.

Thiriet, Mélanie et al. (2018). "Hemispheric Dichotomy in Lithospheric Thickness on Mars Caused by Differences in Crustal Structure and Composition". In: *Journal of Geophysical Research: Planets* 123.4, pp. 823–848. DOI: 10.1002/2017JE005431.

Wagner, N. L. et al. (June 2024). "Evaluating the Use of Seasonal Surface Displacements and Time-Variable Gravity to Constrain the Interior of Mars". In: *Journal of Geophysical Research: Planets* 129 (6). ISSN: 2169-9097. DOI: 10.1029/2023JE008053.

Wieczorek, Mark A. and Maria T. Zuber (2004). "Thickness of the Martian crust: Improved constraints from geoid-to-topography ratios". In: *Journal of Geophysical Research: Planets* 109.E1, E01009. DOI: 10.1029/2003JE002153.

Wieczorek, Mark A. et al. (2022). "InSight Constraints on the Global Character of the Martian Crust". In: *Journal of Geophysical Research: Planets* 127.5, e2022JE007298. DOI: 10.1029/2022JE007298. URL: https://doi.org/10.1029/2022JE007298.

Yamauchi, Hatsuki and Yasuko Takei (2016). "Polycrystal anelasticity at near-solidus temperatures". In: *Journal of Geophysical Research: Solid Earth* 121.11, pp. 7790–7820. DOI: 10.1002/2016JB013316.

Zhong, Shijie and Maria T. Zuber (2001). "Degree-1 mantle convection and the crustal dichotomy on Mars". In: *Earth and Planetary Science Letters* 189.1–2, pp. 75–84. DOI: 10.1016/S0012-821X(01)00345-4.

Responses to Referees for:

Tidal Tomography Reveals a Thermal Anomaly Beneath Mars's Crustal Dichotomy

June 24, 2026

Referee #1

> *The authors have provided a thorough and satisfactory revision, particularly regarding their interpretation of the shear module's asymmetry, the change to the title, and the more detailed description of the methodology. Before publishing, however, I would suggest taking the following point into consideration:*

Response: Thank you very much. We have addressed the reviewer's remaining comments (see below).

> *The authors made a thorough investigation of how atmospheric model uncertainties affect the results. However, I find it limiting that these uncertainties are not propagated onto the calibrated $\Delta C/S_{lm}$ coefficients. While I appreciate the addition of the posterior distribution plots of shear modulus variation as a function of the atmospheric model adopted, the uncertainties reported in the manuscript (e.g., Figure 2 and Table 1) may be misleading to non-specialist readers. I suggest that the author explicitly state in the captions of Figure 2 and Table 1 that the uncertainties on the solid-body time-varying gravity coefficients do not account for uncertainty in the atmospheric model used to derive them.*

Response: Thank you for this suggestion. To clarify, we have avoided propagating the shifts resulting from the "Cold min EUV dust", "Warm max EUV dust" atmospheric models into uncertainties on $\Delta C_{lm}^{A,B}$ and $\Delta S_{lm}^{A,B}$ since these models are meant to represent extreme scenarios (i.e., which do not translate cleanly into e.g., $1-\sigma$ bounds). Moreover, the error bars reported in Figure 1 panel a for atmospheric coefficients are calculated using multi-year variations in the gravity field for the "Avg EUV" model. Since these are smaller than the formal uncertainties multiplied by 15 (i.e., the uncertainties on coefficients used in inversions), these are effectively (though implicitly) accounted for in our inversion procedure.

To make these points clearer, and in line with the reviewer's suggestion, we have made the following changes:

→ We have altered the captions of Figure 1 and Table 1 to make it clear that the uncertainties on gravity field coefficients used in inversions are substantially larger than those derived from multi-year variations for our atmospheric model, and therefore implicitly subsume these uncertainties. We additionally note that total uncertainties do not necesarily account for extreme scenarios (e.g., very warm, very cold, or high dust conditions), and refer the reader to the Methods and Extended Data Figure 6 for additional information on the potential impact of these models. Note that we have chosen to alter the caption of Figure 1 (not Figure 2, as the reviewer suggested) since this Figure deals more directly with the gravity field coefficients and their uncertainties than does Figure 2 of the manuscript.

Referee #1 (Remarks on code availability)

The provided code (Love3D_multi) does not allow readers to readily reproduce the results presented in this manuscript. The repository should include a worked example enabling reproduction of the key results. Since providing a full inverse Bayesian pipeline would be impractical, I recommend the authors include a script/example that takes the inferred interior structure (base structure + shear modulus variations) as input and demonstrates that it correctly reproduces the observed $\Delta C/S_{lm}$ values, within uncertainty.

Response: Thank you for this suggestion. We should note that it is not possible to provide a full example which considers an exact numerical calculation of the relationship between tidal forcing, response, and a given structure (as was done in this work) since this would include pre-processing scripts which account for the ephemerides of different bodies and are contained in the licensed (and non-distributable) gravity inversion code GEODYN. However, in place of these scripts, it is possible to provide an estimate of the amplitudes of the different forcings by analytically evaluating the prefactor (i.e., first line on the RHS of the Equation) of Equation 5 in the manuscript using keplerian orbital elements for Mars's orbit around the Sun. We have done exactly this, and find results which are essentially identical (<1% difference) to those calculated using the method presented in the manuscript for a trial interior model (the maximum likelihood solution across our inversion). We have uploaded the updated code to the DOI 10.5281/zenodo.20823473 (in Zenodo) and have updated the code availability statement of the manuscript text to reflect this change.

Referee #2

The authors provided a detailed response to the various points raised by reviewers regarding the first submitted version. The various additional tests carried out and included as Supplementary Information make the results more reliable and the interpretation of the shear modulus asymmetries deduced in terms of temperature variations and iron content is now more relevant. The implications for the thermal evolution of the Martian mantle, past volcanism and future measurements are also better argued. I have no further comments and I thank the authors for their detailed and thoughtful response and the careful revision of their manuscript, which is now suitable for publication in Nature.

Response: Thank you very much. We greatly appreciate the time taken to review the manuscript and to provide feedback on our work.
